# Long-Range and Coupled Rotor Dynamics in NO_2_-MIL-53(Al) by Classical Molecular Dynamics

**DOI:** 10.1021/acs.jpcc.4c05851

**Published:** 2024-11-12

**Authors:** Srinidhi Mula, Joris Bierkens, Louis Vanduyfhuys, Monique A. van der Veen

**Affiliations:** † Department of Chemical Engineering, Delft University of Technology, 2629 HZ Delft, The Netherlands; ‡ Delft Institute of Applied Mathematics, Delft University of Technology, 2628 CD Delft, The Netherlands; § Centre for Molecular Modeling, Ghent University, Technologiepark 46, 9052 Zwijnaarde, Belgium

## Abstract

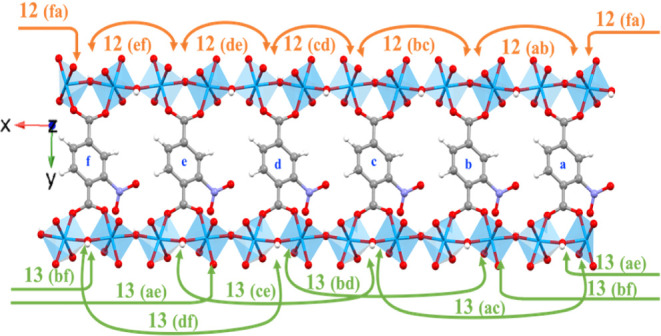

By tuning the steric environment and free pore space in metal–organic frameworks, a large variety of rotor dynamics of the organic linkers can appear. Nitrofunctionalized MIL-53 is a terephthalate-linker-based MOF that shows coupled rotor dynamics between the neighboring linkers along the pore direction. Here, we use classical molecular dynamics up to 6 × 2 × 2 supercells to investigate the range of the correlated linker dynamics. Interestingly, we observe an PNPNPNPN... conformational arrangement (P = nearly planar and N = nonplanar) for the conformations of the linkers in a row along the pore direction in the MOF. We identified correlated linker dynamics emerging among the direct and next nearest neighboring linkers along the pore. Due to 180° rotational flips of the planar linkers along the pore, (1) a change in the width of librations in their direct neighbors (PN) is observed; (2) intriguingly, their next nearest planar neighbors (PP) rotate between 0° and ±180° to reattain aligned (0°, 0°) or (±180°, ±180°) conformations. The presence of correlated dynamics in such linkers over long-length scales occurring at nanoseconds time scales is desirable for applications like ferroelectric switching or diffusion control via geared linker rotation, and this work provides insight into the design for such applications.

## Introduction

Metal–organic frameworks are hybrid materials composed of organic ligands that serve as linkers between the inorganic building units and are linked through coordination bonds. The organic linkers are often the flexible components in MOFs, and the rotational mobility of linkers is one of the major forms of flexibility seen in MOFs. The rotational dynamics of linkers in MOFs have a profound influence on their gas adsorption, diffusive properties, and optical properties.^1−5^ The different types of rotational dynamics that are observed in MOFs, their mechanisms, and the impact of such dynamics on MOF applications have been previously reviewed in detail in the work of Gonzalez-Nelson et al.^6^ Recent works on rotor MOFs aim to obtain ultrafast and free rotation of organic rotors in solid state that exist even at low temperatures.^7−11^ This is done by reducing the rotational energy barrier via molecular design and ample free space in the structure to avoid steric hindrance.

However, MOFs have the potential of displaying much more intricate dynamics in steric environments, similar to biological systems containing closely interacting molecules, such as crowded movement of proteins in lipid bilayers.^12,13^ Moreover, linker dynamics of MOFs play an important role for their application in artificial molecular machines.^14,15^ They provide a unique platform as they offer a regular arrangement of rotors with defined intermolecular distances that can be tuned through the choice of building blocks. This means that the available free pore space and interrotor distance can be tuned to a desired level of crowdedness of the rotors for achieving a cooperative motion. Such motion, when synchronized, can potentially be harnessed to impact diffusion through the pores.^16^ To fully realize the potential of MOF-based molecular machines and harness the collective motion of these rotors on the molecular scale, it is crucial to engineer the cooperative behavior of linkers through understanding the governing interactions on an atomic scale. This was previously studied for a molecular motor-functionalized MOF where the overall network topology of the MotorMOF is impacted due to the embedded motor–motor interactions.^17^ The essential design strategies for engineering gearing motion in crystalline solids was reviewed in the work of Liepuoniute et al.^18^ Specifically in supramolecularly arranged dirhodium paddle-wheel complexes, the effect of steric interactions on the rotational barriers and the time scales at which rotational dynamics occur was studied with various computational and experimental techniques.^19^ A recent work on a pillared paddle-wheel metal–organic framework presented the effect of steric interactions between the dipolar fluorinated terephthalate rotator and the pillaring linker on the rotational barrier of the former.^20^ Evidence of correlated motions of dipolar rotors through a cascade mechanism was observed in a MOF with a 2,2-difluorobicyclo[1.1.1]pentane-1,3-dicarboxylate linker. A very low rotational energy barrier was achieved, leading to correlated motions of dipolar rotors through a cascade mechanism, even at 2 K.^11^ In our previous work, the emergence of coupled linker dynamics was observed in the MIL-53 family by tuning the steric environment through functionalization of the terephthalate linkers. Specifically interesting is the case of NO_2_-MIL-53 with nitrofunctionalized terephthalate linkers. Complex rotational dynamics that evolve with temperature, spanning over a broad frequency range, were observed for the nitroterephthalate linkers, obtained using a combination of experimental techniques and ab initio molecular dynamics.^21^ The works on correlated linker motion in MOFs so far provide detailed insight into the interactions with the directly neighboring linkers, yet a complete understanding of correlated rotor dynamics requires probing also the long-range effects. The latter is indeed experimentally inaccessible and computationally requires the use of large supercells and thus the development of a force field for the specific system to balance computational cost and accuracy.

Here, we determine the length range of the correlated linker dynamics occurring in NO_2_-MIL-53. We developed a force field using QuickFF, which can predict the rotational linker dynamics for NO_2_-MIL-53, and carried out classical molecular dynamic simulations for supercells up to 6 × 2 × 2 unit cells. The free energy profiles and the long-range correlations between the orientation of neighboring linkers along the pore direction of the MOF are determined. Interestingly, we observe a long-range PNPNPN... conformational ordering of the P [Planar] linkers librating around 0° or ±180° [Planar], and N [Nonplanar] linkers librating between ±50° and ±120°. It is predominantly the planar linkers that are mobile through the 180°. Through a statistical analysis via a Markov model of the probabilistic subsequent rotations, we found that coordinated rotations occurred between the next nearest planar neighboring linkers to restore realignment to (0°, 0°) or (±180°, ±180°), typically with a time lag of a few nanoseconds. These insights into the longer-range correlated dynamics of sterically hindered rotors provide valuable insight into how to design rotor MOFs for applications based on cooperative linker dynamics, such as molecular motors and ferroelectric switching.

## Computational Methodologies

### Force Field Development

To be able to obtain the free energy profile and numerical analysis of rotational linker dynamics in NO_2_-MIL-53(Al), we need to reach simulation times and supercell sizes that are larger than what are typically obtainable with computationally expensive AIMD calculations. Therefore, for predicting the rotational linker dynamics in this MOF, we developed a force field (FF) using QuickFF protocol, a program that can be used for deriving force fields from ab initio inputs.^22,23^ The reason for the choice of using QuickFF among other available generic MOF-specific FF is that^24^ QuickFF was already used to derive a force field for unfunctionalized MIL-53, and this FF was also validated to correctly describe the geometry for MIL-53.^23^ Hence, we used QuickFF to generate the FF for NO2-MIL-53 and then refined it further to account for the full rotational pathway of the nitroterephthalate linkers. The force field energy consists of three contributions: analytical covalent, electrostatic, and van der Waals (vdW) force field terms. The covalent interactions are described in terms of bonds, bends (angles), torsions, out-of-plane distances, and cross terms that explicitly couple different internal coordinates in the structure. The parameters that figure in the analytical expression of the various covalent FF terms are fitted to reproduce the ab initio structure and the Hessian matrix in equilibrium as implemented in the QuickFF protocol.^22,23^ The electrostatic interactions are described by means the Coulomb interaction between Gaussian distributed atomic charges, which are estimated using the Minimal Basis Iterative Stockholder (MBIS) partitioning scheme.^25^ The vdW interactions are transferred from the MM3 model from Allinger et al.^26^ As we aim to use this FF for describing the dynamics of rotating linkers, we specifically focus further on the FF terms relevant to this motion. In the terephthalate linkers, the benzene rings rotate with respect to the carboxyl groups, and this is represented by a change in dihedral angles O_CA C_CA C_PC C_N and O_CA C_CA C_PC C_PH as indicated in Figure S1. In the force field generated with QuickFF, the torsion for these dihedrals is fitted on the deformation energies for small deviations from the equilibrium, as encoded in the Hessian. However, this does not account for full rotations of the linker that are of particular interest for this work, and it can be anticipated that the original QuickFF FF will not adequately capture the deformation energy along the full rotational pathway. Therefore, the force field was refined by refitting the dihedral contributions relevant for linker rotation. This is done by means of a rigid energy scan of the full rotational motion of the linker of NO_2_-MIL-53 in which the dihedral O_CA C_CA C_PC C_N was varied between −180° and 180°. Along this rotational trajectory, various energy contributions were calculated: the ab initio reference (AI) and the FF energy of the original QuickFF excluding the dihedral contributions that are to be refined (i.e., O_CA C_CA C_PC C_N and O_CA C_CA C_PC C_PH). Finally, the new dihedral contributions are obtained by fitting a Fourier series functional form to the difference in the energies between AI and FF. The final FF is obtained by simply replacing the original QuickFF dihedral contributions with the Fourier series resulting from the rotational fit. Detailed steps and the various software used at different steps in the force field generation and fitting procedures can be found in the Supporting Information.

### Definition of Regions Indicated in 2D Free Energy Surface Plots

**Figure 1:**
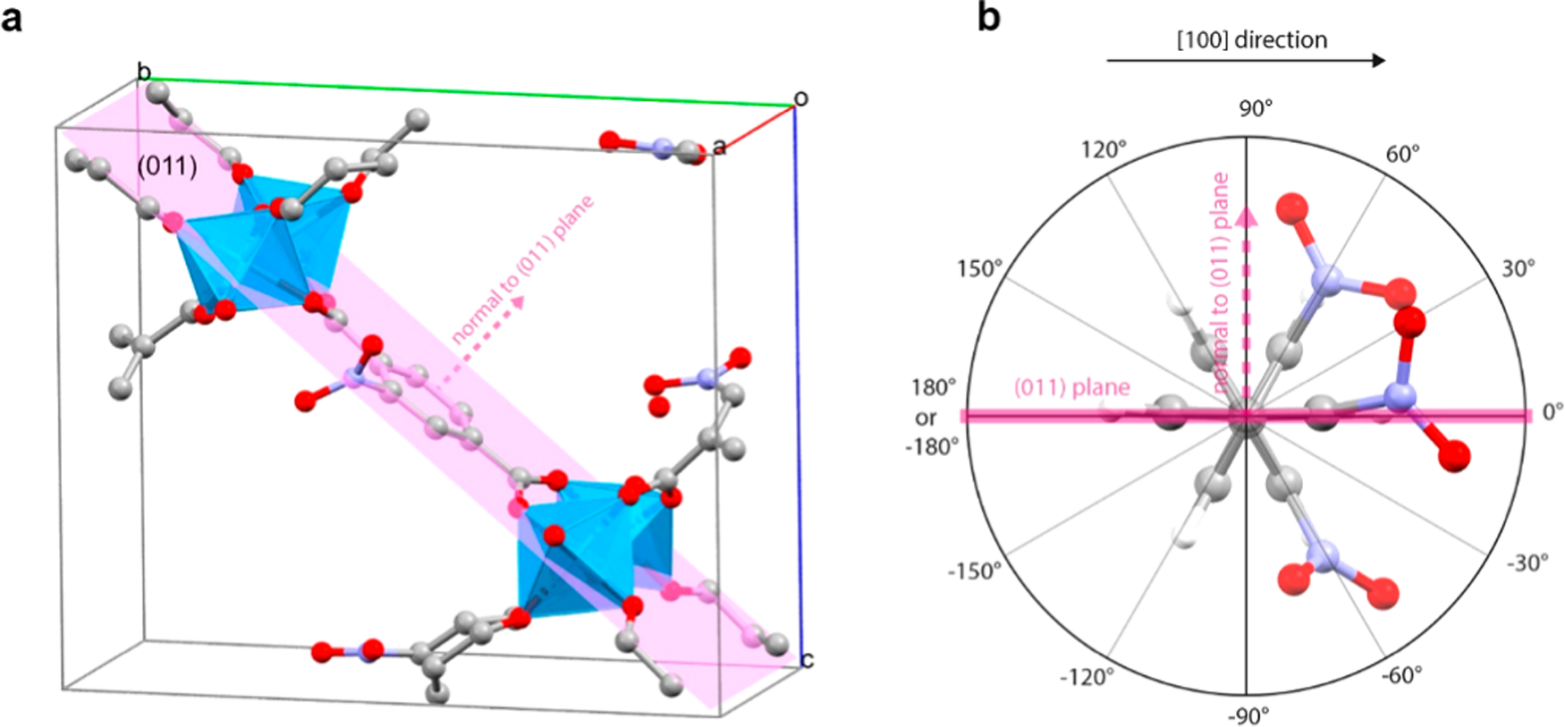
(a) Unit cell of NO2-MIL-53(Al) with central linker in 0° rotation with respect to the (011) plane (pink); hydrogens omitted for clarity. (b) Rotation angle is defined as the angle between benzene ring plane and (011) plane, taking 0° as the conformation with the functional group pointing in the positive [100] direction. The sign of the angle is assigned based on the direction normal of the reference plane. Reproduced from ref (21). Copyright [2021] American Chemical Society.

In order to evaluate the thermodynamic stability of the orientational state of the various linkers, we construct 2-dimensional free energy surface (FES) to describe the phase space for the orientation of direct neighboring and next neighboring linkers. Therefore, the rotation angle of nitroterephthalate linkers is defined as the angle between the benzene ring plane and the (011) plane, taking 0° as the conformation with the functional group in the positive [100] direction. The notation for the rotation angles used is shown in Figure 1. Multiple (unbiased) molecular dynamics simulations were performed from which the angle data are obtained using a python code from our previous work^21^ and used to construct the free energy surfaces (FES) using the ThermoLIB package.^27^ More specifically, the FES was constructed by combining the simulations through the weighted histogram analysis method (WHAM) in which the bias potential was set to zero.^28,29^

To allow for a transparent manner to refer to the various regions in the FES, we first introduce a specific notation. The *X* and *Y* axes in all 2D FES plots indicate the rotational angles of 2 pairs of linkers, either a pair of direct neighbors or a pair of next nearest neighbors. The regions in the FES plots are labeled into three types:

(1) Region PP, “Planar–Planar”: both linkers of the pair are close to planar (rotation angles close to 0° or ±180°).
(2) Region PN, “Planar–NonPlanar”: only one linker of the pair is close to planar.
(3) Region NN, “NonPlanar–NonPlanar”: both linkers of the pair are clearly nonplanar.

### Force Field (FF) Validation

To validate the accuracy of the obtained force field, we performed geometry optimization followed by normal-mode analysis to compute the normal-mode frequencies. We then compared the FF-predicted geometry and supercell dimensions with the experimental results as well as compared the FF-predicted normal-mode frequencies with the ab initio reference frequencies. The results are included in the Supporting Information and indicate good correspondence. Additionally, we also validated the newly developed force field for its ability of describing the linker dynamics by comparing with linker dynamics extracted from AIMD in our previous work.^21^ The FES plots obtained with AIMD and FF simulations for a 2 × 1 × 1 supercell at various temperatures are discussed in detail in the Supporting Information. Overall, we observe in the free energy profiles obtained both via AIMD and FF that the linkers have specific preferred orientations. At 300 K, we see that the “Planar–NonPlanar” (PN) configuration for the sets of neighboring linkers is dominant in both AIMD and FF simulations, showing that they are in agreement (Figures S6 and S8). The evolution of the free energy profiles with the temperature in FF is consistent with that of AIMD (Figures S7 and S9). With an increase in thermal energy, the “Planar–NonPlanar” (PN) configuration for the linkers becomes unfavorable and the “NonPlanar–NonPlanar” (NN) configuration is increasingly seen. The difference between AIMD and FF FES plots is that FF underestimates the energy associated with the orientation of a linker at ±90°. So, there is a difference in the location of the PN minima region between AIMD and FF simulations, especially the N angles. In FF simulations, we see a single broad conformational distribution for the PN regions with the N angle ranging from ±50° to ±130°, whereas in AIMD, two narrow distributions that are centered at ±50° and ±135° are observed. It is also important to note here that the simulation time for AIMD was much shorter than the FF MD simulations (40 ps versus 1 ns). This would mean that the sampling in AIMD is much less reliable than that in FF, which could be the reason for the appearance of narrow minima in AIMD FES (Figure S6). Moreover, the region that is most energetically unfavorable is the same in AIMD and FF mainly entailing neighboring linkers at (0°, 0°), (±180°, ±180°), (0°, 0°), (±180°, 0°), (0°, ±180°), (90°, −90°), and (−90°, 90°) conformational angles. With this in consideration, we do not make conclusions based on the magnitude of nonplanar angles (N) in PN but only on the type of energy minima regions in the FES plots (i.e., PN or NN).

## Results and Discussion

Using the newly developed and validated FF, we performed MD simulations for larger simulation times and supercells of 4 × 2 × 2 and 6 × 2 × 2 supercells. In the 4 × 2 × 2 and 6 × 2 × 2 supercell, there are 16 AlOH chains with respectively four and six rotating linkers per chain along the pore direction, thus 64 and 96 linkers, respectively (see Figures S10 and S11 for the 4 × 2 × 2 supercell). The data of all of the linkers over the entire simulation time of 70 ns are included in the calculations of the different free energy surfaces. Note that, we only discuss here in detail interactions and correlations between linkers along the pore direction (along *x*-axis) in a single chain of linkers rather than neighboring interactions between four other neighbors perpendicular to the pore direction (along *y* and *z* axes). Based on a qualitative assessment of the time traces of neighboring linkers along the *y* and *z* axes, we conclude that no meaningful correlations persist between the linkers across the pore (see the Supporting Information).

### 4 × 2 × 2 Supercell

**Figure 2:**
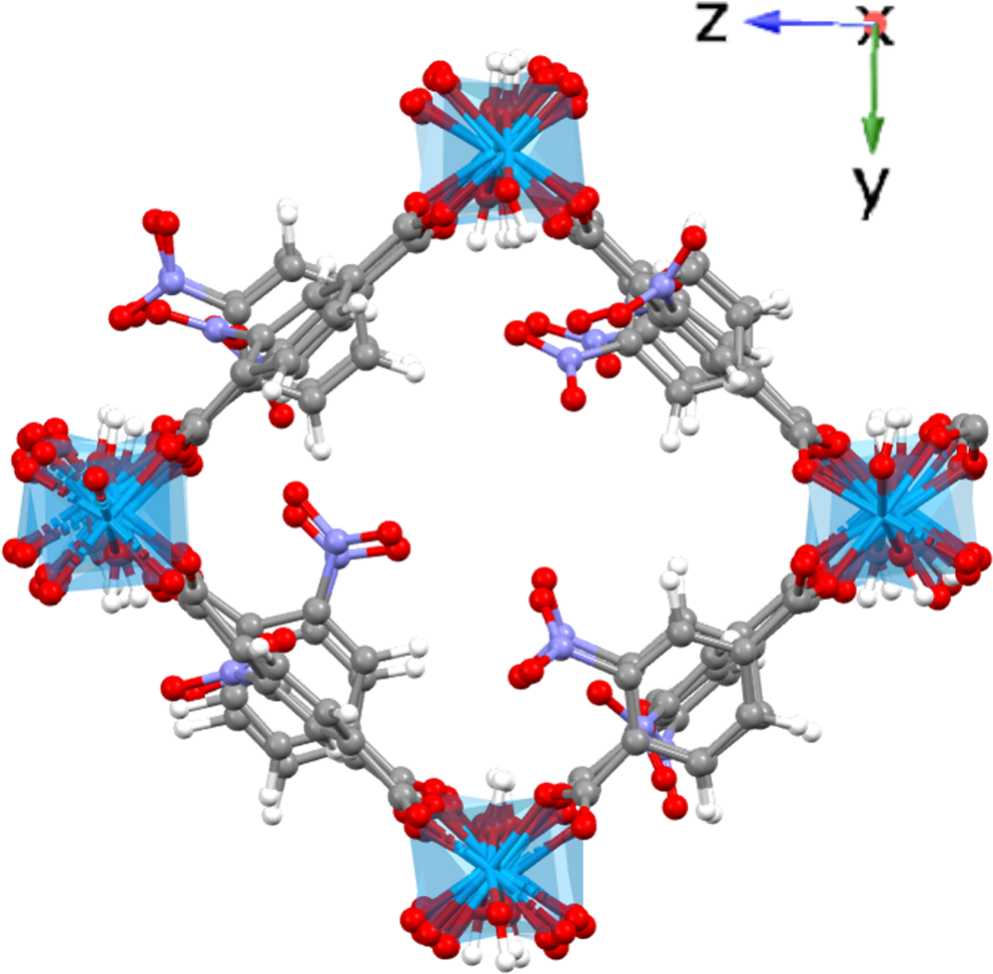
Part of the 4 × 2 × 2 supercell of NO_2_-MIL-53 MOF, i.e, single pore of the 4 × 2 × 2 supercell consisting of four chains of linkers.

**Figure 3:**
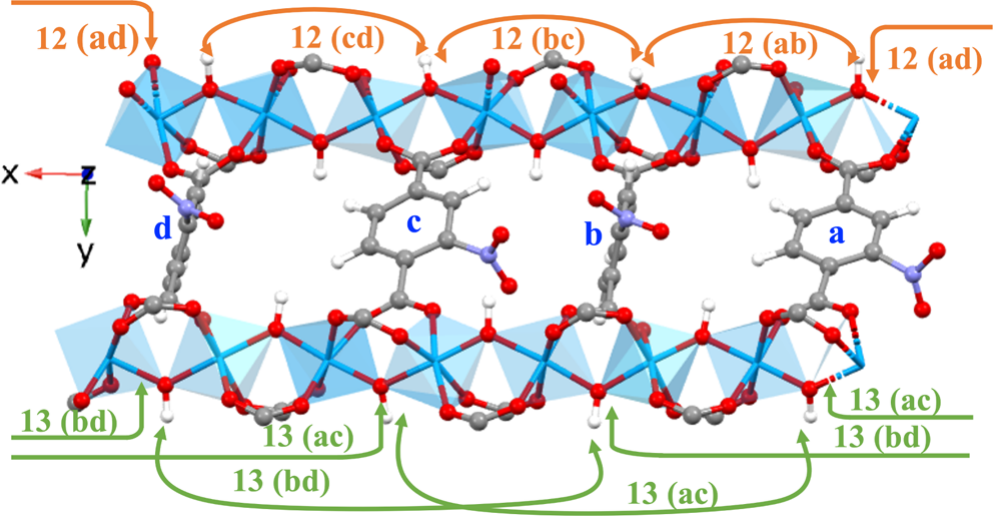
Representation of direct neighbors “1, 2” type (orange: includes pairs ab, bc, cd, ad) and alternate neighbors “1, 3” type (green: includes pairs ac, bd) in a single chain along the pore direction (*x*) for a 4 × 2 × 2 supercell.

The MD simulation for a 4 × 2 × 2 supercell was performed at a temperature of 300 K. Figure 2 shows a scheme of a single pore in the supercell showing four chains of linkers, and Figure 3 shows the types of direct neighboring “1, 2” (linker pairs ab, bc, cd, ad) and next neighboring “1, 3” (linker pairs ac, bd) interactions along the pore direction. We show in the results two different kinds of free energy surface (FES) plots, namely in terms of the rotational angles of the direct neighbors (“1, 2” type) and of the next nearest neighbors (“1, 3” type), as well as time traces of the rotation angle for all four rotating linkers along the pore direction.

#### Free Energy Surfaces of “1, 2” and “1, 3” Type Neighbors

**Figure 4:**
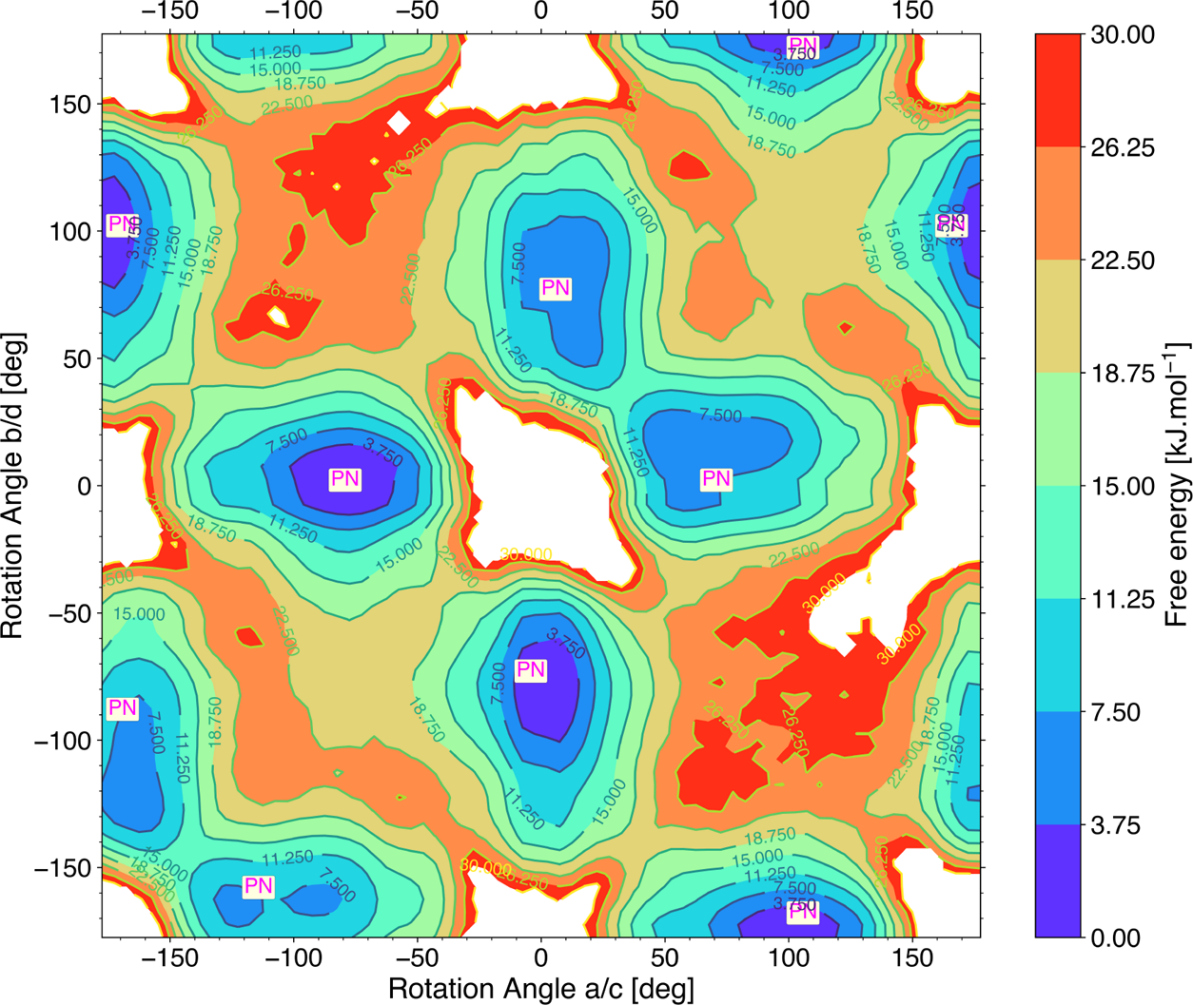
Free energy surface (FES) for a 4 × 2 × 2 supercell in terms of the rotational angles of “1, 2” type direct neighbors. The white regions indicate unsampled rotational conformations in the MD simulation.

**Figure 5:**
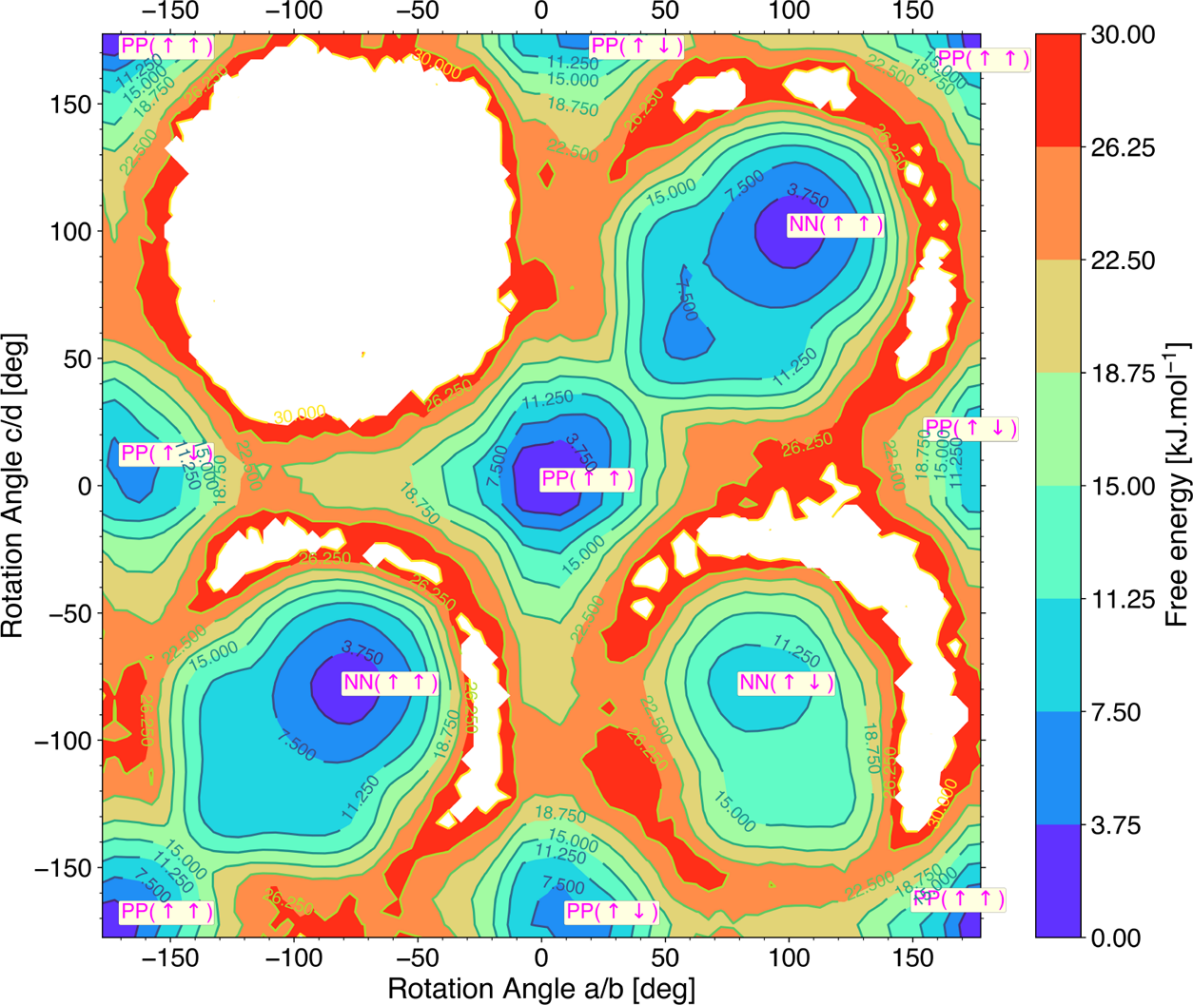
Free energy surface (FES) for a 4 × 2 × 2 supercell in terms of the rotational angles of “1, 3” type next nearest neighbors. The white regions indicate the unsampled rotational conformations in the MD simulation.

The free energy surfaces (FES) of the 4 × 2 × 2 supercell in Figures 4 and 5 give an overall picture of the linkers’ orientation. In Figure 4, where the FES is plotted in terms of the orientations of “1, 2” neighbors as collective variables, the minima regions are only of PN type where one linker is planar, and the other linker is nonplanar. We do not see PP and NN regions as minima for the 4 × 2 × 2 supercell at 300 K. NN regions only occur as transition states between two adjacent PN regions (for example, between (0°, 80°) and (80°, 0°) in Figure 4) with a free energy of around 11.25–15 kJ/mol. Note that in the 2 × 1 × 1 supercell FES (Figure S8), we do not see PP regions as minima, and it is dominated by PN minima regions. We do observe a few NN regions in both AIMD and FF plots. This could be an effect of the smaller supercell, which imposes restrictions onto the orientational freedom of the linkers due to the periodic boundary conditions. More specifically, next neighboring linkers are forced to orient identically and hence heavily influences the orientational dynamics.

Upon shifting our attention to Figure 5 for the FES in terms of the orientations of “1, 3” type neighbors, we observe that the global minima are now located along the diagonal and corners of the plot (the dark blue regions) with an energy up to 3.75 kJ/mol. At these minima, the “1, 3” neighbors are librating around the same kind of rotational angle, i.e., both linkers are either planar and aligned parallel PP(↑↑) or both linkers are nonplanar and aligned parallel NN(↑↑). Next to this global minima, we also observe less deeply bound local minima (i.e., light blue regions) for the “1, 3” type of linker pairs. These represent either antiparallel aligned planar linkers (i.e., PP(↑↓)) with a free energy in the range of 3.75–7.50 kJ/mol or antiparallely aligned nonplanar linkers (i.e., NN(↑↓)) with a slightly higher free energy up to 11.25 kJ/mol. Antiparallely aligned linkers are linkers with rotational angles that are 180° apart conformations. Note that in Figure 5, there is a region, i.e., for a/b = −150° to 0° and c/d = 30° to 180°, that is not sampled in the MD simulations of 4 × 2 × 2 supercell. This is due to the high free energy (i.e., the red barrier) surrounding it and could be addressed by means of applying a bias potential as is commonly done in Umbrella Sampling. However, this region is symmetrically equivalent, because of the interchangeability of linker conformation, to the region of a/b = 30° to 180° and c/d = −150° to 0° (and hence NN(↑↓)) that was sampled. Therefore, we conclude that the barrier is high but not too high, resulting in only sporadic transitions to the NN(↑↓) region, which in turn results in one region begin sampled while its equivalent is not. To summarize, from the FES plots of the linkers along the row, i.e., linker a, linker b, linker c, and linker d, direct or “1, 2” type neighbors have different conformations P and N where one linker is planar (0° or ±180°) and the other linker is nonplanar. The next nearest or “1, 3” type neighbors are librating around the same conformation, i.e., P and P or N and N while parallel (PP(↑↑), NN(↑↑)) or antiparallel aligned (PP(↑↓),NN(↑↓)), with parallel alignment being energetically slightly more favorable. As a result, the configuration of linkers along a chain is abcdabcd... = PNPNPNPN... (or NPNPNPNP...).

#### Effect of Change in the Rotational Angle of Linkers on its Neighbors

**Figure 6:**
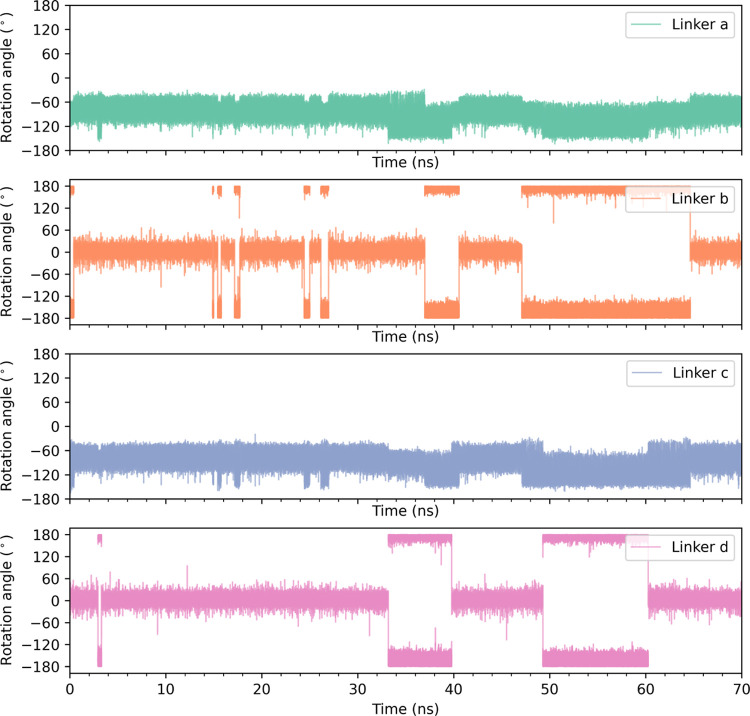
MD trajectory of the four linkers along the row for a specific chain in the 4 × 2 × 2 supercell.

Now that we investigated the thermodynamic stability of the various configurations of the linkers, we shift our focus to the rotational dynamics and more specifically to correlations between linker rotations. To this end, we consider the time traces of the rotational angles of the linkers (a, b, c, d) within a single chain as shown in Figure 6. Herein, we observe frequent rotation of linkers b and d between two planar conformations, i.e., 0° and ±180°, which we will now analyze in more detail. This 180° rotational flip of planar linkers occurs over a couple of picoseconds, and this transition requires overcoming a free energy barrier of ∼24 kJ/mol per each linker, as seen from the zoomed in MD trajectory and illustrative path on the FES in Figure S16b.

##### “1, 2” Type Interactions

**Figure 7:**
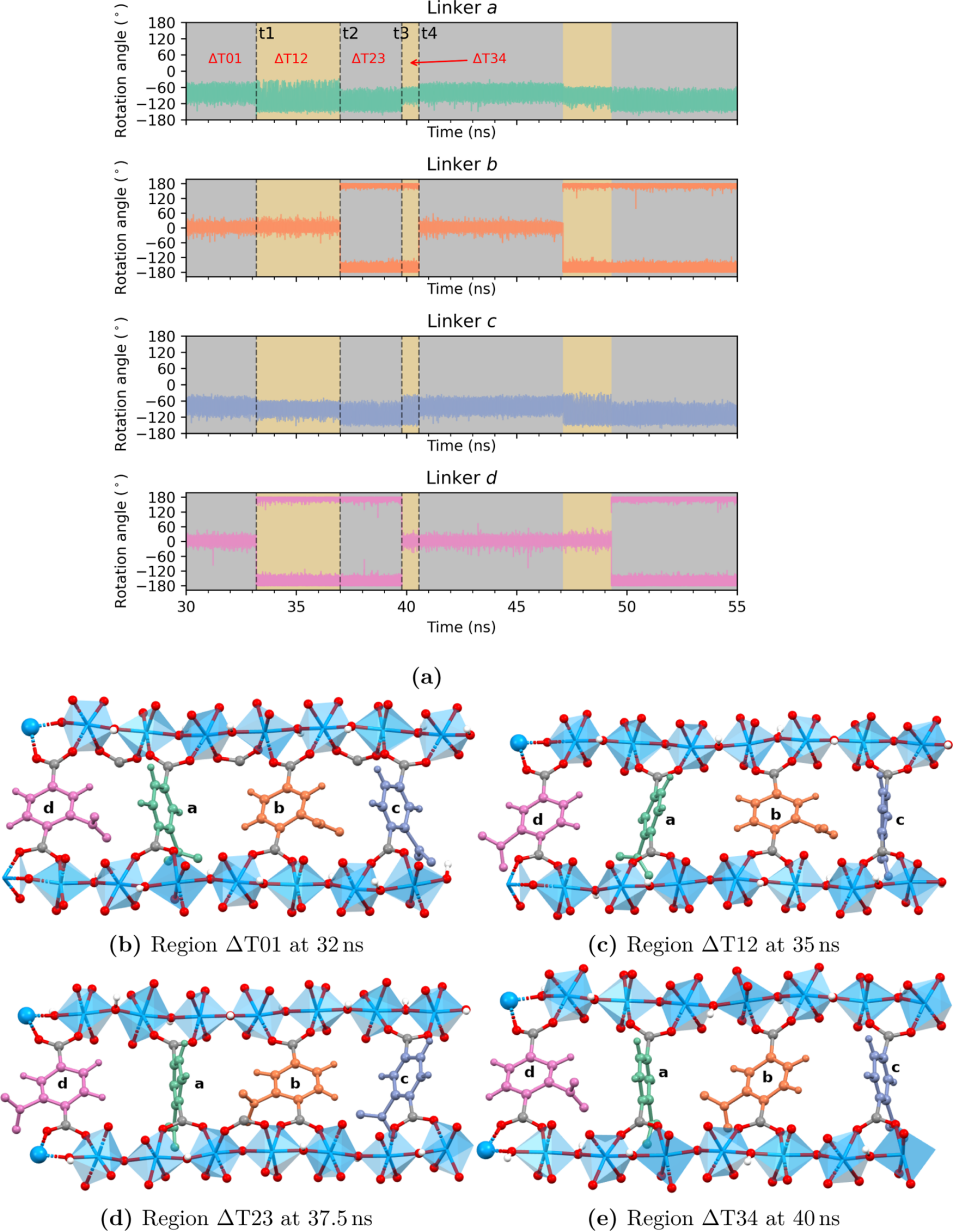
(a) MD trajectory of linkers highlighting the changes in rotational angle flips of 180° in their neighboring linkers for a specific chain in the 4 × 2 × 2 supercell. (b–e) Conformations of all of the four linkers in the chain in regions ΔT01, ΔT12, ΔT23, and ΔT34 at specific times highlighted in the MD trajectory.

Figure 7a shows the time trajectory from 30 to 55 ns of the same chain as shown in Figure 6. The time steps where linkers b and d are in the same conformation (parallel (↑↑) aligned states: (0°, 0°) or (±180°, ± 180°)) are highlighted in gray, and where they are 180° apart (antiparallel (↑↓) aligned states: (0°, ± 180°) or (±180°, 0°)) are highlighted in gold. In general, for parallel (↑↑) aligned states of linkers *b* and *d* (in ΔT01 and ΔT23), the widths of the librations for linkers a and c are equal but possibly be slightly shifted in average value (i.e., shifted in ΔT23 w.r.t ΔT01). For antiparallel (↑↓) aligned states of linkers b and d (in ΔT12 and ΔT34), the linkers *a* and *c* have either wider (i.e., a in Δ*T*12 and i.e., c in ΔT34) or narrower librations (i.e., c in ΔT12 and i.e., a in ΔT34). The overall picture observed in Figure 7a shows linker dynamics representative of all of the linkers in the 4 × 2 × 2 supercell, as was confirmed by considering all other chains (see the Supporting Information). Starting in region ΔT01, linkers b and d are at the same conformations (gray color) in a parallel (↑↑) aligned state centered at 0° and linkers a and c are librating in nonplanar conformations. A snapshot of the positions of four linkers in region ΔT01 is shown in Figure 7b. At t1, linker d flips to a ± 180° conformation leading to an antiparallel (↑↓) alignment of linkers b and d. At the same time, the width of the librations change in its direct neighbors linkers a and c (“1, 2” type). The width of the librations becomes wider in linker a (from [−40°,–110°] to [−40°,–150°]) and narrower in linker c (from [−40°,–110°] to [−60°,–120°]). This is related to the direction of the nitro group in linker d before (in ΔT01) and after (in ΔT12) the 180° flip at t1. In region ΔT01, linker c has only one nitro group pointing toward it (from linker b, see Figure 7b), whereas in region ΔT12, it has two nitro groups (from linkers b and d, see Figure 7c) pointing toward it; hence it is forced to librate narrowly yet more symmetrically around −90° than in region ΔT01. For linker a, in region ΔT01, there is one nitro group pointing toward it (from linker d, see Figure 7b), while in region ΔT12, there are no nitro groups pointing toward it (see Figure 7c), thus it is allowed more freedom resulting in wider librations than in region ΔT01.

At t2, linker b rotates to a ±180° conformation and both linkers b and d are now again in parallel (↑↑) aligned state. In region ΔT23, the width of librations for linkers a and c become equal, as at of ΔT01. However, in ΔT23 (rotating angle between −70° and −150°), their average rotational angle is shifted compared to the average angle in ΔT01 (rotating angle between −50° and −120°). This is due to the change in position of the nitro groups of linkers b and d; pointing toward right in ΔT01 (Figure 7b) and toward left in ΔT23 (Figure 7d).

##### “1, 3” Type Interactions

The FES of next neighbor or “1, 3” type neighbors in Figure 5 indicates that they are librating around the same rotational angle, i.e, both are in planar conformations (PP (↑↑)) or both are in nonplanar conformations (NN(↑↑)). On looking at various time trajectories for all of the 16 chains in the 4 × 2 × 2 supercell, “1, 3” type linkers in planar conformations rotate between the two planar conformations i.e, 0° and ±180° as frequently seen, in Figure 6 where linkers b and d flip between 0° and ±180°. In Figure 7a, following the time trajectory from 30 ns, linker d (linker “1” type) rotates from 0° to ±180° at ∼33 ns, which is then followed by rotation of linker b (linker “3” type) at ∼37 ns; so a time gap of ∼4 ns. Again at ∼39.8 ns, linker d rotates back to 0° followed by linker b rotating back to 0° at ∼40.8 ns; a time gap of ∼1 ns. Hence, for the “1, 3” type neighbors, we not only see frequent flips between the planar conformations but also the linker “3” type follows the linker “1” type, or vice versa. Alternatively, the parallel (↑↑) aligned state is restored when the linker that rotated to an unaligned state rotates back to its original conformation, as can be seen in Figure S17 (linker b and d). Overall, it appears that transitions from (↓↑) to (↑↑) (or ↓↓, which is energetically equivalent) appear on a shorter time scale than vice versa. Such kinetics is consistent with the observation from the FES of “1, 3” neighbors as shown in Figure 5, where parallel aligned states (PP(↑↑)) are the global minima and antiparallel aligned states (PP(↑↓)) are less deeply bound local minima with a difference in free energy up to 7.50 kJ/mol. This energy difference is most probably due to slightly increased electrostatic repulsion experienced by the linker squeezed between the two NO_2_ groups of the two neighboring planar linkers at an antiparallel conformation of 0° and ±180° (as shown in Figure 7e). This delayed yet coordinated rotations between “1, 3” type neighbors in NO_2_-MIL-53(Al) is a probabilistic mechanism that happens over a time delay on the nanosecond scale. A statistical analysis of these coordinated rotations in “1, 3” type linkers is presented in the next section. For the “1, 3” type nonplanar linkers centered around (−80°, −80°) or (100°, 100°) rotational flips of 180° would mean changing to (−80, 100°) or (100°, −80°). This is a very rare event observed in the MD trajectories and could be due to the higher energy of these regions (NN(↑↓)) up to 11.5 kJ/mol compared to the minima NN(↑↑). In all of the modeled trajectory for all of the chains in the 4 × 2 × 2 supercell, 180° rotational flips of nonplanar linkers occurred only once, versus 180° flip of a planar linker at least 20 times.

### 6 × 2 × 2 Supercell

To simulate structures closer to the experimental crystal structures, we increase the length of the supercell to 6 × 2 × 2 with six rotating linkers per MOF chain along the pore direction (as illustrated in Figure S19). Similar to the 422 supercell described previously, the rotational angle data obtained from the MD simulations of 6 × 2 × 2 supercell were used to plot the free energy surface plots for “1, 2” type neighbors (includes linker pairs ab, bc, cd, de, ef, fa), see Figure S20 and “1, 3” type neighbors (includes linker pairs ac, ae, bd, bf), see Figure S21. The FES plots of the 6 × 2 × 2 supercell are qualitatively and quantitatively identical to that of the 4 × 2 × 2 supercell. The type of minima regions (i.e., PN) and location of minima in the case of both “1, 2” and “1, 3” type neighbors for the 6 × 2 × 2 supercell is the same as that of 4 × 2 × 2 supercell. From the FES plots (Figures S20 and S21) of various different chains in the 6 × 2 × 2 supercell, we observe each chain is always in the configuration abcdef = PNPNPNPNPNPN..., identical to our observations for a 4 × 2 × 2 supercell.

Based on the time trajectories of the different chains in the 6 × 2 × 2 supercell (Figure S22), we observe linkers in planar conformations rotating between the two planar conformations, i.e., 0° and ±180°. Because of these 180° rotations, wider and narrower librations are observed in their “1, 2” neighbors, identical with the observations for the 4 × 2 × 2 supercell. The planar “1, 3” linker pairs are switching between parallel aligned and antiparallel aligned states by coordinated rotations occurring at the time scale of nanoseconds, with transitions that restore an aligned PPP (↑↑↑) or PPP(↓↓↓). Thus, the “1, 2” and “1, 3” type interactions in the 6 × 2 × 2 supercell are similar to the interactions observed for a 4 × 2 × 2 supercell. To allow for a more quantitative comparison, a statistical analysis of these “1, 3” type probabilistic coordinated rotations occurring in planar linkers is shown in the next section. A more detailed description of the FES plots and representative time trajectories for the 6 × 2 × 2 supercell is included in the Supporting Information.

### Statistical Analysis of Correlated Rotations of “1, 3” Type Planar Linkers

So far, we extracted the thermodynamic properties of the rotational motion in linkers (FES in Figures 4 and 5) from the probability distribution of the conformations of each linker. To derive the kinetic properties, a statistical analysis of the probabilistic rotations occurring in “1, 3” type planar linkers was done to estimate the transition rates from a parallel aligned state to an antiparallel aligned state and vice versa. Other ways to compute the rate constant is from the free energy barrier within the approximation of transition state theory (TST).^30^ For this, additional information about the speed at which the linker rotates in the transition state is required that can be obtained through enhanced sampling techniques such as umbrella sampling. Such computationally expensive simulations are beyond the scope of the current work.

The statistical analysis was done using the Expectation-Maximization (EM)-algorithm^31,32^ to estimate the transition rates in a Markov model from a parallel aligned state to an antiparallel aligned state and vice versa, taking into account uncertainty due to librations. Additional details of the mathematical model used for the analysis are described in the Supporting Information. As before, “1, 3” type linkers are considered parallel aligned PP(↑↑) if the conformations of linkers “1” and “3” type are in the same planar conformations (i.e., (0°, 0°) or (±180°, ± 180°)) and are considered antiparallel aligned PP(↑↓) if they are 180° apart planar conformations (i.e., (0°, ± 180°) or (±180°, 0°)). In a 4 × 2 × 2 supercell, there are two “1, 3” type planar linkers; hence, two transition states are possible:

(1) Parallel aligned state, i.e., (0°, 0°) or (±180°, ± 180°), to antiparallel aligned state, i.e., (0°, ± 180°) or (±180°, 0°), which we denote as P to AP.
(2) Antiparallel aligned state to parallel aligned state, which we denote as AP to P.

Similarly, in a 6 × 2 × 2 supercell, there are three “1, 3” type planar linkers and three transition states are possible:

(1) Fully parallel aligned to antiparallel aligned (P to AP), e.g., (0°, 0°, 0°) to (0°, 0°, ±180°).
(2) One antiparallel aligned state to another antiparallel aligned state (AP to AP), e.g., (0°, 0°, ±180°) to (0°, ±180°, ±180°).
(3) From antiparallel aligned state to parallel aligned state (AP to P), e.g., (0°, ±180°, ±180°) to (±180°, ±180°, ±180°).

**Figure 8:**
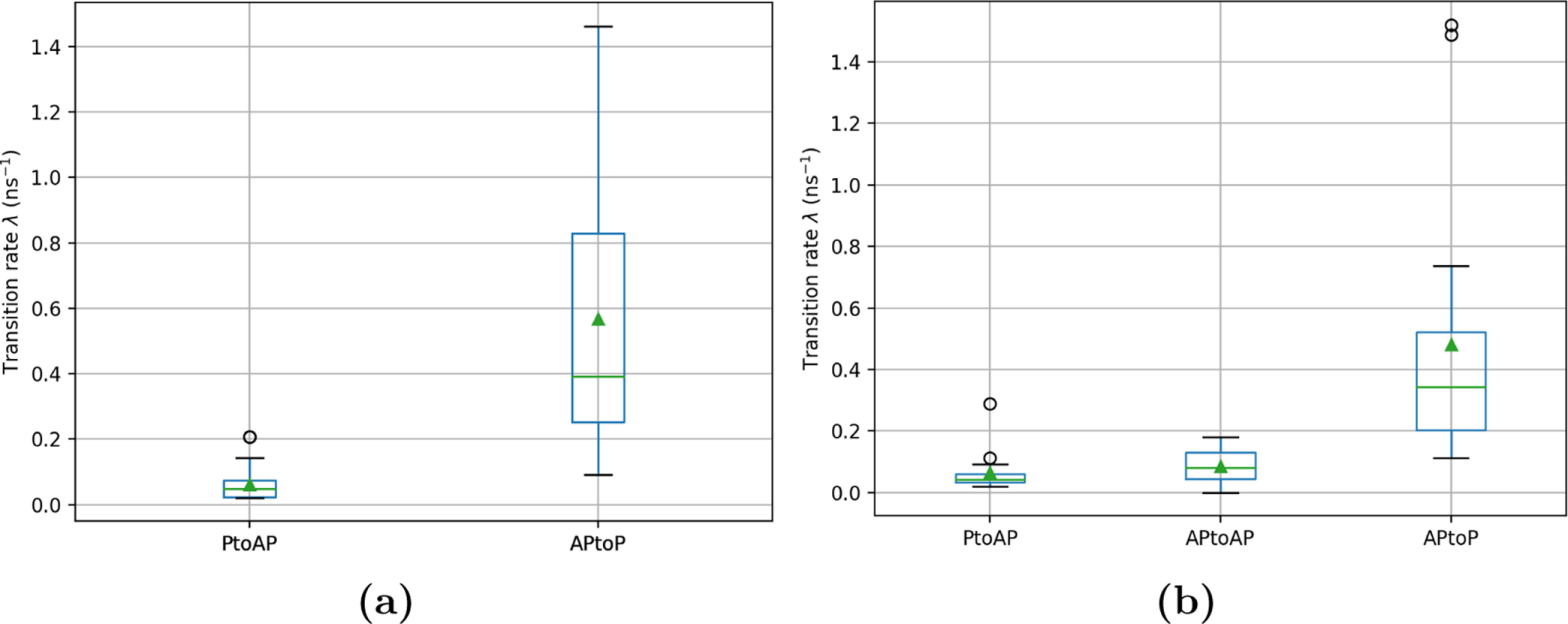
Distribution of transition rates λ from parallel to antiparallel state and vice versa occurring in “1, 3” type planar linkers in 4 × 2 × 2 and 6 × 2 × 2 supercell.

The transition rates (λ) for “1, 3” type neighbors in all 16 chains of the supercell are extracted from the rotational angle data of the MD simulations. From those values, the mean, median, and 95% confidence interval for the rate of the transition from an antiparallel state to a parallel state (λ_AP to P_) and parallel state to an antiparallel state (λ_P to AP_) for 4 × 2 × 2 and 6 × 2 × 2 supercells are reported in Table 1 and plotted in the box plot (Figure 8a,b). Additionally, the transition rate from an antiparallel state to another antiparallel state (λ_AP to AP_) occurring in a 6 × 2 × 2 supercell is also included. As reported in Table 1, the transition rates (λ) for a 6 × 2 × 2 supercell are lower than those for a 4 × 2 × 2 supercell. Due to the possibility of more linker combinations and more degrees of freedom in 6 × 2 × 2 supercell when compared to a 4 × 2 × 2 supercell, we estimate that the transition rates of 6 × 2 × 2 supercell are more reliable and closer to the absolute values. In both supercells, the mean and median transition rates λ_AP to P_ are about an order of magnitude higher than λ_Pt o AP_: for the 6 × 2 × 2 supercell the median λ_AP to P_ = 0.344 ns^–1^ versus λ_P to AP_ = 0.042 ns^–1^, and the median λ_AP to P_ = 0.344 ns^–1^ is also about an order of magnitude higher than λ_AP to AP_ = 0.082 ns^–1^. The rate of transition from an antiparallel aligned state to a parallel state is faster than that vice versa, indicating that antiparallel aligned linkers experience a driving force to return to a parallel aligned state. These observations are also in line with the observations from the free energy surface plots where “1, 3” type neighbors in parallel aligned states have lower free energy (more favorable conformations) than antiparallel aligned states or 180° apart conformations.

**Table 1:** Transition Rates (λ) Obtained from Statistical Analysis of 180° Rotations Occurring in “1, 3” Type Planar Linkers

	4 × 2 × 2 supercell		6 × 2 × 2 supercell		
	λ_P to AP_	λ_AP to P_	λ_P to AP_	λ_AP to AP_	λ_AP to P_
mean λ (ns^–1^)	0.062	0.568	0.064	0.085	0.481
median λ (ns^–1^)	0.048	0.391	0.042	0.082	0.344
standard deviation (SD) of λ (ns^–1^)	0.051	0.441	0.065	0.060	0.437
confidence interval (95%) for λ (ns^–1^)	0.036–0.087	0.347–0.788	0.032–0.097	0.055–0.115	0.262–0.699

In order to get a more general picture of the time scale required for the transitions, we consider the box plots Figure 8a,b. The overall range for transition rate λ_AP to P_ for transitioning from antiparallel to parallel alignment as seen from the box plot is 0.08 to 1.46 ns^–1^ in a 4 × 2 × 2 supercell and 0.1 to 0.73 ns^–1^ for a 6 × 2 × 2 supercell. As such, we find the APtoP transitions to typically occur on the time scale of 1–10 ns. The correlated rotational behavior of the next nearest or “1, 3” type linkers can be summarized as follows. From the FES in Figures 5 and S21, we observe the “1, 3” type linkers are librating around the same conformations (i.e., PP or NN). The planar linkers in the system are rotating between 0° and ±180° conformations, and that would mean the “1, 3” type linkers together are switching between parallel PP(↑↑) and antiparallel PP(↑↓) aligned states. The parallel states PP(↑↑) are energetically more favorable than the antiparallel states PP(↑↓), and the system is in a metastable state in the antiparallel state. Hence, the switching time from an antiparallel state to a parallel state is occurring over a shorter time scale of 1–10 ns. This occurs either by returning the rotated linker to its original state or by also rotating its next neighbors over 180°. With experimental techniques such as broadband dielectric spectroscopy (BDS) and solid-state deuterium NMR (^2^H SSNMR), the time scales (ns) relevant for the dynamics of the linkers are indeed accessible. This was previously investigated in our previous paper for the same MOF i.e., NO_2_-MIL-53.^21^ However, with these experimental techniques, only the aggregate behavior of all of the linkers in the MOF can be observed and the precise type of interactions, i.e., “1, 2” and “1, 3” type and the long-range effects of these interactions cannot be observed.

## Conclusions

By means of molecular simulations, we constructed the free energy surface and investigated the correlated dynamics for linker rotations in the NO_2_-MIL-53. To this end, we first developed and validated a force field that can predict the linker dynamics in this MOF. Using classical MD simulations for 4 × 2 × 2 and 6 × 2 × 2 supercells and simulation times up to 70 ns, we observe a distinct PNPNPNPN... arrangement for the linkers along the pore direction where P refers to a planar linker with librations around 0° or ±180° and N refers to a nonplanar linker with librations between ±50° and ±120°. The direct neighbors are always in “Planar–NonPlanar” conformations, whereas the next nearest neighbors are in either in “Planar–Planar” or “NonPlanar–NonPlanar” conformations. In its thermodynamically most stable state, all P-linkers are furthermore parallel aligned, i.e., all in 0° or all in ±180°. However, due to 180° rotational flips of the planar linkers, the system can transition into a metastable antiparallel aligned PNPNPNPN... configuration. The estimated free energy barrier for this initial 180° flip is ∼24 kJ/mol per linker at 300 K. In this metastable intermediate state, the system is driven back to the global minimum through a similar 180° rotational flip of a next neighbor on a time scale of 1–10 ns. Due to these 180° flips occurring in planar linkers, we see correlated linker dynamics emerging in the direct neighbors and the next nearest neighbors along the pore direction in the same chain. With regard to the direct neighbors, the librations of the nonplanar linkers become wider or narrower based on the direction of the nitro group neighboring planar linkers. The planar next nearest neighbors are rotating between parallel aligned [(0°, 0°), (±180°, ±180°)] and antiparallel aligned [(0°, ±180°)] state, where the parallel aligned state is recovered on a shorter time scale compared to the manifestation of the antiparallel aligned state. This work provides for the first time detailed insight into the intricate correlated linker dynamics of Rotor MOFs. As such, it provides understanding for the design of Rotor MOFs for applications based on correlated linker dynamics like ferroelectric switching or diffusion control via geared linker rotation.

## Data Availability

Raw data and files used during different steps of force field generation, force field validation, and input, output files, and python scripts used for LAMMPS molecular dynamics of 4 × 2 × 2 and 6 × 2 × 2 supercells are available in the 4TU data repository at 10.4121/08ed7ce7-fd71-4ace-a671-82a0a379c902.

## Supporting Information Available

Detailed steps in force field development and validation, supporting MD results of “1, 3” type neighbor interactions, results of 6 × 2 × 2 supercell and model description of statistical analysis. (PDF)
Force field generated in this work. (TXT)
MD time trajectory plots of all of the 16 chains in the 4 × 2 × 2 and 6 × 2 × 2 supercells. (ZIP)
Transition rates of “1, 3” type planar linkers in each chain for 4 × 2 × 2 and 6 × 2 × 2 supercell obtained through statistical analysis. (XLSX)
Video showing “1, 3” and “1, 2” type linker interactions for a single chain of linkers. (MP4)
