# Supplementary material for: Long-Range and Coupled Rotor Dynamics in NO2-MIL-53(Al) by Classical Molecular Dynamics
Source: J Phys Chem C Nanomater Interfaces. 2024 Nov 12;128(47):20264–74. doi: 10.1021/acs.jpcc.4c05851 (PMC11613561; doi:10.1021/acs.jpcc.4c05851)
Supplement: Supplementary file 1 — jp4c05851_si_001.pdf [file jp4c05851_si_001.pdf]

Supporting Information

Long-Range and Coupled Rotor Dynamics in NO_2-MIL-53(Al) by Classical Molecular Dynamics

Srinidhi Mula,[†] Joris Bierkens,[‡] Louis Vanduyfhuys,[*,ℙ] and Monique A. van der Veen[*,†]

†*Department of Chemical Engineering, Technische Universiteit Delft, Delft, 2629HZ, The Netherlands*

‡*Delft Institute of Applied Mathematics, Delft University of Technology, 2628CD Delft, the Netherlands*

ℙ*Centre for Molecular Modeling, Ghent University, 9052 Zwijnaarde, Belgium*

E-mail: Louis.Vanduyfhuys@UGent.be; m.a.vanderveen@tudelft.nl

Force Field Development

QuickFF program is used to derive force fields for metal–organic frameworks from ab initio data of the equilibrium structure as input (`http://molmod.github.io/QuickFF/index.html#`). The energy expression for the force field including covalent and non-covalent interactions used in QuickFF is:

$$V^{FF} = \underbrace{V_{bond} + V_{bend} + V_{oopd} + V_{torsion} + V_{cross}}_{V_{cov}} + \underbrace{V_{ei} + V_{vdW}}_{V_{noncov}} \tag{1}$$

The functional form for each contribution in equation 1 is mentioned in previous literature on development of QuickFF program.[1] Specific to this work, the methodology for obtaining the

force field is described below. The general steps involved with FF development for predicting the rotational linker dynamics is summarized below.

Step 1: Generation of ab initio (AI) data for input to QuickFF program

1. **Covalent interactions**: QuickFF requires ab initio equilibrium geometry and ab initio hessian at equilibrium to estimate the parameters in the covalent contributions of the force field energy expression. We used VASP[2,3] to obtain the equilibrium structure by geometry optimization, followed by performing a frequency calculation to obtain the hessian matrix (analytical second order derivatives of energy with respect to cartesian coordinates) and ab initio forces. With VASP, we worked with a single (111) unit cell, where the cutoff energy of 600 eV, Perdew Becke and Ernzerhof (PBE)[4][GGA exchange-correlation functional and k-points gamma mesh of $6 \times 3 \times 2$ was used to obtain the hessian matrix with considerable accuracy. D3 dispersion interactions together with the Becke-Johnson damping scheme (DFT-D3(BJ)) were included.[5,6] VASP-recommended projector augmented wave (PAW) pseudopotentials were considered for all elements in the MOF.[7] The electronic (ionic) convergence criteria is 10^{-8} (10^{-7}) eV. The output file from frequency calculation "vasprun.xml" contains all the structural information along with the hessian matrix which will be one of the inputs to QuickFF software.

2. The **electrostatic interactions** are modelled by coulomb interactions between Gaussian charge distributions. The atomic charges q_i are obtained from Minimal Basis Iterative Stockholder (MBIS) partitioning scheme.[8]

$$V_{ei} = \frac{1}{2} \sum_{i,j=1,i\neq j} \frac{q_i q_j}{4\pi\epsilon_0 r_{ij}} \text{erf}\left(\frac{r_{ij}}{d_{ij}}\right) \tag{2}$$

MBIS scheme is implemented in a package called DensPart[9] that is especially used for periodic structures to get the MBIS charges. DensPart works with GPAW[10–12] output

files. We performed a quick single point calculation with GPAW on the equilibrium structure obtained in VASP and "output.gpw" is obtained. This is then used with scripts given in DensPart package to convert GPAW files to DensPart readable "density.npz" file. The output "results.npz" file from DensPart is then used with QuickFF to generate the FF with inclusion of electrostatic interactions.

3. **Van der waals interactions** are described by MM3 model, parameters are taken from Allinger et al.[13] and added manually into the Force field file later.

$$V_{vdw} = \epsilon_{ij}\left[1.84.10^5 \exp\left(-12\frac{r}{\sigma_{ij}}\right) - 2.25\left(\frac{\sigma_{ij}}{r}\right)^6\right] \tag{3}$$

The parameters σ_{ij} and ϵ_{ij} are the equilibrium distance and depth of the potential. These parameters are determined with mixing rules for interaction between atom i and j:

$$\sigma_{ij} = \sigma_i + \sigma_j$$
$$\epsilon_{ij} = \sqrt{\epsilon_i \epsilon_j}$$

Step 2: Generation of Force field using QuickFF program

Apart from the hessian matrix in "vasprun.xml" file, atomic charges from DensPart "results.npz" file and van der waals parameters, another file with the system information MolMod.chk file is obtained from "vasprun.xml" by providing atom rules in ATSELECT language (`https://molmod.github.io/yaff/ug_atselect.html`). With all the needed files, an initial FF can be generated by following the procedure in QuickFF documentation. Please note, initially the default settings for the QuickFF were used for deriving the force field.

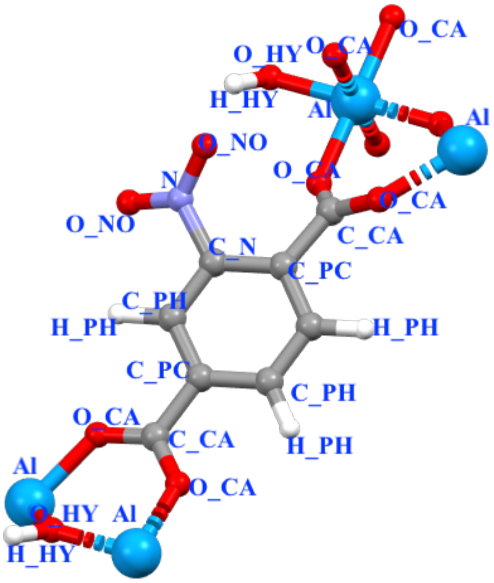

Figure S1: Figure showing labelled atom types in the asymmetric unit of NO_2-MIL-53 used for FF generation, FF validation and MD simulations. Elements notation from color are: blue: Aluminum, gray: Carbon, red: Oxygen, white: Hydrogen, lavender: Nitrogen.

Step 3: Rigid rotational scan with Ab Initio (AI) and generated FF

In this work, we are specifically interested in rotational freedom of linkers i.e., change in torsional value of specific dihedrals O_CA C_CA C_PC C_N and O_CA C_CA C_PC C_PH that changes with the rotation of terephthalate linker with respect to plane containing Al-OH linkages or the (011) plane. To be able to simulate the rotational dynamics accurately, torsional terms for these dihedrals in the previously generated FF in Step2 are replaced, and new torsional terms are added. The new terms are obtained by performing a rigid rotational energy scan of structures at different configurations ranging from −180° to 180° of OCCC dihedral angle both with AI and FF (containing all except the torsional OCCC term). The difference in the energies (E_{OCCC}) between AI and FF (containing all except the torsional OCCC term) rotational scans is then fitted to a cosine functional form shown in equation 5

$$E_{OCCC} = E_{AI} - E_{FF} \tag{4}$$

$$E_{OCCC} = \sum_{i=1}^{N_i} \frac{1}{2} A_i (1 - cos(M_i(\phi_i - \phi_{0,i}))) \tag{5}$$

Parameters A_i is obtained by fitting the difference in energies between AI and FF rotational

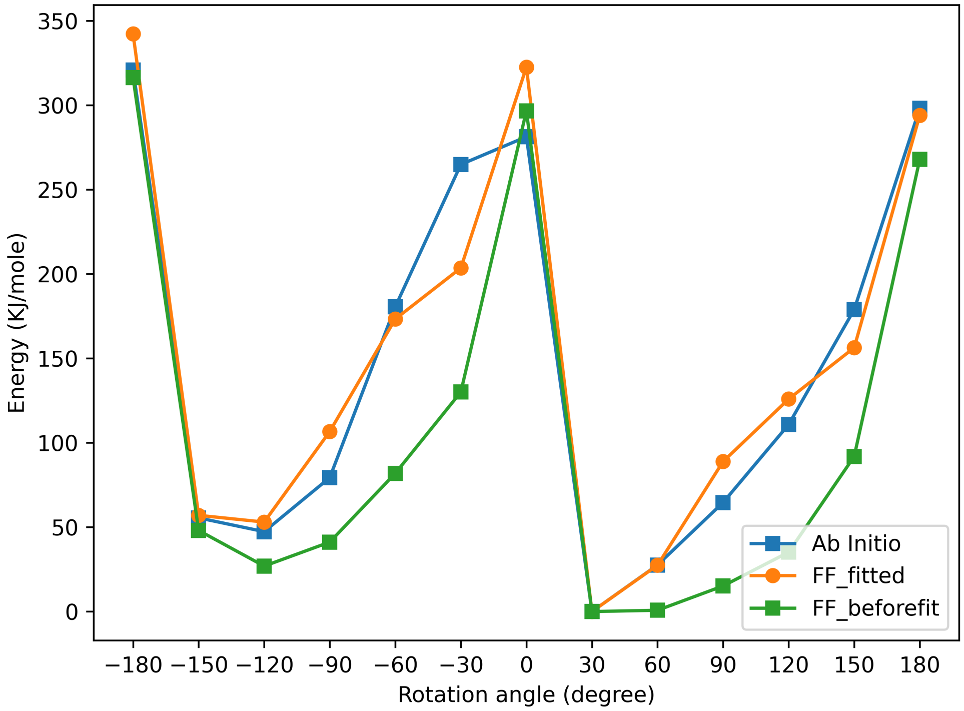

Figure S2: Figure showing rotational scan energies with Ab Initio, FF before fitting and FF after fitting.

scans into this functional form and $M_i = 2, 4, 6....$ The force constant A_i is estimated by minimizing the cost function method implemented in QuickFF called “boxqp” function (`http://molmod.github.io/QuickFF/_modules/quickff/tools.html#boxqp`). Figure S2 shows the energies of rotational scan with Ab Initio, Force field before fitting for the OCCC dihedral term and final FF energies after fitting for the OCCC dihedral term.

Note: All the python scripts and input/output files for each step in the FF development are made publicly available on the data repository.

Computational Methods

Geometry Optimization and Frequency Calculations

One of the force field validation methods was to compare the geometry optimized structure with developed FF to the experimental structure, The geometry optimization for this was done with YAFF[14] (`https://github.com/molmod/molmod`; `https://molmod.github.io/yaff/index.html`) for $2 \times 1 \times 1$ supercell. The electrostatic interactions were calculated using an Ewald summation with a real-space cutoff of 15 Å, a splitting parameter α of 0.213/Å, and a reciprocal space cutoff of 0.32/Å. Also, the van der Waals interactions were calculated with a smooth cutoff of 15 Å. The structure was optimized with convergence criteria set to 10^{-8} a.u. for the RMS on the cartesian gradient, 10^{-6} a.u. for the RMS of difference in the cartesian coordinates, 10^{-8} a.u. for the RMS on the gradient on the cell parameters and 10^{-6} a.u. for the RMS of the difference in the cell parameters. After the optimization, normal mode frequencies with FF were computed using TAMkin.[15]

Molecular Dynamics

In this work, we did not perform any AIMD simulations of $2 \times 1 \times 1$ supercell mentioned in the main paper. The data from AIMD simulations which were obtained in the previous work[16] was used to obtain the free energy surface (FES) plots and compare with the FF generated FES.

Classical MD simulations for $2 \times 1 \times 1$, $4 \times 2 \times 2$ and $6 \times 2 \times 2$ supercells were all performed with LAMMPS software (`https://www.lammps.org/index.html#gsc.tab=0`).[17–19] Starting from the geometry optimized DFT structure, MD simulations were performed in the (N,V,T) ensemble with fixed size and shape of the unit cell. A time step of 0.5 fs was used in the MD runs and temperature was controlled by a canonical sampling through Nose-Hoover thermostat. The equilibration period for FF validation calculations with $2 \times 1 \times 1$ supercell was 10 ps and a production period of 1 ns for all temperatures (300 K and 450 K). For $4 \times 2 \times 2$

and $6 \times 2 \times 2$ supercells, the equilibration period is 100 ps and a production period of 70 ns.

Force Field (FF) Validation

Geometry Optimization

The obtained FF should accurately predict the geometry of the Nitro functionalized MIL-53 structure. For that, we compare the FF simulated cell parameters with the experimental values of large pore structure of NO_2-MIL-53 (see Table S1). The FF simulated structure optimizes to an orthorhombic shape, in agreement with the experimental structure. The deviation between experimental and simulated lattice parameters a, b and c is 0.2%, 6.5% and 3.1% respectively; where deviation on b is the highest among other directions. This is due to the inherent flexibility of the MIL-53 framework in the plane of the wine-rack motif i.e, *bc* plane perpendicular to the inorganic chain. However, the overall predicted structure from the geometry optimization with FF is similar to the experimental structure.

Table S1: Comparison of Lattice Parameters of NO_2-MIL-53(Al) structure.

Lattice Parameters	Experimental[20]	FF simulated
a [Å]	13.298	13.331
b [Å]	13.320	14.188
c [Å]	16.382	15.877
[deg]	90	90
[deg]	90	90
[deg]	90	89.98

Comparison of Force Field (FF) Frequencies with DFT Frequencies

We perform the next validation of the FF by comparing the DFT calculated normal-mode frequencies to that of the FF frequencies as shown in Figure S3. They show a very good correlation at all ranges of frequencies. We further compute the Internal energy, the Helmholtz free energy, the entropy and the heat capacity of the NO_2-MIL-53 $2 \times 1 \times 1$ supercell, as a function of temperature in the quantum-harmonic approximation using the computed

normal mode frequencies from both DFT and FF.[1] (see Figure S4) All these thermodynamic properties are reproduced very well with FF frequencies when compared to the DFT computed properties. Validation of the FF with geometry optimization and frequency comparison shows the FF accurately reproduces the experimental geometries, unit cell dimensions and frequencies of normal modes.

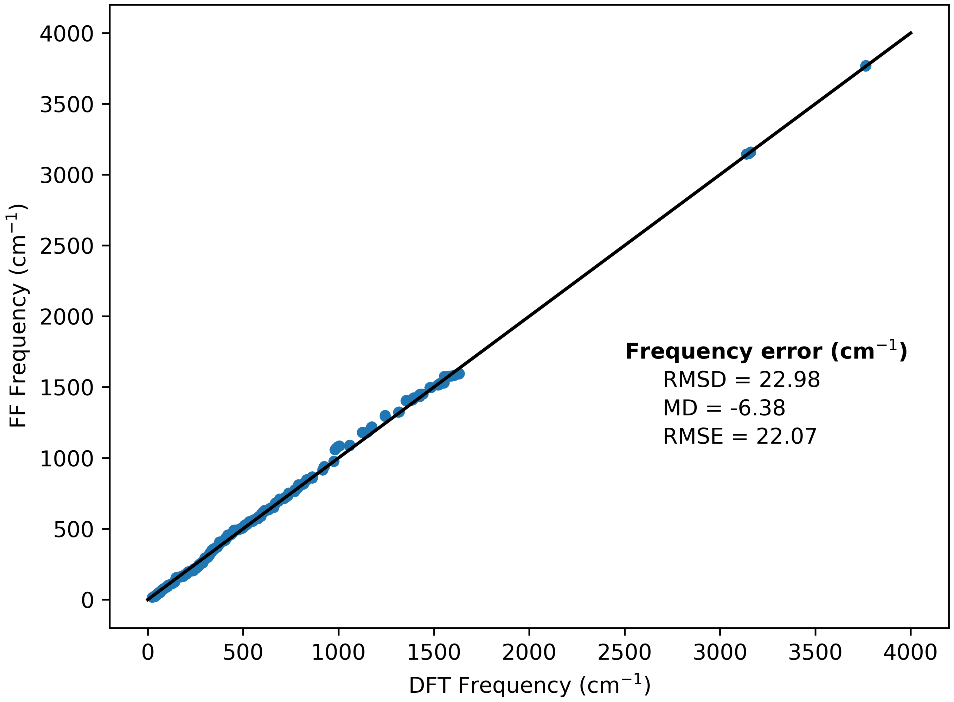

Figure S3: Comparison of DFT and FF calculated frequencies. Three measures of error between DFT and force field frequencies are indicated: RMSD the root-mean-square deviation as a measure of total error, MD the mean deviation as a measure of the systematic error, RMSE the root-mean-square error as a measure of the non-systematic error.

$$E(T) = \sum_{i=1}^{N_\omega} \left(\frac{\hbar\omega_i}{2} + \frac{\hbar\omega_i}{e^{\beta\hbar\omega_i} - 1} \right) \tag{6}$$

$$F(T) = \sum_{i=1}^{N_\omega} \left(\frac{\hbar\omega_i}{2} + k_\mathrm{B}T\ln[1 - e^{-\beta\hbar\omega_i}] \right) \tag{7}$$

$$C_v(T) = k_\mathrm{B} \sum_{i=1}^{N_\omega} \left(\frac{\hbar\omega_i}{k_\mathrm{B}T} \right)^2 \frac{e^{\beta\hbar\omega_i}}{(e^{\beta\hbar\omega_i} - 1)^2} \tag{8}$$

$$S(T) = k_{\mathrm{B}} \sum_{i=1}^{N_\omega} \left(\frac{\beta\hbar\omega_i}{e^{\beta\hbar\omega_i} - 1} - ln[1 - e^{-\beta\hbar\omega_i}] \right) \tag{9}$$

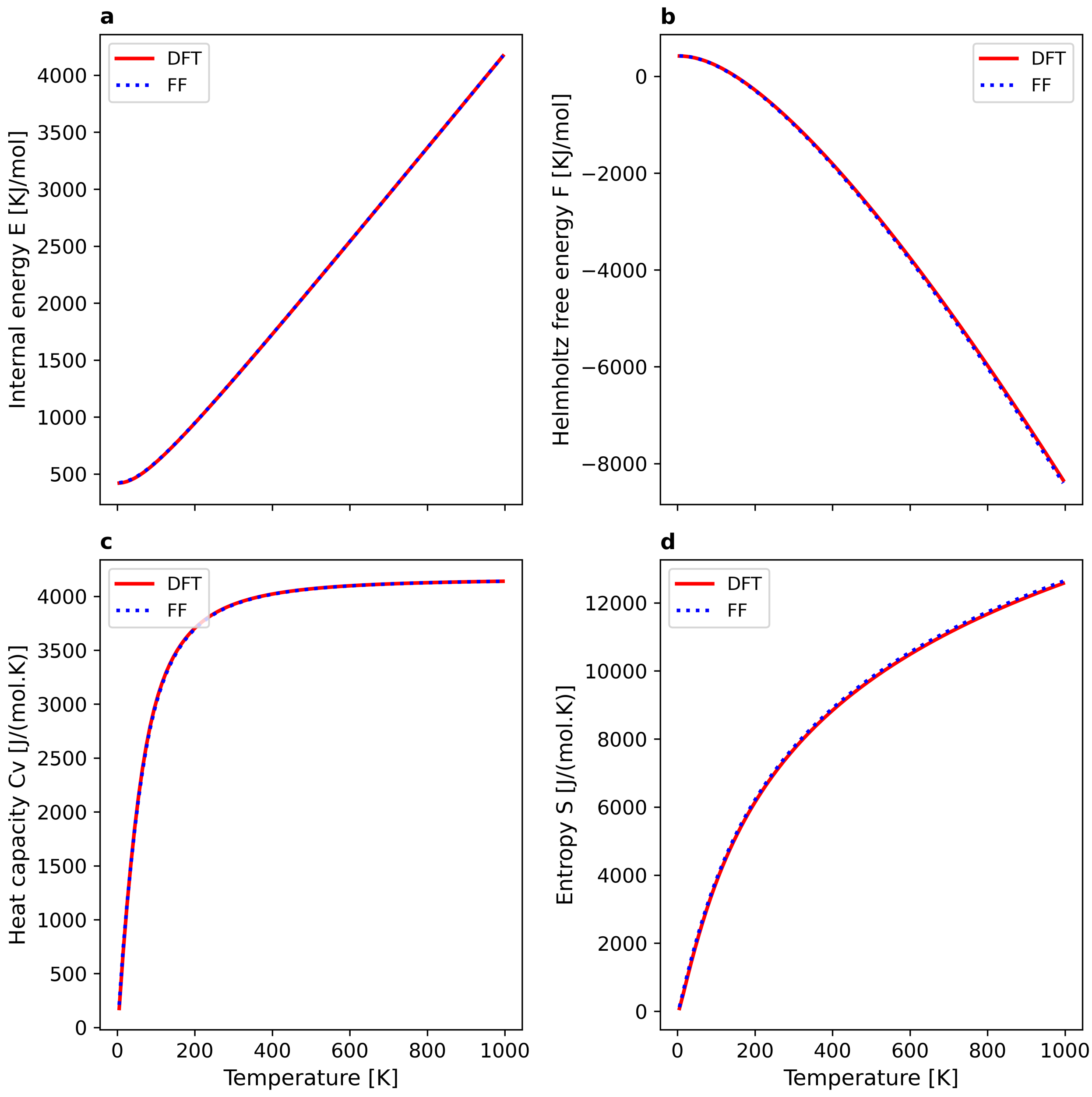

Figure S4: a) Internal energy E, b) Helmholtz free energy F, c) heat capacity C_v and d) entropy S of nitro functionalized MIL53 as a function of temperature in the harmonic oscillator approximation using the computed DFT (red line) and force field frequencies (blue dotted line).

Comparison of Molecular Dynamics based on Ab initio and FF Calculations

The goal of this work is to use the obtained FF for studying the rotational linker dynamics in NO_2-MIL-53 for bigger supercells. Hence, to validate the FF, we did MD simulations with the FF for a $2 \times 1 \times 1$ supercell for a simulation time of 1 ns and compared these results with AIMD results for the same supercell for a simulation time of 40 ps. These AIMD results were already published in one of the previous works on linker dynamics in NO_2-MIL-53. From the MD simulations with AIMD and FF, the rotational angle of the Nitro terephthalate linkers is obtained using a python code used in the previous work.[16] The $2 \times 1 \times 1$ supercell has four sets of chains in the unit cell where each chain has two rotating linkers that are direct neighbors along the pore direction.(see Figure S5) Hence a total of eight linkers are present in the supercell.

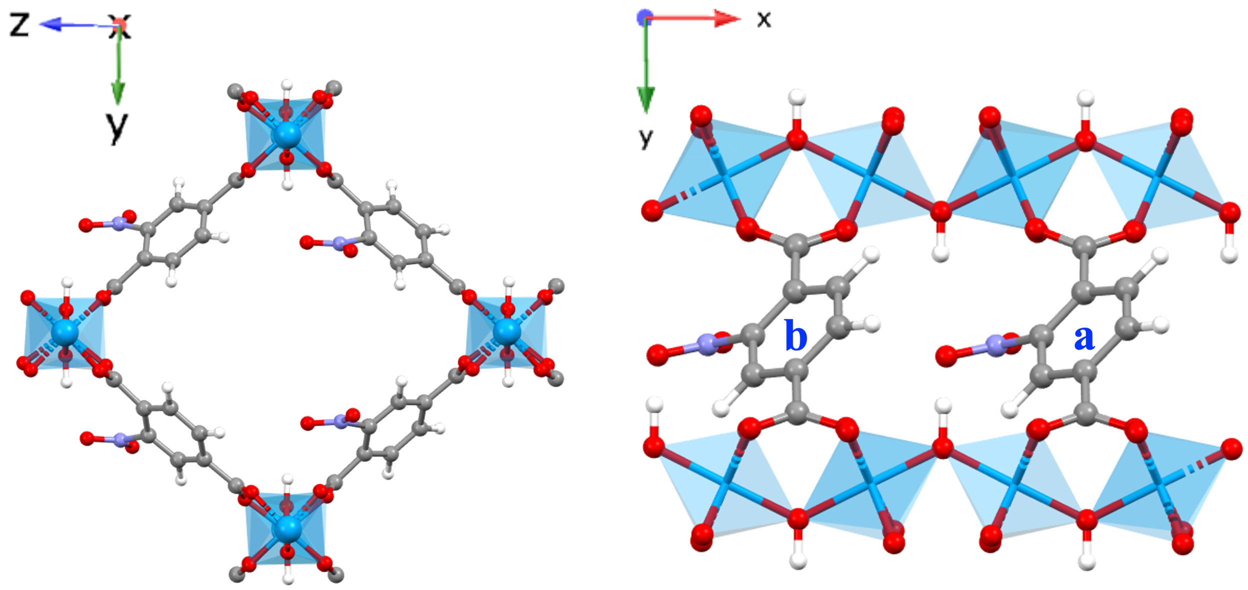

Figure S5: Figure showing the pore of the $2 \times 1 \times 1$ supercell (left) and (right) two rotating linkers (a,b) along the pore direction (x) in a single chain in the $2 \times 1 \times 1$ supercell.

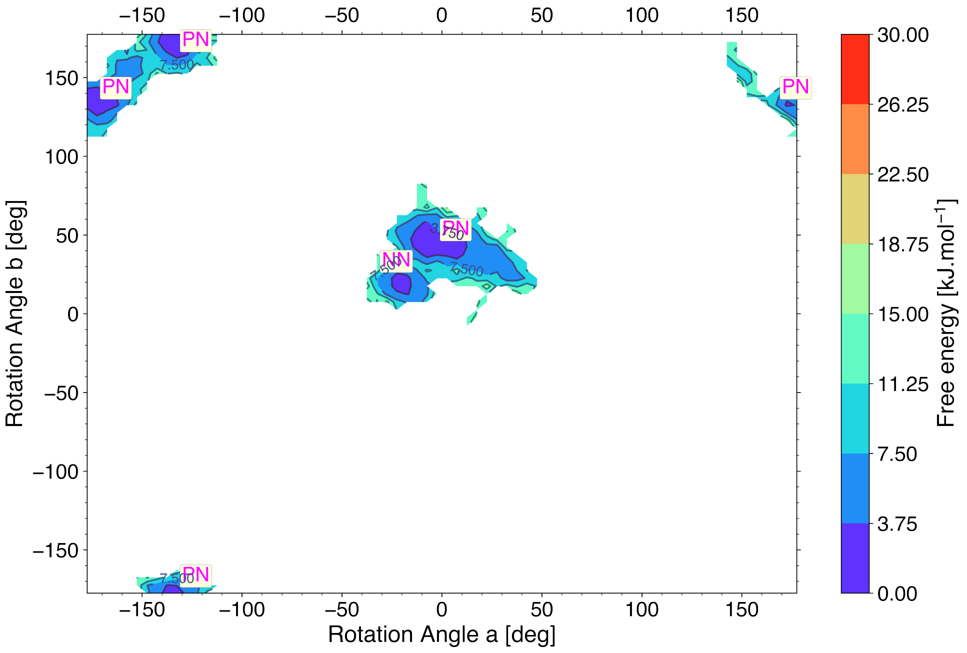

Figure S6: Free energy surface (FES) obtained with AIMD from rotational angle data of neighboring linkers in a $2 \times 1 \times 1$ supercell at 300 K.

AIMD

In AIMD simulations, at low temperature (300 K, Figure S6), the width of the FES minima is around 10°. With an increase in temperature (see Figure S7), the minima in the FES become wider (20° to 30°), due to the increased librations of the linkers at high temperatures. At 300 K, the lowest energy minima are majorly seen at orientations in *PN* regions where at least one linker is planar, and the other linker is oriented at 50° or ±135°. Region *NN* with acute angles of (−25°, 25°) is also observed in scarce instances at 300 K. With an increase in temperature to 450 K and 700 K region *NN* becomes more prominent, the planar orientation of linkers gradually disappears, and we see oblique angles for the linkers i.e., centered around (−140°, 25°) or (140°, 150°).

FF

The FES from 1 ns FF simulations at two temperatures 300 K and 450 K are shown in Figure S8 and S9. Considering first the FES at 300 K we observe that the minima are wider and

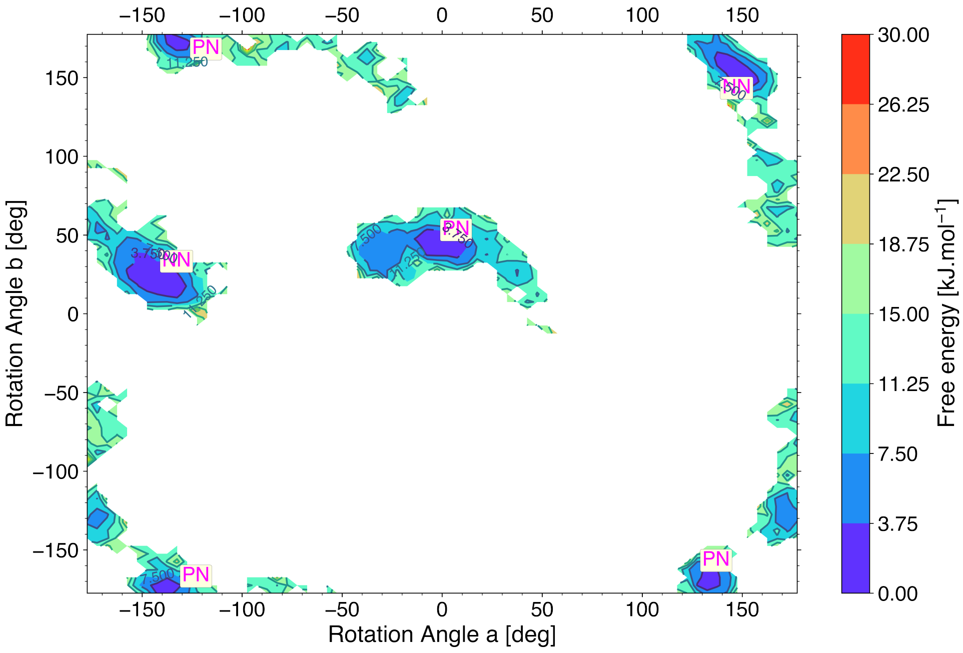

(a)

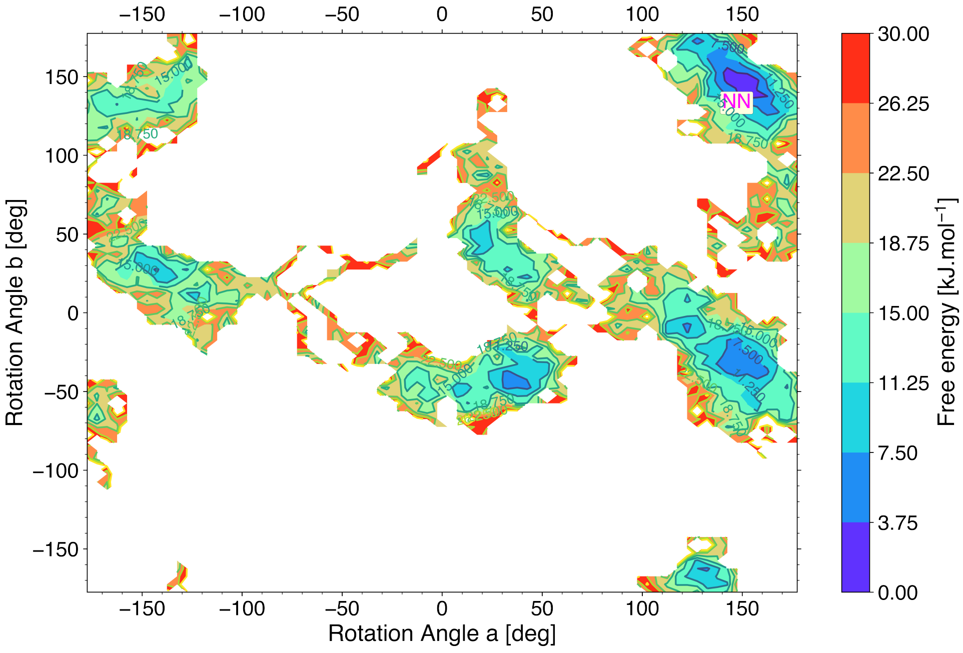

(b)

Figure S7: Free energy surface (FES) plots obtained from rotational angle of neighboring linkers in a $2 \times 1 \times 1$ supercell with AIMD at temperatures a) 450 K and b) 700 K.

mostly seen at orientations in region PN where linker a is planar (0° or ±180°), and the other linker b is oriented between 80° and 130° or ±50° to ±100°. Some sampling of region

NN is also observed with an acute angle for both linkers a, b centered around (30°, 30°). With an increase in temperature, region NN becomes prominent and centered around (30°, 30°) and (−150°, −150°). Note here, region NN may not be a minima, but it is a transition state between the two adjacent PN minima regions. This is confirmed later in the longer MD simulations of a 4 × 2 × 2 supercell in Figure 4 in the main paper.

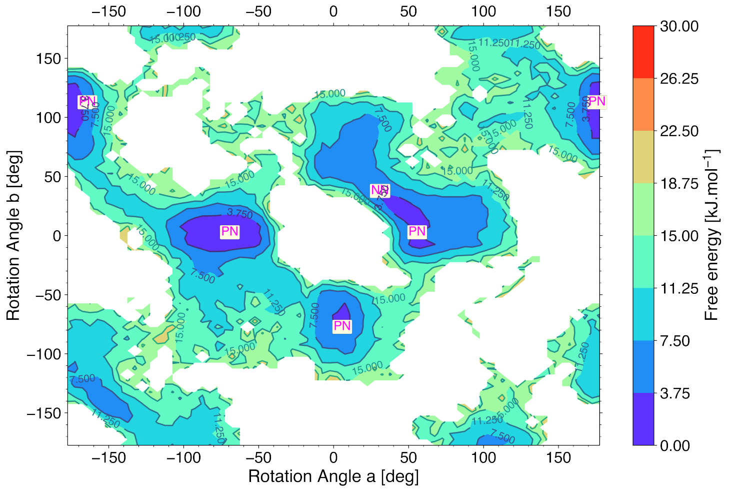

Figure S8: Free energy surface (FES) obtained with FF from rotational angle data of neighboring linkers in a 2 × 1 × 1 supercell at temperature 300 K.

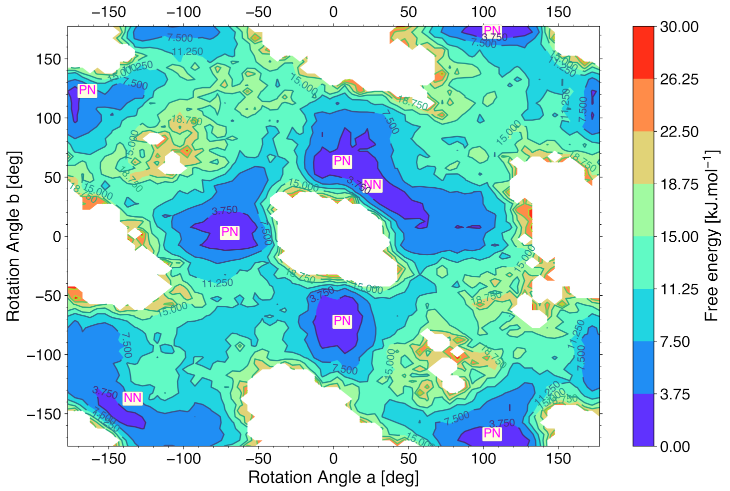

Figure S9: Free energy surface (FES) plots obtained from rotational angle of neighboring linkers in a 2 × 1 × 1 supercell with FF at 450 K.

$4 \times 2 \times 2$ Supercell

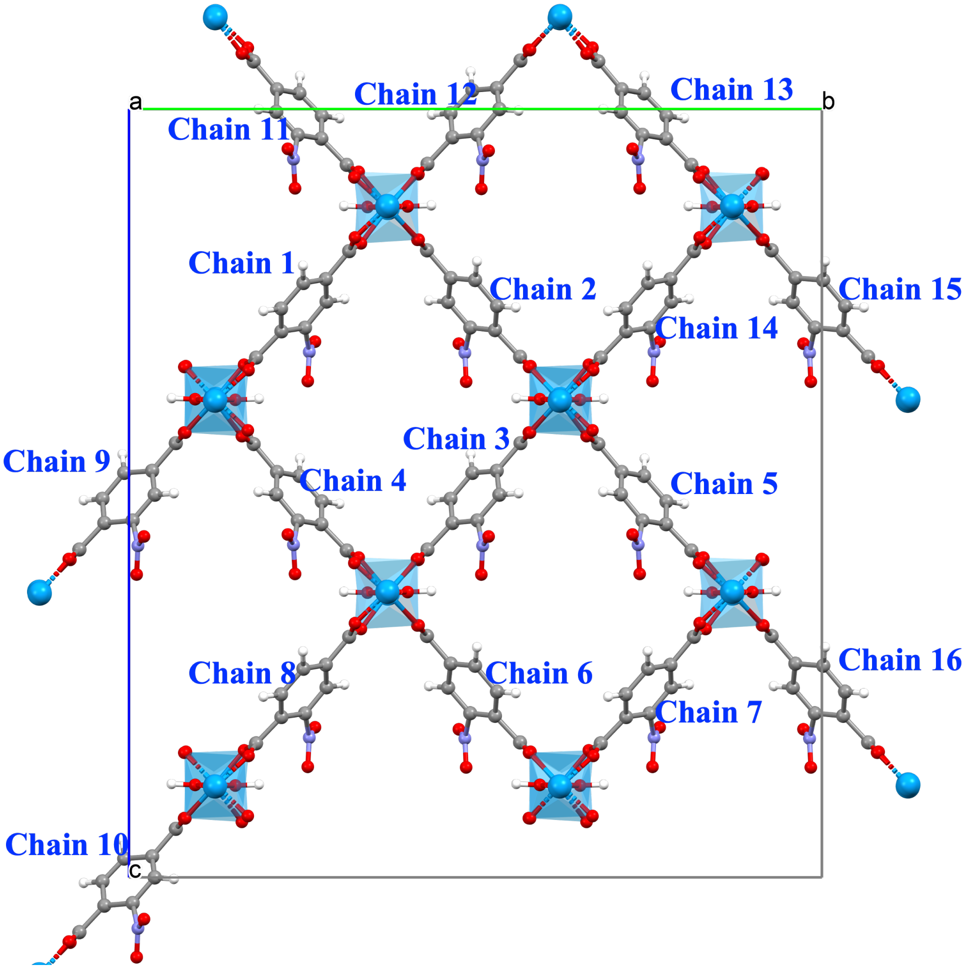

Figure S10: Figure showing $4 \times 2 \times 2$ supercell with all 16 chains in the supercell.

Interactions with Neighboring Linkers along Perpendicular to Pore Direction

In the main paper, we elaborated on neighboring interactions between linkers along the pore direction (x). Here, we present and discuss the time trajectories for some of the linkers and its perpendicular neighbors along y or z direction. The notation for the different sets of chains shown in Figure S10 is followed for the time traces below.

Structurally, linkers from different chains directly stack over each other when there is a

hydroxyl group between the chains. For example a hydroxyl group is present between chains (1,4), (2,3), (4,8), (5,7) etc.(shown in Figure S12a) The other kind of chain pairs are those where the hydroxyl group is not present between them and chains do not directly stack over each other but are alternately stacked. Some examples of these pairs include (3,4), (6,7), (4,9), (2,14) etc.(shown in Figure S12b) The steric interactions should be higher for the chain pairs with hydroxyl group between them because of the direct stacking along y and z directions. Hence, we look closely into those chain pairs with hydroxyl group between them for linker interactions.

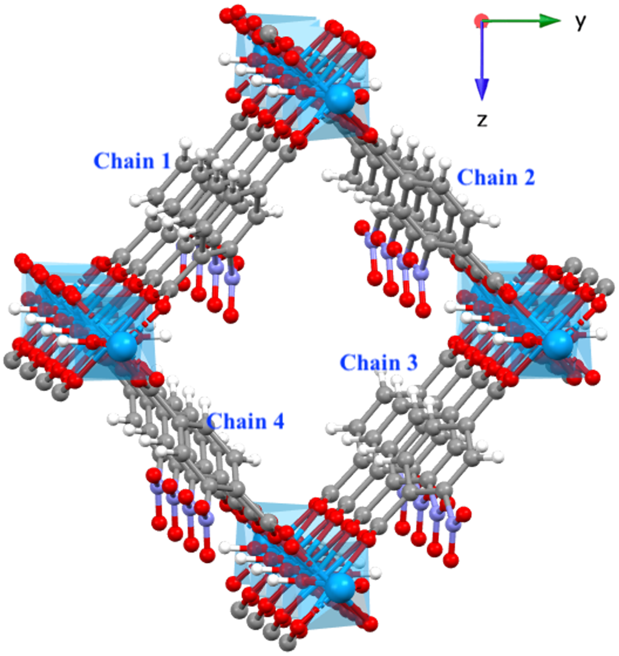

Figure S11: Figure showing chains 1, 2, 3, and 4 of the $4 \times 2 \times 2$ supercell in S10 along ‘x’ direction.

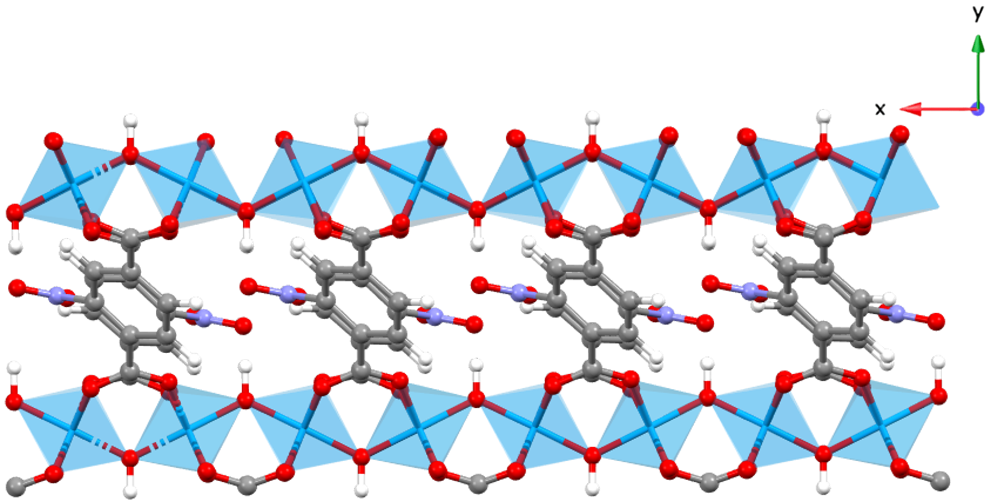

(a) Chains 1 and 4 with hydroxyl group in between showing directly stacked over each other when viewed along ‘z’ direction.

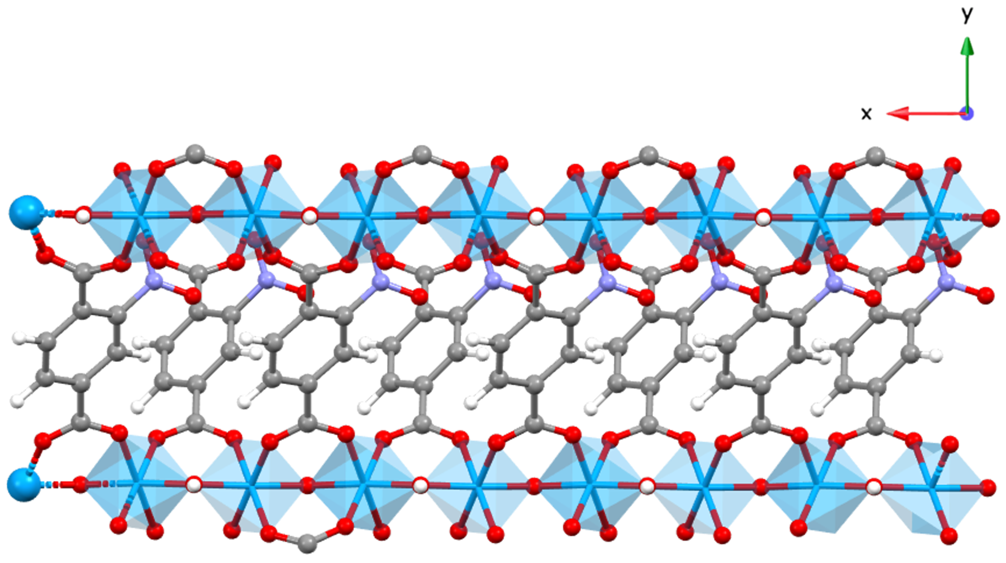

(b) Chains 3 and 4 without hydroxyl group in between showing alternately stacked when viewed along ‘z’ direction.

Figure S12: Chains (a) with hydroxyl group in between, (b) without hydroxyl group in between.

Qualitatively we look for changes in the perpendicular neighbor linker pairs, when there is a change in conformation from 0° to ±180° or vice versa, or change in width of the librations in either of the linkers. In Figure S13a and S13b, linkers librate around (±180°, 0°) and (−90°, 90°) i.e., (P,P) and (N,N) configurations respectively. In Figure S14a and S14b libration of linkers in this case is around (90°, 0°) and (0°, 90°) i.e., (N, P) and vice versa. Similarly, for linkers in Figure S15 conformations are (N, P) and (P, P). So, for all different combinations of linker conformations P and N where P = librations around 0° or ±180° and N = librations around, ±90° we did not observe a distinct change in the perpendicular linker due to change in the other linker conformation by 180°flips or changed width of librations.

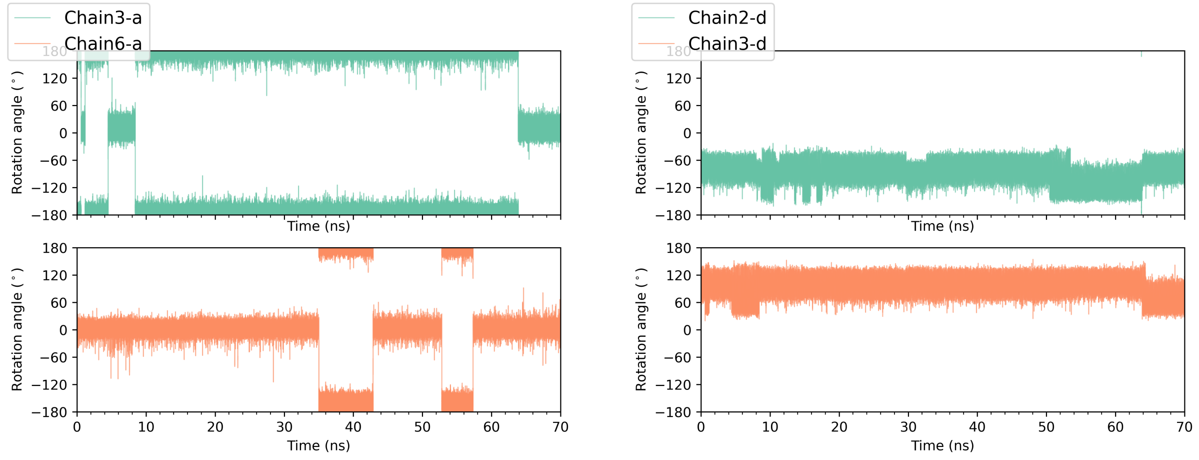

(a) **(b)**

Figure S13: MD time trajectories of one of the linkers in chains (3,6) and (2,3).

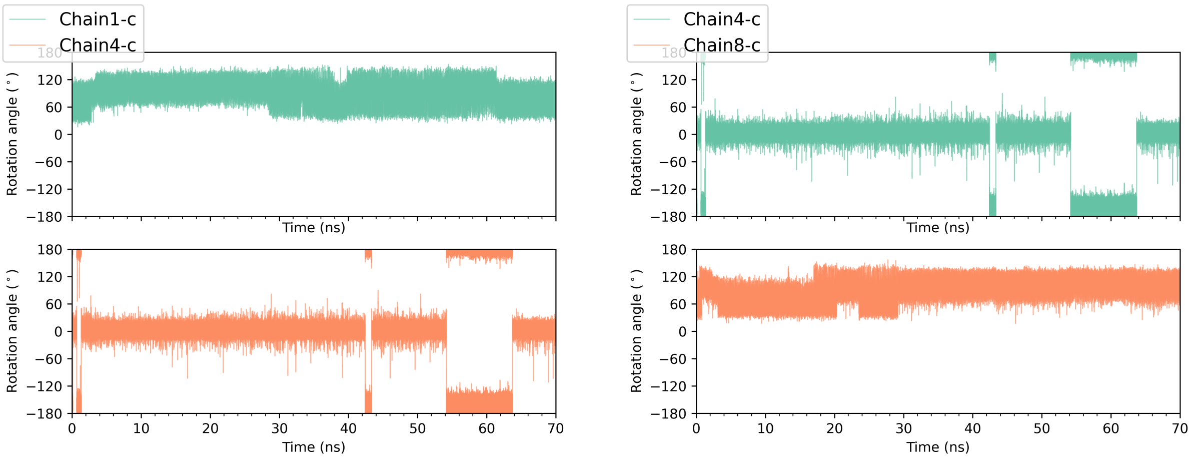

(a) **(b)**

Figure S14: MD time trajectories of one of the linkers in chains (1,4) and (4,8).

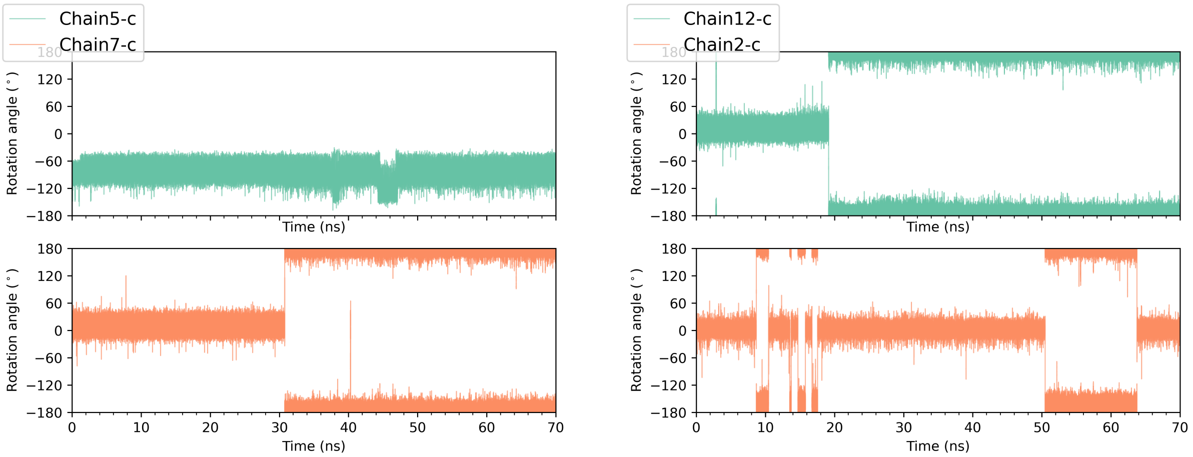

(a) **(b)**

Figure S15: MD time trajectories of one of the linkers in chains (5,7) and (12,2).

Energetics of 180° Rotations in Planar Linkers

To obtain the energy barrier for the 180° flips of a single planar linker, we zoom into one of the rotational flips and show an illustrative path followed on the FES during the flips. Figure S16a shows closely the rotational angle change of linker *d* between 33.17 ns and 33.21 ns for the same chain as in Figure 7a in the main paper. The rotational flip occurs between the indicated dotted lines over a time of, ∼0.004 ns which is 4 ps. The conformational changes occurring during the rotational flip in linker *d* and its direct neighbors linkers *a* and *c* are shown as the rotational path on the FES with direct neighbors as collective variables in Figure S16b. Since linker *d* has two direct neighbors *a* and *c*, the transition path considering both the neighbors is shown during the flip from *start* to *end* for *ad* and *cd*. From the starting conformations in the minima at *start ad* to the final 180° apart conformation for linker *d* at *end ad*, the transition requires for linkers *ad* to overcome a transition state energy of around ∼ 24 kJ/mol. A similar transition path is also observed for linkers *cd*, with an energy barrier of ∼ 24 kJ/mol. The free energy for these transition states is higher than the rotational energy barrier for a 180° rotation of nitro-substituted terephthalic acid molecule (17.1 kJ/mol) where neighboring interactions are not present but lower than experimentally obtained activation energy values for the NO_2-MIL-53 MOF (32.3 ± 1.3 kJ/mol)[16].

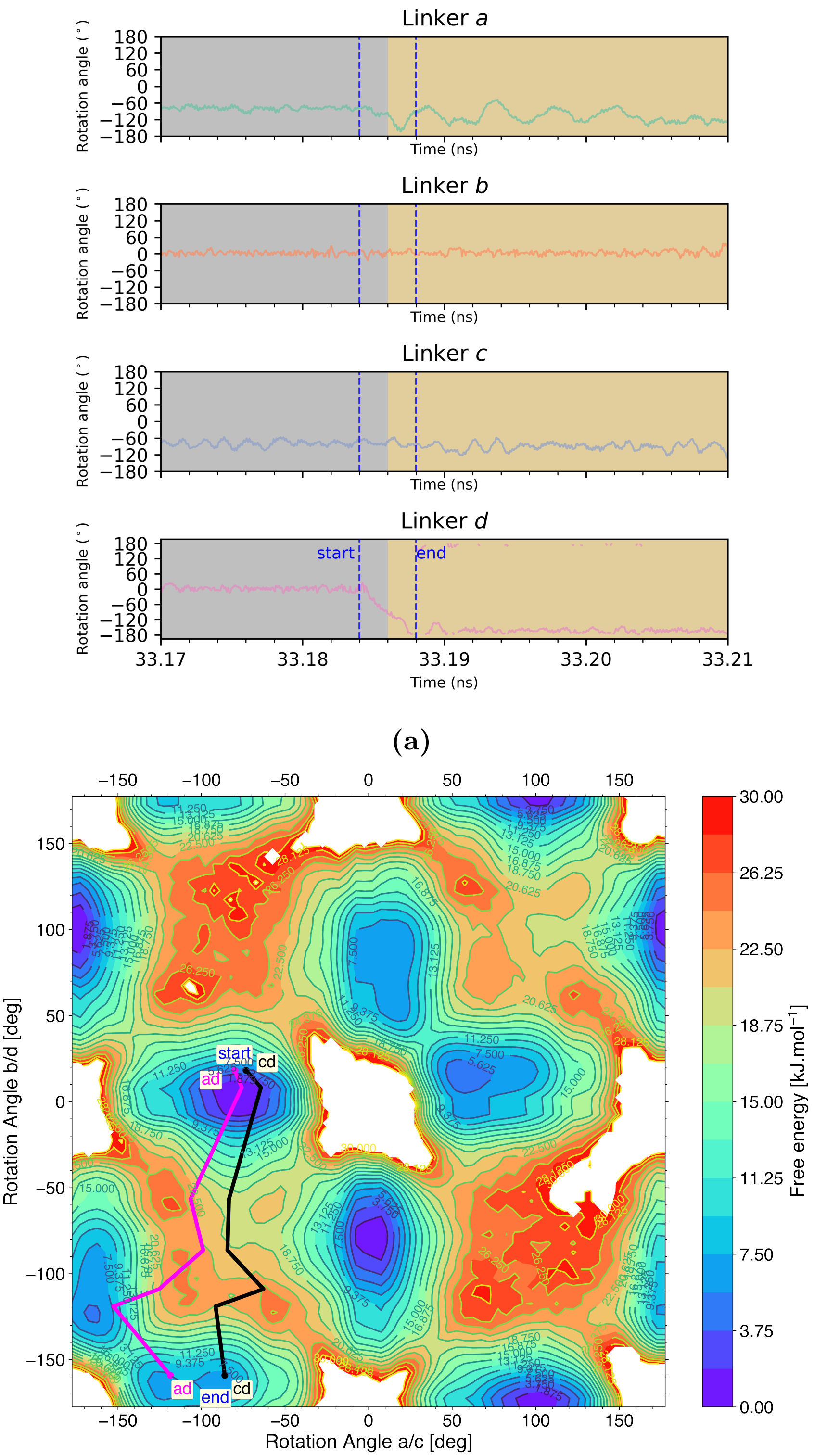

(a)

(b)

Figure S16: a) Zoomed in MD trajectory at t1 and between regions ΔT01 and ΔT12 (from Figure 7a in the main paper). Dotted blue lines indicate the start and end of the rotational angle change of the planar linker *d*. b) FES of "1, 2" type neighbors showing an illustrative path of the 180° rotational angle change of planar linker *d* and the "*ad*" and "*cd*" pathway.

"1, 3" Type Interactions

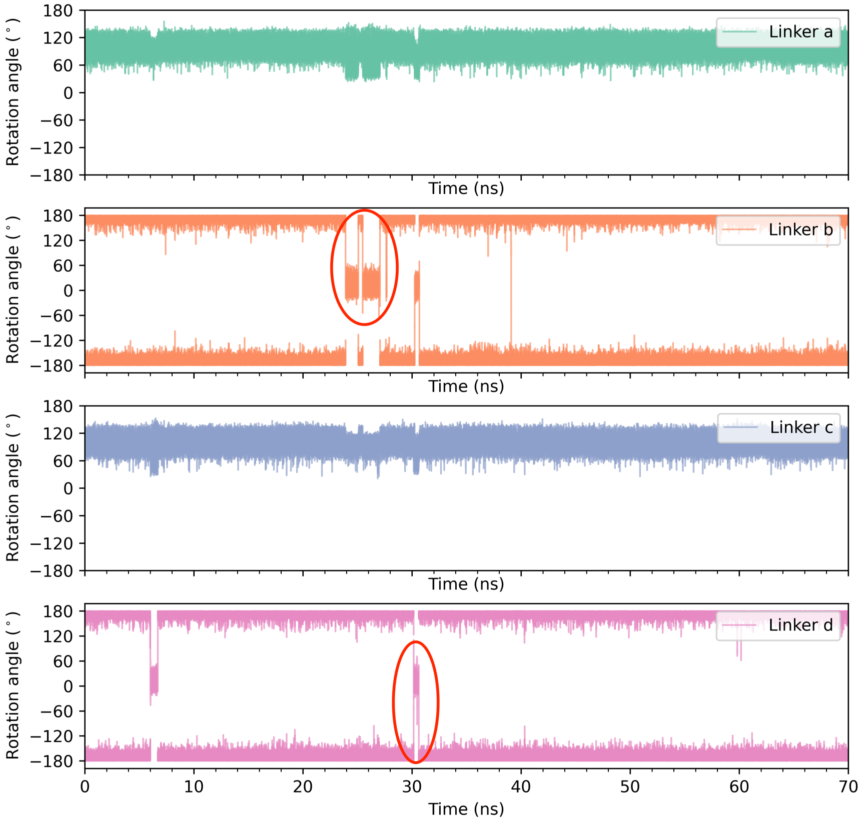

Figure S17: MD time trajectory of a chain in the $4 \times 2 \times 2$ supercell, highlighting 180° rotational flips for a short time of ∼1.5 ns.

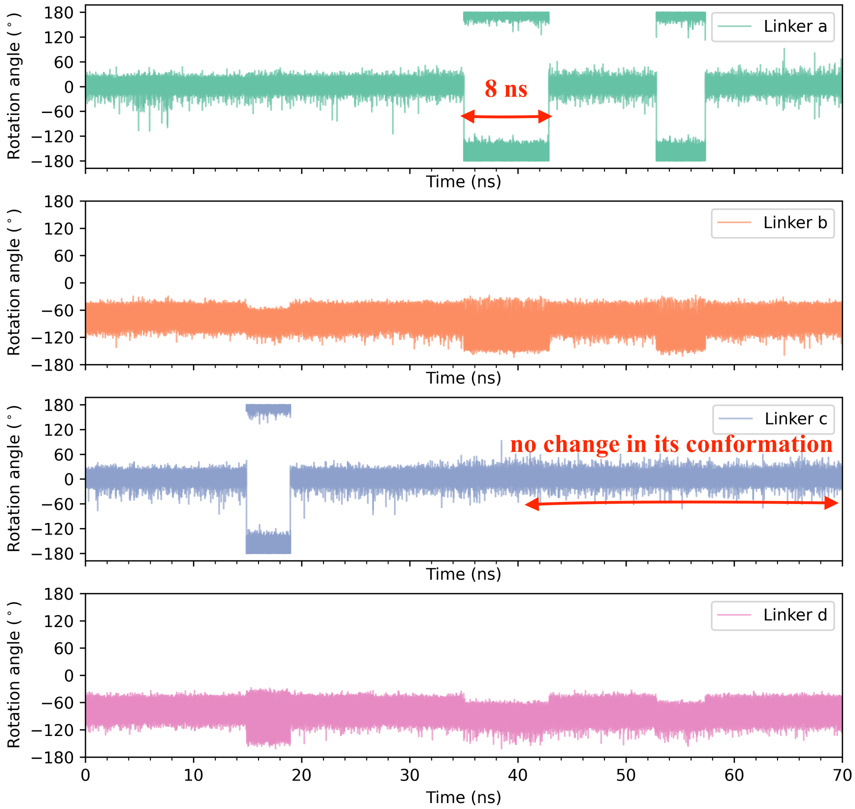

Figure S18: MD time trajectory of a chain in the $4 \times 2 \times 2$ supercell, highlighting 180° rotational flips for a time of $\sim$8 ns.

$6 \times 2 \times 2$ Supercell

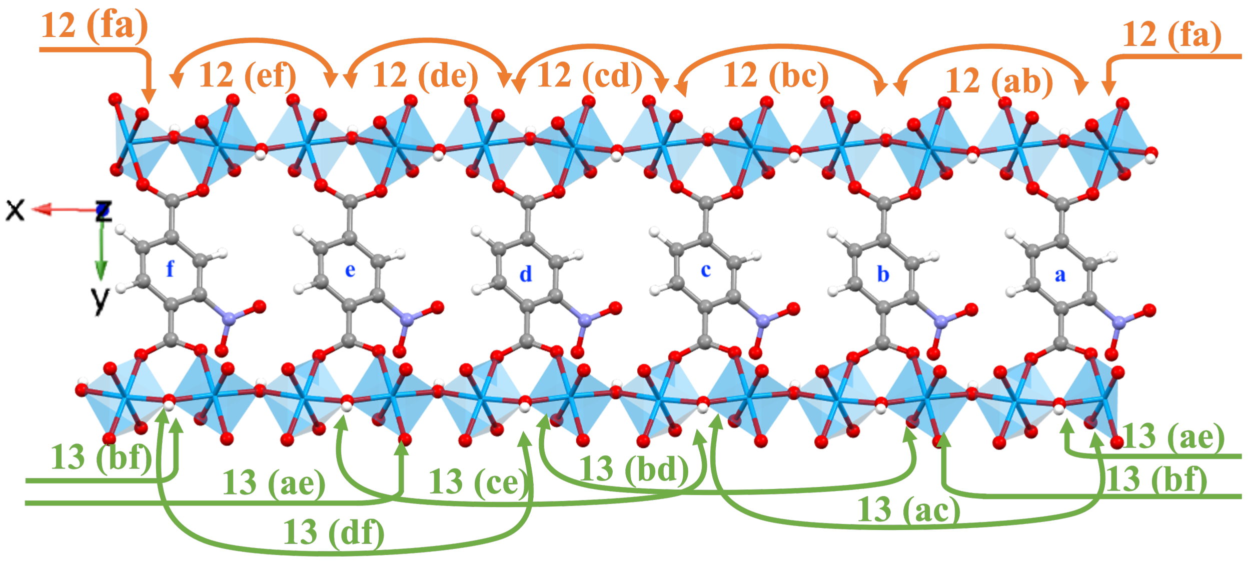

Figure S19: Figure showing the six rotating linkers (a, b, c, d, e, f) along the pore direction (x) and their "1, 2" type (orange: includes pairs ab, bc, cd, de, ef, fa), "1, 3" type (green: includes pairs ac, ae, bd, bf, ce, df) type neighbors for a single chain in the $6 \times 2 \times 2$ supercell.

Note: For the FES plots of "1, 3" type neighbors, linker pairs *ce* and *df* were not included to maintain the consistency of linkers selection on x axes (i.e., *a, a, b, b*) and y axes (i.e., *c, e, d, f*) of the plot. The FES "1, 3" type neighbors plot remained constant with and without inclusion of *ce* and *df* pairs of linkers.

Figure S20 shows the FES of the $6 \times 2 \times 2$ supercell ranging from $-180°$ to $180°$ for the "1, 2" type neighbors as collective variables. In line with the FES of the $4 \times 2 \times 2$ supercell for "1, 2" type neighbors (Figure 4), we only observe region *PN*, and the locations of minima in the $6 \times 2 \times 2$ supercell are at the same rotational angles as for the $4 \times 2 \times 2$ supercell. Figure S21 shows FES for $6 \times 2 \times 2$ supercell with alternate or "1, 3" type neighbors as collective variables. The deepest minima ($PP(\uparrow\uparrow)$ and $NN(\uparrow\uparrow)$) in the FES plot of "1, 3" type neighbors for a $6 \times 2 \times 2$ supercell are located along the diagonal and corners of the FES plot and the less deep minima ($PP(\uparrow\downarrow)$) are located on the edges of the FES, similar to the $4 \times 2 \times 2$ supercell. This implies alternate neighbors or "1, 3" type linkers are librating at

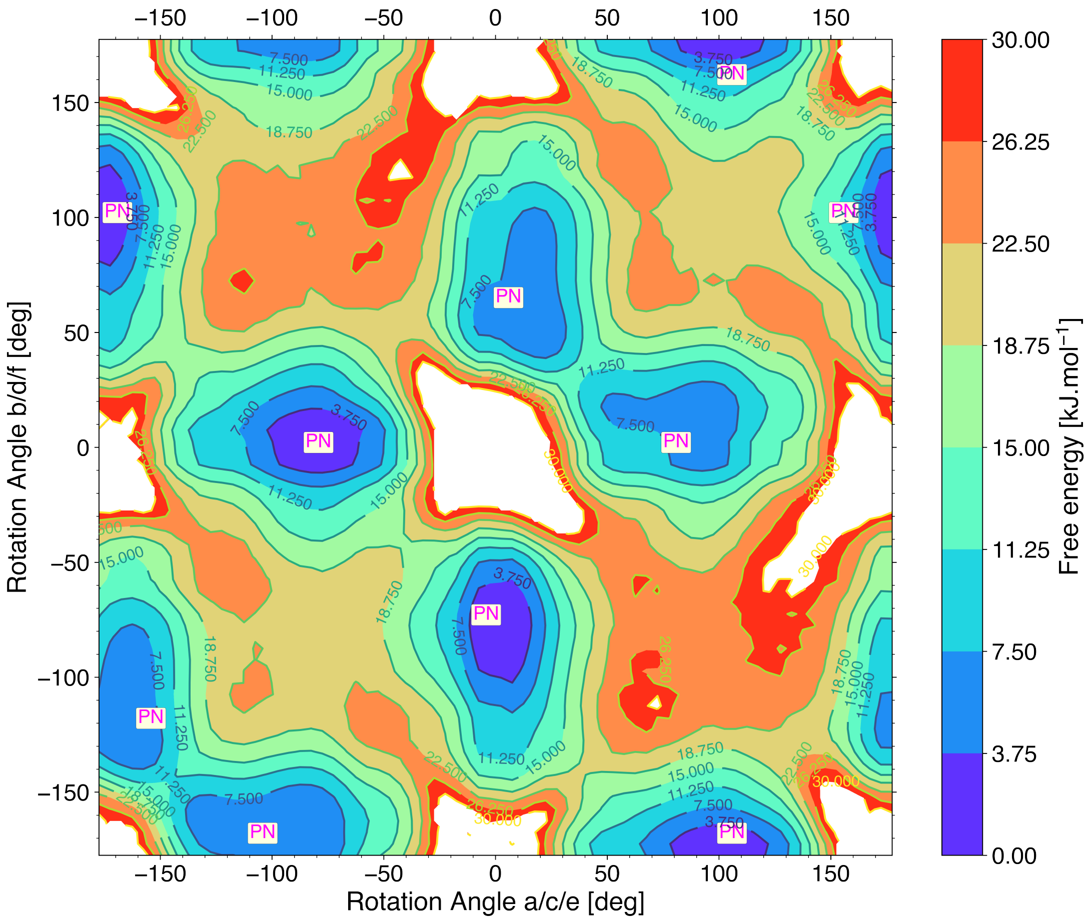

Figure S20: Free energy surface (FES) for a $6 \times 2 \times 2$ supercell, considering rotational angle of "1, 2" type neighbors as collective variables.

the same rotational angles compared to the $4 \times 2 \times 2$ supercell. The free energy surface plots of $6 \times 2 \times 2$ supercell are qualitatively and quantitatively identical to that of the $4 \times 2 \times 2$ supercell.

Based on the time trajectories of the different chains in the $6 \times 2 \times 2$ supercell, we show here representative examples and discuss trends that are generally occurring. For instance, in Figure S22 linkers *a, c, e* are librating between $b = 40°$ and $120°$ while linkers *b, d, f* are librating around $a = 0°$ or $\pm 180°$. Between 10 ns and 14 ns, linkers *b, d, f* are librating around 0° and are parallel(↑↑↑) aligned with each other. At ∼14 ns linker *b* rotates to ±180°, thus being antiparallel(↓↑↑) aligned with respect to conformations of linkers *d, f*. As a result, linker *c* librates over a large angle range ("1, 2" type interaction) as NO_2 groups from both

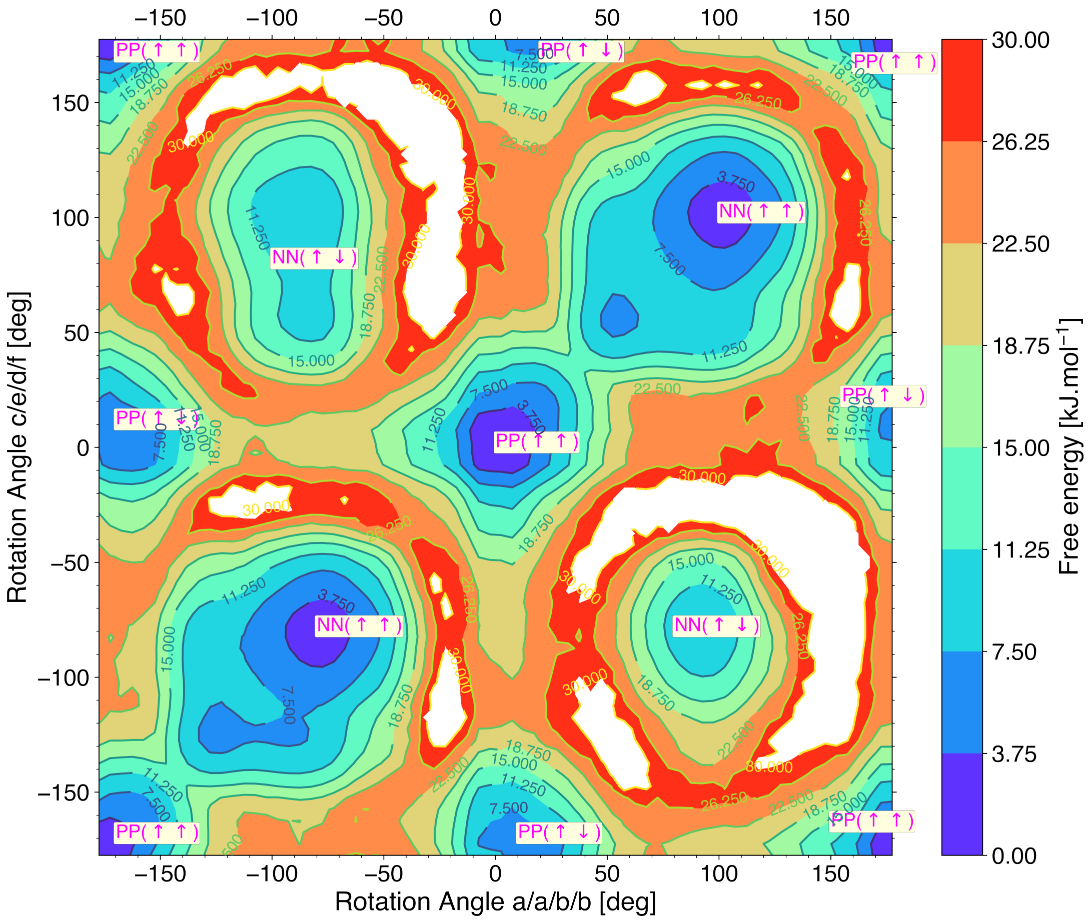

Figure S21: Free energy surface (FES) for a $6 \times 2 \times 2$ supercell, considering rotational angle of "1, 3" type neighbors as collective variables.

neighboring linkers b,d are pointing away from linker c, while linker a librates over a slightly narrower range due to the NO_2 groups from both neighboring linkers $b,$ d pointing towards it. The rotation of linker b to ±180° at 14 ns is followed by linker d to flip from 0° to ±180°("1, 3" type interaction) around 18 ns and becomes parallel aligned with linker b but antiparallel to linker f (↓↓↑). Due to the rotational change of linker d, wider librations in linker e and narrower librations in linker c are observed ("1, 2" type interaction). This is then followed by linker f to make the transition from 0° to ±180° ("1, 3" type interaction) around 24 ns. At this point all three linkers $b,$ $d,$ f are again parallel aligned (↓↓↓).

At ∼6 ns, we observe dynamics that are rarely observed compared to the above discussed "1, 3" type interactions. The linker d flips from 0° to ±180° conformation, thus becoming

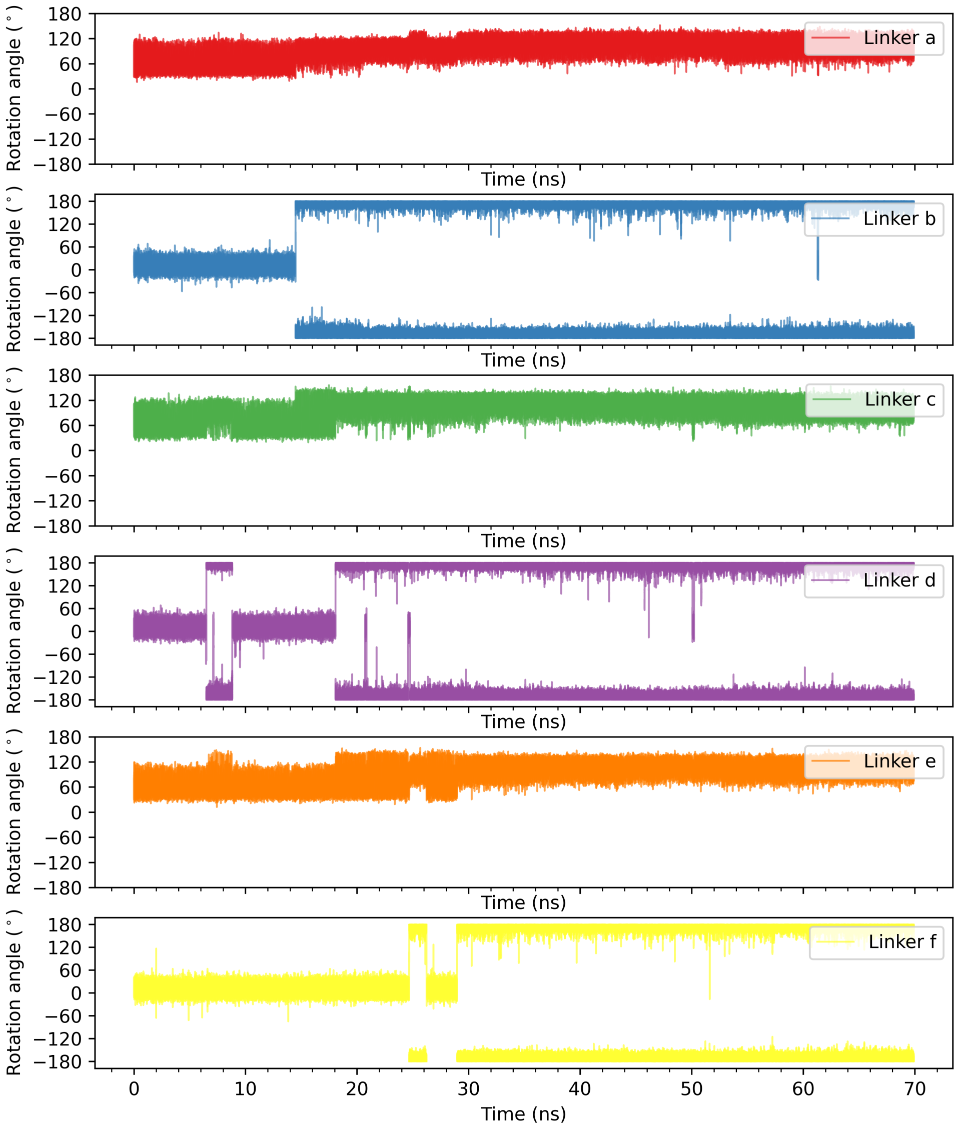

Figure S22: MD trajectory of the four linkers along the row for a specific chain in the $6 \times 2 \times 2$ supercell.

antiparallel (↑↓↑) aligned with linkers *b, f*. The linkers *b, f* continue librating at 0° and remain in this conformation, while linker *d* flips back to its original conformation after a few nanoseconds at 9.3 ns, restoring the original parallel alignment of the three linkers *b, d, f* (↑↑↑). A similar situation is observed for linker *f* from 26 ns to 29 ns. At 26 ns, linker *f* flips from ±180° to 0°, thus flips to an antiparallel (↓↓↑) aligned state with linkers *b, d*. At 29 ns, linker *f* rotates back to a parallel alignment with linkers *b, d* (↓↓↓) its "1,3" type neighbors.

The "1, 2" and "1, 3" type interactions in the $6 \times 2 \times 2$ supercell are similar to the

interactions observed for a $4 \times 2 \times 2$ supercell. Additionally, the time between rotational flips of "1, 3" type planar neighbors in the $6 \times 2 \times 2$ supercell to restore parallel alignment is also in the order of nanoseconds as in the case of $4 \times 2 \times 2$ supercell. For the "1, 3" type non-planar linkers, 180° rotational flips are rare events in the $6 \times 2 \times 2$ supercell due to the high energy barrier between the parallel and antiparallel aligned states (red regions and unsampled regions in Figure S21), similar to observations in $4 \times 2 \times 2$ supercell. From Figures S20, S21, S22 and trajectories of different chains in $6 \times 2 \times 2$ supercell we see they are always in *abcdef=PNPNPNPNPNPN...* where P = nearly planar and N = non-planar conformations which is similar to our observations for a $4 \times 2 \times 2$ supercell. Thus, the qualitative behavior of linker dynamics in the framework is well described by the $4 \times 2 \times 2$ supercell.

Statistical Analysis of "1, 3" Type Planar Linker Correlated Rotations

The model

We consider time series with either four ($4 \times 2 \times 2$ supercell) or six rotors ($6 \times 2 \times 2$ supercell). By visual inspection of the data, we see that in either case, half of the rotors are librating around an angle of ±90°, and the other rotors are rotating between angles of 0° and ±180°. It is however visually apparent that the stationary positions are not exactly 0° or 180°. In all cases there are random fluctuations (*librations*) around the means.

We will only consider the planar rotors that switch orientation, so that the number of rotors is reduced to either $M = 2$ or $M = 3$ rotors. We will impose the model that each of these rotors is either in position $x = 0$ or $x = 1$, corresponding to an angle of approximately 0° or ±180°. Henceforth we will only write angles in radial form.

Let (y_{ij}) denote the observed time series of rotor angles for rotors $j = 1, \ldots, M$ over a time series parameterized by $i = 1, \ldots, N$. They may be viewed as observations in a hidden Markov model, governed by the values of unobserved variables $x_{ij} \in \{0, 1\}$ that change dynamically

according to a Markov chain as discussed in Section .

We model the observed angle y_{ij} conditional on the unobserved value $x_{ij} \in \{0, 1\}$ as distributed according to the *von Mises distribution.* This gives for the emission probabilities the model

$$p(y_{ij} \mid \mu_{ij}, \kappa) = \frac{\exp(\kappa \cos(y_{ij} - \alpha - \mu_{ij}))}{2\pi I_0(\kappa)},$$

where $\kappa > 0$ is a concentration parameter, $\alpha \in (-\pi/2, \pi/2)$ is a single offset parameter that allows for a rotation relative to the purely vertical positions 0 and π, and where $I_0(\kappa)$ is the modified Bessel function of order zero. We assume throughout that $\kappa > 0$ is the same for all possible states. The emission angle μ_{ij} for rotor j at time i is modeled as

$$\mu_{ij} = \pi x_{ij},$$

where $x_{ij} \in \{0, 1\}$ encodes the binary state for rotor j at time i. The values of α and κ are a priori not known and need to be estimated from the data.

If we would simply categorize each observation y_{ij} as lying closer to a 0° or a 180° angle, there are two sources of problems:

- we ignore the unknown offset α which may have a significant effect in a correct classification, and

- many phantom switches (apparent switches that did in fact not happen) would be observed due to observational noise in the data. In particular during a transition from one state to another the angle may seem to switch frequently but we wish to interpret this as a single transition.

We therefore need a more careful analysis using a careful modeling of the unobserved variables x_{ij}.

Transition dynamics

In this section we discuss the dynamics of the transitions of the unobserved states x_{ij}. This will lead to the estimation of the parameters λ_{PtoAP}, λ_{APtoP} (and λ_{APtoAP}, if $M = 3$) if we would consider the values of x_{ij} to be known.

Throughout, let $K = 2^M$ denote the total number of states. There are $K = 4$ possible states if $M = 2$,

$$(0,0), \quad (0,1), \quad (1,0), \quad (1,1),$$

and $K = 8$ possible states if $M = 3$,

$$(0,0,0), \quad (0,0,1), \quad (0,1,0), \quad (0,1,1), \quad (1,0,0), \quad (1,0,1), \quad (1,1,0), \quad (1,1,1).$$

The case $M = 2$

In case $M = 2$ there are essentially two transitions: from a parallel aligned state where $x_{i1} = x_{i2}$ to a anti-parallel aligned state where $x_{i1} \neq x_{i2}$, and vice versa. We assume that from a parallel aligned state, the transition to a anti-parallel aligned state happens at rate $2\lambda_{PtoAP}$ (per unit time), so that the transition rate to a particular anti-parallel aligned state happens at rate λ_{PtoAP}. Similarly, from an anti-parallel aligned state we may transition to a parallel aligned state at total rate $2\lambda_{APtoP}$, with each possible transition occurring at rate λ_{APtoP}.

We enumerate the states as $\{0, 1, 2, 3\}$ corresponding to $(x_{i1}, x_{i2}) \in \{(0,0), (0,1), (1,0), (1,1)\}$. The Markov chain in terms of parameters λ_{PtoAP} and λ_{APtoP} is then given as

$$P = I - hG$$

where $h > 0$ denotes the length of a time interval in seconds, and

$$G = \begin{bmatrix} -2\lambda_{PtoAP} & \lambda_{PtoAP} & \lambda_{PtoAP} & 0 \\ \lambda_{APtoP} & -2\lambda_{APtoP} & 0 & \lambda_{APtoP} \\ \lambda_{APtoP} & 0 & -2\lambda_{APtoP} & \lambda_{APtoP} \\ 0 & \lambda_{PtoAP} & \lambda_{PtoAP} & -2\lambda_{PtoAP} \end{bmatrix}$$

which gives

$$P = \begin{bmatrix} 1-2h\lambda_{PtoAP} & h\lambda_{PtoAP} & h\lambda_{PtoAP} & 0 \\ h\lambda_{APtoP} & 1-2h\lambda_{APtoP} & 0 & h\lambda_{APtoP} \\ h\lambda_{APtoP} & 0 & 1-2h\lambda_{APtoP} & h\lambda_{APtoP} \\ 0 & h\lambda_{PtoAP} & h\lambda_{PtoAP} & 1-2h\lambda_{PtoAP} \end{bmatrix}.$$

Reduction to two states We may interpret the system as consisting of two states, P and AP, where P denotes the parallel aligned state and AP denotes the anti-parallel aligned state. The Markov transition matrix (for a time step $h > 0$) given by

$$P = \begin{bmatrix} 1-2h\lambda_{PtoAP} & 2h\lambda_{PtoAP} \\ 2h\lambda_{APtoP} & 1-2h\lambda_{APtoP} \end{bmatrix}.$$

Crude parameter estimation using maximum likelihood

For a sequence of observations $x_1, \ldots, x_N \in \{A, U\}$, write, for $k, \ell \in \{A, U\}$,

$$n_{k\ell} = \#\{i = 1, \ldots, n-1 : x_i = k, x_{i+1} = \ell\}.$$

For example n_{PtoAP} counts the number of transitions from P to AP. The log likelihood, conditioned on the initial state, is given by

$$\ell_c(\lambda_{PtoAP}, \lambda_{APtoP}) = n_{PP} \log(1 - 2h\lambda_{PtoAP}) + n_{PtoAP} \log \lambda_{PtoAP} \\ + n_{APtoP} \log \lambda_{APtoP} + n_{APAP} \log(1 - 2h\lambda_{APtoP}) + C,$$

where C is an additive constant that may be ignored as it does not depend on λ_{APtoP} or λ_{PtoAP}. The first order conditions with respect to λ_{PtoAP} and λ_{APtoP} are

$$\partial_{\lambda_{PtoAP}} \ell(\lambda_{PtoAP}, \lambda_{APtoP}) = \frac{n_{PtoAP}}{\lambda_{PtoAP}} - \frac{2hn_{PtoP}}{1 - 2h\lambda_{PtoAP}} = 0,$$
$$\partial_{\lambda_{APtoP}} \ell(\lambda_{PtoAP}, \lambda_{APtoP}) = \frac{n_{APtoP}}{\lambda_{APtoP}} - \frac{2hn_{APtoAP}}{1 - 2h\lambda_{APtoP}} = 0,$$

which gives the maximum likelihood estimator

$$\hat{\lambda}_{PtoAP} = \frac{n_{PtoAP}}{2h(n_{PtoP} + n_{PtoAP})}, \quad \hat{\lambda}_{APtoP} = \frac{n_{APtoP}}{2h(n_{APtoAP} + n_{APtoP})},$$

The case $M = 3$

In case $M = 3$, we either have a full parallel aligned state, where $x_{i1} = x_{i2} = x_{i3}$, or we have an antiparallel aligned state in which two states are identical and one state is different. We allow transitions:

- from parallel aligned to antiparallel aligned (total rate $3\lambda_{PtoAP}$, each possible flip at rate λ_{PtoAP}),
- from antiparallel aligned to antiparallel aligned (there are two components that can switch to have this transition, so we have total rate $2\lambda_{APtoAP}$, and a rate λ_{APtoAP} for each component to switch),
- and a single possible transition from antiparallel aligned to parallel aligned, which happens at rate λ_{APtoP}.

.

We have eight states $\{0,180\}^3$, which we enumerate as

$$\begin{gathered}(0,0,0),\quad (0,0,180),\quad (0,180,0),\quad (0,180,180),\\ (180,0,0),\quad (180,0,180),\quad (180,180,0),\quad (180,180,180).\end{gathered}$$

The generator in terms of these states is

$$G=\begin{bmatrix} -3\lambda_{PtoAP} & \lambda_{PtoAP} & \lambda_{PtoAP} & 0 & \lambda_{PtoAP} & 0 & 0 & 0 \\ \lambda_{APtoP} & \begin{matrix}-\lambda_{APtoP}\\ -2\lambda_{APtoAP}\end{matrix} & 0 & \lambda_{APtoAP} & 0 & \lambda_{APtoAP} & 0 & 0 \\ \lambda_{APtoP} & 0 & \begin{matrix}-\lambda_{APtoP}\\ -2\lambda_{APtoAP}\end{matrix} & \lambda_{APtoAP} & 0 & 0 & \lambda_{APtoAP} & 0 \\ 0 & \lambda_{APtoAP} & \lambda_{APtoAP} & \begin{matrix}-\lambda_{APtoP}\\ -2\lambda_{APtoAP}\end{matrix} & 0 & 0 & 0 & \lambda_{APtoP} \\ \lambda_{APtoP} & 0 & 0 & 0 & \begin{matrix}-\lambda_{APtoP}\\ -2\lambda_{APtoAP}\end{matrix} & \lambda_{APtoAP} & \lambda_{APtoAP} & 0 \\ 0 & \lambda_{APtoAP} & 0 & 0 & \lambda_{APtoAP} & \begin{matrix}-\lambda_{APtoP}\\ -2\lambda_{APtoAP}\end{matrix} & 0 & \lambda_{APtoP} \\ 0 & 0 & \lambda_{APtoAP} & 0 & \lambda_{APtoAP} & 0 & \begin{matrix}-\lambda_{APtoP}\\ -2\lambda_{APtoAP}\end{matrix} & \lambda_{APtoP} \\ 0 & 0 & 0 & \lambda_{PtoAP} & 0 & \lambda_{PtoAP} & \lambda_{PtoAP} & -3\lambda_{PtoAP} \end{bmatrix}$$

with associated transition matrix $P = I - hG$.

Similarly to the case $M = 2$, we count transitions as follows:

- n_{PP}: stay in parallel aligned state
- n_{PtoAP}: move from parallel aligned to anti-parallel aligned state
- n_{APtoP}: move from anti-parallel aligned to parallel aligned
- $n_{AP\Delta AP}$: move from an anti-parallel aligned state to a *different* anti-parallel aligned state
- n_{APAP}: stay in a particular unaligned state

The log likelihood is given as

$$\begin{aligned}\ell_c(\lambda_{PtoAP}, \lambda_{APtoP}, \lambda_{APtoAP}) = n_{PP}\log(1-3\lambda_{PtoAP}h) + n_{PtoAP}\log(3\lambda_{PtoAP}h)\\ + n_{APtoP}\log\lambda_{APtoP} + n_{AP\Delta AP}\log(2\lambda_{APtoAP})\\ + n_{APAP}\log(1-h(\lambda_{APtoP}+2\lambda_{APtoAP}))\end{aligned} \tag{10}$$

Differentiation with respect to λ_{PtoAP}, λ_{APtoP} and λ_{APtoAP} gives

$$\begin{aligned}\partial_{\lambda_{PtoAP}}\ell_c(\lambda_{PtoAP}, \lambda_{APtoP}, \lambda_{APtoAP}) &= -\frac{3hn_{PP}}{1-3\lambda_{PtoAP}h} + \frac{n_{PtoAP}}{\lambda_{PtoAP}} = 0\\ \partial_{\lambda_{APtoP}}\ell_c(\lambda_{PtoAP}, \lambda_{APtoP}, \lambda_{APtoAP}) &= \frac{n_{APtoP}}{\lambda_{APtoP}} - \frac{hn_{APAP}}{1-h(\lambda_{APtoP}+2\lambda_{APtoAP})} = 0,\\ \partial_{\lambda_{APtoAP}}\ell_c(\lambda_{PtoAP}, \lambda_{APtoP}, \lambda_{APtoAP}) &= \frac{n_{AP\Delta AP}}{\lambda_{APtoAP}} - \frac{2hn_{APAP}}{1-h(\lambda_{APtoP}+2\lambda_{APtoAP})} = 0\end{aligned}$$

After rearranging this gives a linear system in λ_{APtoP}, λ_{PtoAP} and λ_{APtoAP}, with solution

$$\hat{\lambda}_{PtoAP} = \frac{n_{AU}}{3h(n_{PP}+n_{PtoAP})}$$

$$\begin{aligned}\hat{\lambda}_{APtoP} &= \frac{n_{APtoP}}{h(n_{APtoP}+n_{APAP}+n_{AP\Delta AP})}\\ \hat{\lambda}_{APtoAP} &= \frac{n_{AP\Delta AP}}{2h(n_{APtoP}+n_{APAP}+n_{AP\Delta AP})}\end{aligned}$$

EM-approach, Baum-Welch algorithm

A careful approach for estimating the transition rates requires that we take into account the uncertainty in the estimation of the unobserved states x_{ij}. This gives a hierarchical model and it is the Expectation-Maximization (EM)-algorithm[21,22] that takes care of the estimation of the parameter λ (consisting of λ_{PtoAP}, λ_{APtoP} and, if $M = 3$, λ_{APtoAP}), as well as the parameters α and κ. The EM-algorithm iteratively determines a new approximation of the maximum likelihood equations in the E-step, and solves these in the M-step. For an introduction to this algorithm see[21].

In the Expectation (E)-step, we determine the conditional distribution of the states (x_i) given observations (y_i) and parameters $\theta' = (\kappa', \lambda', \alpha')$, to give the expected complete likelihood $Q(\theta, \theta')$. In the Maximimization (M)-step we maximize $Q(\theta, \theta')$ with respect to $\theta = (\kappa, \lambda, \alpha)$.

The E-step may be carried out using the Baum-Welch algorithm, as described in[21], and we refer the readers to that reference and the computer code for details. We will now focus on the M-step of the algorithm.

The EM algorithm then works by alternating the E and M step for a fixed number of iterations.

M-step

Let (x_i) denote the hidden Markov chain with values in $1, \ldots, K$. Conditional on x_i, we have emission probabilities

$$p(y_{ij} \mid x_i, \kappa, \alpha) = \frac{\exp(\kappa \cos(\mu_{i,j} + \alpha - y_{ij}))}{2\pi I_0(\kappa)}.$$

The complete data log likelihood decomposes as

$$\ell(\kappa, \alpha, \lambda; (x_i), (_i)) = \sum_{i=1}^{N} \sum_{j=1}^{M} \log p(y_{ij} \mid x_i = k, \kappa, \alpha) + \sum_{i=1}^{N-1} \log p(x_{i+1} \mid x_i; \lambda).$$

For a sequence of state probabilities (γ_{ik}) and transition probabilities $(\xi_{ik\ell})$ (see section 13.2 in[21]), we have expected log likelihood

$$q(\kappa, \alpha, \lambda) = q_1(\kappa, \alpha) + q_2(\lambda),$$

where

$$
\begin{aligned}
q_1(\kappa, \alpha) &= \sum_{i=1}^{N} \sum_{k=1}^{K} \gamma_{ik} \log p(_i \mid _i = j, \kappa, \alpha) \\
&= \sum_{i=1}^{N} \sum_{k=1}^{K} \gamma_{ik} \left(\kappa \sum_{j=1}^{M} \cos(y_{ij} - \mu_{jk} - \alpha) - M \log 2\pi - M \log I_0(\kappa) \right).
\end{aligned}
$$

and

$$
q_2(\lambda) = \sum_{i=1}^{N-1} \sum_{k=1}^{K} \sum_{\ell=1}^{K} \xi_{ik\ell} \log P_\lambda(k, \ell).
$$

The first order condition with respect to α is

$$
\sum_{i=1}^{N} \sum_{k=1}^{K} \gamma_{ik} \sum_{j=1}^{M} \sin(y_{ij} - \mu_{jk} - \alpha) = 0,
$$

which is solved by setting

$$
\hat{\alpha} = \arg \sum_{i=1}^{N} \sum_{k=1}^{K} \sum_{j=1}^{M} \gamma_{ik} \exp\left(\mathrm{i}[y_{ij} - \mu_{jk}]\right).
$$

The first order condition with respect to κ is

$$
\sum_{i=1}^{N} \sum_{k=1}^{K} \gamma_{ik} \left(\sum_{j=1}^{M} \cos(y_{ij} - \mu_{jk}) - M \frac{d}{d\kappa} \log I_0(\kappa) \right) = 0.
$$

Solving for κ gives the implicit equation to be solved for κ,

$$
\frac{d}{d\kappa} \log I_0(\hat{\kappa}) = \frac{\sum_{i=1}^{N} \sum_{k=1}^{K} \gamma_{ik} \sum_{j=1}^{M} \cos(y_{ij} - \hat{\mu}_{jk})}{M \sum_{i=1}^{N} \sum_{k=1}^{K} \gamma_{ik}} =: \overline{R}_\gamma.
$$

Following[23], we introduce the functions $A_p(\kappa) = I_{p/2}(\kappa)/I_{p/2-1}(\kappa)$. We then have that

$$
\frac{d}{d\kappa} \log I_0(\kappa) = \frac{I_1(\kappa)}{I_0(\kappa)} = A_2(\kappa).
$$

and, as it turns out[23],

$$\frac{d}{d\kappa}A_p(\kappa) = 1 - A_p(\kappa)^2 - \frac{p-1}{\kappa}A_p(\kappa).$$

One step of the Newton-Raphson is therefore given by

$$\kappa^{(\ell+1)} = \kappa^{(\ell)} - \frac{A_2(\kappa^{(\ell)}) - \overline{R}_\gamma}{1 - A_2(\kappa^{(\ell)})^2 - \frac{1}{\kappa^{(\ell)}}A_2(\kappa^{(\ell)}}.$$

A good starting point for the iterations is provided by

$$\kappa^{(0)} = \frac{\overline{R}_\gamma(2 - \overline{R}_u^2)}{1 - \overline{R}_\gamma^2}.$$

Finally for the Markov transition probabilities we have the first order condition

$$\sum_{i=1}^{N-1}\sum_{k=1}^{K}\sum_{\ell=1}^{K}\xi_{ik\ell}\nabla_\lambda P_\lambda(k,\ell)/P_\lambda(k,\ell) = 0. \tag{11}$$

The case $M = 2$

In the two-rotor case, we have (approximately)

$$P_\lambda = \begin{bmatrix} 1-2h\lambda_{PtoAP} & h\lambda_{PtoAP} & h\lambda_{PtoAP} & 0 \\ h\lambda_{APtoP} & 1-2h\lambda_{APtoP} & 0 & h\lambda_{APtoP} \\ h\lambda_{APtoP} & 0 & 1-2h\lambda_{APtoP} & h\lambda_{APtoP} \\ 0 & h\lambda_{PtoAP} & h\lambda_{PtoAP} & 1-2h\lambda_{PtoAP} \end{bmatrix}.$$

Consequently,

$$\partial_{\lambda_{PtoAP}} P_\lambda = \begin{bmatrix} -2h & h & h & 0 \\ 0 & 0 & 0 & 0 \\ 0 & 0 & 0 & 0 \\ 0 & h & h & -2h \end{bmatrix}, \quad \partial_{\lambda_{APtoP}} P_\lambda = \begin{bmatrix} 0 & 0 & 0 & 0 \\ h & -2h & 0 & h \\ h & 0 & -2h & h \\ 0 & 0 & 0 & 0 \end{bmatrix}.$$

Then (11) gives the equations

$$\sum_{i=1}^{N-1} (\xi_{i,1,1} + \xi_{i,4,4}) \frac{-2h}{1 - 2h\lambda_{PtoAP}} + (\xi_{i,1,2} + \xi_{i,1,3} + \xi_{i,4,2} + \xi_{i,4,3}) \frac{h}{h\lambda_{PtoAP}} = 0,$$
$$\sum_{i=1}^{N-1} (\xi_{i,2,2} + \xi_{i,3,3}) \frac{-2h}{1 - 2h\lambda_{APtoP}} + (\xi_{i,2,1} + \xi_{i,2,4} + \xi_{i,3,1} + \xi_{i,3,4}) \frac{h}{h\lambda_{APtoP}} = 0,$$

resulting in

$$\lambda_{PtoAP} = \frac{\sum_{i=1}^{N-1} (\xi_{i,1,2} + \xi_{i,1,3} + \xi_{i,4,2} + \xi_{i,4,3})}{2h \sum_{i=1}^{N-1} (\xi_{i,1,1} + \xi_{i,4,4} + \xi_{i,1,2} + \xi_{i,1,3} + \xi_{i,4,2} + \xi_{i,4,3})},$$
$$\lambda_{APtoP} = \frac{\sum_{i=1}^{N-1} (\xi_{i,2,1} + \xi_{i,2,4} + \xi_{i,3,1} + \xi_{i,3,4})}{2h \sum_{i=1}^{N-1} (\xi_{i,2,2} + \xi_{i,3,3} + \xi_{i,2,1} + \xi_{i,2,4} + \xi_{i,3,1} + \xi_{i,3,4})}.$$

The case $M = 3$

The case $M = 3$ may be handled analogously; for details see the computer code.

Numerical comparison

In this section we briefly compare the crude MLE-based estimation (which is also used as initialization of the EM-algorithm) with the EM-approach. In both cases we have to decide on the sub sampling used (i.e. whether we take every element, or every 10th/100th/1000th element of the data) when processing the data. The amount of sub sampling of the data strongly affects the estimated parameters when using the 'crude' maximum likelihood method. A likely explanation is that once a transition occurs it takes a few time steps in which there is

even some back and forth, which leads incorrectly to a large transition count. This is exactly the motivation for employing the EM-approach, as it takes care of this uncertainty.

Two rotors

In the initialization, the number of transitions n_{AU}, n_{AA}, n_{UA}, n_{UU} is estimated by interpreting every position as either unambiguously A or U based on the relative angle (ignoring effects of noise). Subsequently the MLE approach to parameter estimation is carried out as described in the previous section

	initialization		result EM	
subsampling rate	λ_{PtoAP}	λ_{APtoP}	λ_{PtoAP}	λ_{APtoP}
1	0.512	2.03	0.0897	0.385
10	0.197	0.805	0.0898	0.385
100	0.0897	0.385	0.0898	0.385
1000	0.0898	0.386	0.0808	0.354

Three rotors

Here, we simply use the initialization by maximum likelihood.

	initialization			result EM		
subsampling rate	λ_{PtoAP}	λ_{APtoP}	λ_{APtoAP}	λ_{PtoAP}	λ_{APtoP}	λ_{APtoAP}
1	0.521	2.744	2.266	0.0447	0.199	0.159
10	0.246	1.272	0.755	0.0447	0.199	0.159
100	0.0894	0.477	0.239	0.0372	0.159	0.159
1000	0.0372	0.159	0.159	0.0373	0.160	0.158

References

(1) Vanduyfhuys, L.; Vandenbrande, S.; Wieme, J.; Waroquier, M.; Verstraelen, T.; Van Speybroeck, V. Extension of the QuickFF force field protocol for an improved accuracy of structural, vibrational, mechanical and thermal properties of metal-organic frameworks. *Journal of Computational Chemistry* **2018**, *39*, 999–1011.

(2) Kresse, G.; Furthmüller, J. Efficient iterative schemes for ab initio total-energy calculations using a plane-wave basis set. *Physical Review B* **1996**, *54*, 11169–11186.

(3) Kresse, G.; Joubert, D. From ultrasoft pseudopotentials to the projector augmented-wave method. *Physical Review B* **1999**, *59*, 1758–1775.

(4) Perdew, J. P.; Burke, K.; Ernzerhof, M. Generalized Gradient Approximation Made Simple. *Physical Review Letters* **1996**, *77*, 3865–3868.

(5) Grimme, S.; Antony, J.; Ehrlich, S.; Krieg, H. A consistent and accurate ab initio parametrization of density functional dispersion correction (DFT-D) for the 94 elements H-Pu. *The Journal of Chemical Physics* **2010**, *132*, 154104.

(6) Grimme, S.; Ehrlich, S.; Goerigk, L. Effect of the damping function in dispersion corrected density functional theory. *Journal of Computational Chemistry* **2011**, *32*, 1456–1465.

(7) Blöchl, P. E. Projector augmented-wave method. *Physical Review B* **1994**, *50*, 17953–17979.

(8) Verstraelen, T.; Vandenbrande, S.; Heidar-Zadeh, F.; Vanduyfhuys, L.; Van Speybroeck, V.; Waroquier, M.; Ayers, P. W. Minimal Basis Iterative Stockholder: Atoms in Molecules for Force-Field Development. *Journal of Chemical Theory and Computation* **2016**, *12*, 3894–3912.

(9) Verstraelen, T. DensPart (https://github.com/theochem/denspart). `https://github.com/theochem/denspart`.

(10) Enkovaara, J.; Rostgaard, C.; Mortensen, J. J.; Chen, J.; Dułak, M.; Ferrighi, L.; Gavnholt, J.; Glinsvad, C.; Haikola, V.; Hansen, H. A. *et al.* Electronic structure calculations with GPAW: a real-space implementation of the projector augmented-wave method. *Journal of Physics: Condensed Matter* **2010**, *22*, 253202.

(11) Mortensen, J. J.; Hansen, L. B.; Jacobsen, K. W. Real-space grid implementation of the projector augmented wave method. *Physical Review B* **2005**, *71*, 035109.

(12) Larsen, A. H.; Mortensen, J. J.; Blomqvist, J.; Castelli, I. E.; Christensen, R.; Dułak, M.; Friis, J.; Groves, M. N.; Hammer, B.; Hargus, C. *et al.* The atomic simulation environment—a Python library for working with atoms. *Journal of Physics: Condensed Matter* **2017**, *29*, 273002.

(13) Allinger, N. L.; Zhou, X.; Bergsma, J. Molecular mechanics parameters. *Journal of Molecular Structure: THEOCHEM* **1994**, *312*, 69–83.

(14) Yaff, yet another force field; https://github.com/molmod/molmod. `https://github.com/molmod/molmod`.

(15) Ghysels, A.; Verstraelen, T.; Hemelsoet, K.; Waroquier, M.; Van Speybroeck, V. TAMkin: A Versatile Package for Vibrational Analysis and Chemical Kinetics. *Journal of Chemical Information and Modeling* **2010**, *50*, 1736–1750.

(16) Gonzalez-Nelson, A.; Mula, S.; Šimėnas, M.; Balčiu nas, S.; Altenhof, A. R.; Vojvodin, C. S.; Canossa, S.; Banys, J.; Schurko, R. W.; Coudert, F.-X. *et al.* Emergence of Coupled Rotor Dynamics in Metal–Organic Frameworks via Tuned Steric Interactions. *Journal of the American Chemical Society* **2021**, *143*, 12053–12062, Publisher: American Chemical Society.

(17) Thompson, A. P.; Aktulga, H. M.; Berger, R.; Bolintineanu, D. S.; Brown, W. M.; Crozier, P. S.; in 't Veld, P. J.; Kohlmeyer, A.; Moore, S. G.; Nguyen, T. D. *et al.* LAMMPS - a flexible simulation tool for particle-based materials modeling at the atomic, meso, and continuum scales. *Computer Physics Communications* **2022**, *271*, 108171.

(18) Shire, T.; Hanley, K. J.; Stratford, K. DEM simulations of polydisperse media: efficient contact detection applied to investigate the quasi-static limit. *Computational Particle Mechanics* **2021**, *8*, 653–663.

(19) in 't Veld, P. J.; Plimpton, S. J.; Grest, G. S. Accurate and efficient methods for modeling colloidal mixtures in an explicit solvent using molecular dynamics. *Computer Physics Communications* **2008**, *179*, 320–329.

(20) Biswas, S.; Ahnfeldt, T.; Stock, N. New Functionalized Flexible Al-MIL-53-X (X = -Cl, -Br, -CH 3 , -NO 2 , -(OH) 2) Solids: Syntheses, Characterization, Sorption, and Breathing Behavior. *Inorganic Chemistry* **2011**, *50*, 9518–9526.

(21) Bishop, C. M. *Pattern recognition and machine learning*; Information science and statistics; Springer: New York, 2006.

(22) Dempster, A. P.; Laird, N. M.; Rubin, D. B. Maximum Likelihood from Incomplete Data Via the *EM* Algorithm. *Journal of the Royal Statistical Society: Series B (Methodological)* **1977**, *39*, 1–22.

(23) Sra, S. A short note on parameter approximation for von Mises-Fisher distributions: and a fast implementation of I s (x). *Computational Statistics* **2012**, *27*, 177–190.
