# Supplementary figures and images for: Long-Range and Coupled Rotor Dynamics in NO2-MIL-53(Al) by Classical Molecular Dynamics
Source: J Phys Chem C Nanomater Interfaces. 2024 Nov 12;128(47):20264–74. doi: 10.1021/acs.jpcc.4c05851 (PMC11613561; doi:10.1021/acs.jpcc.4c05851)

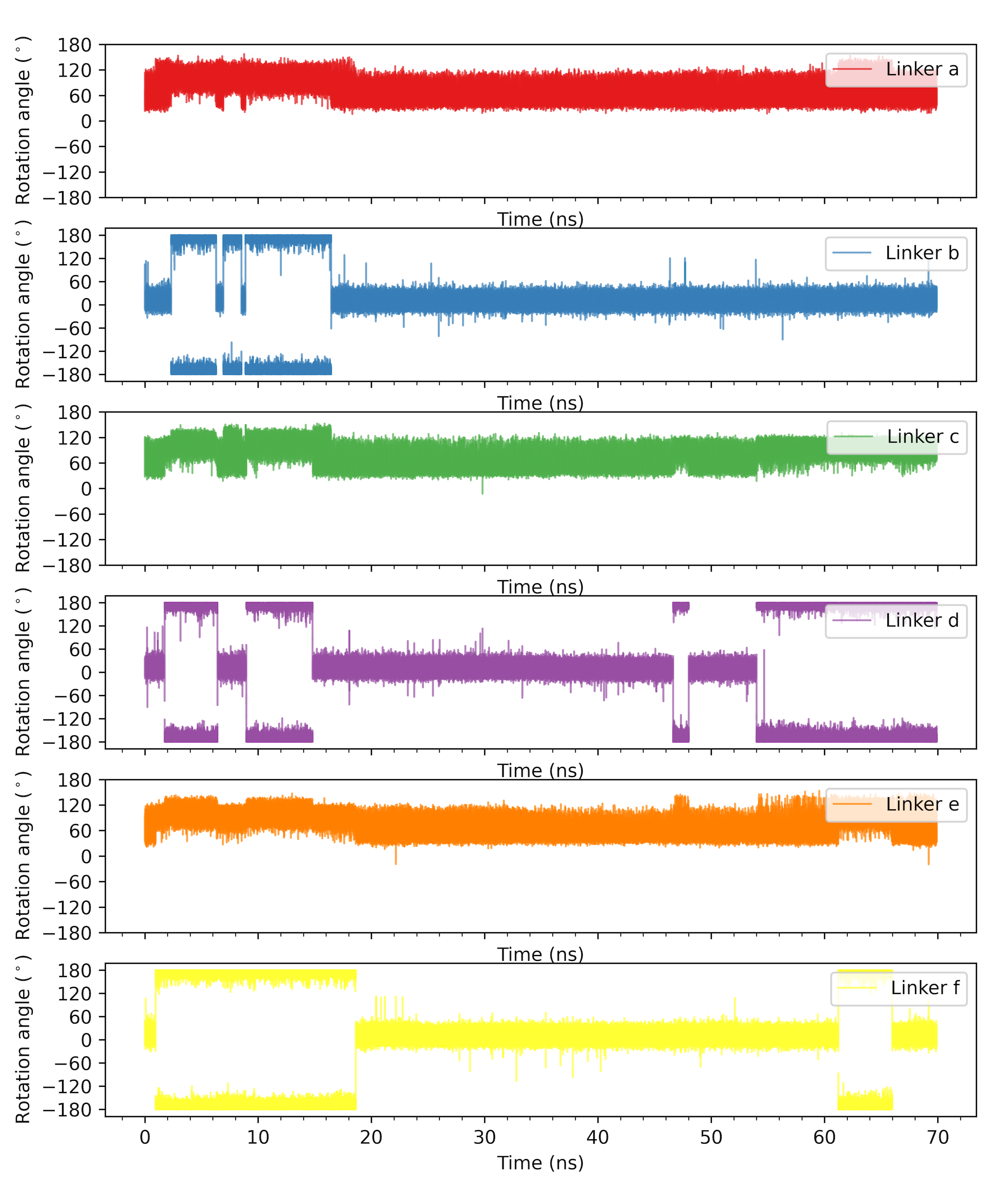

Supplement: Supplementary file 3 — jp4c05851_si_003.zip [file jp4c05851_si_003.zip › Trajectoryplots/622 supercell/622_Chain8.png]

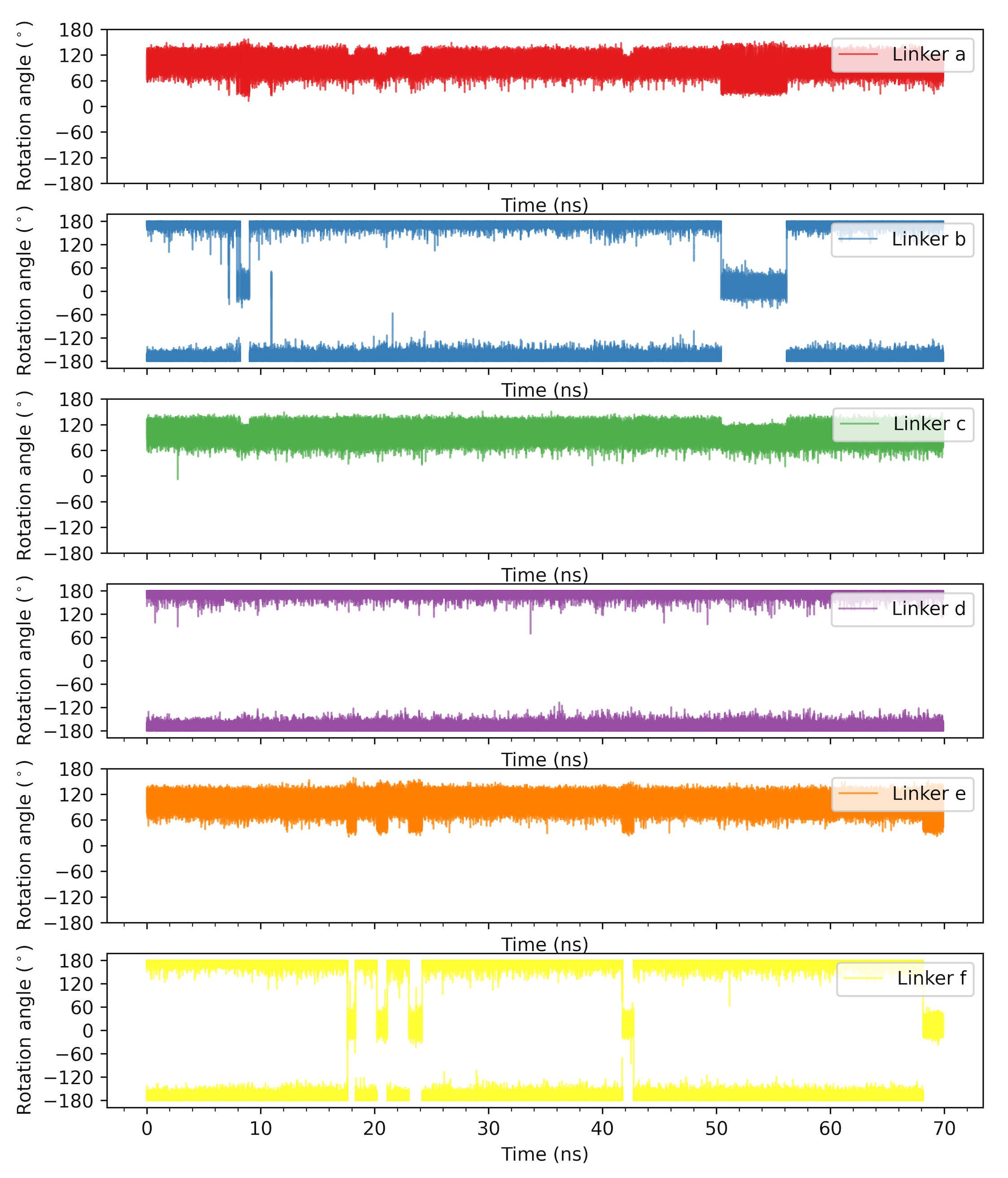

Supplement: Supplementary file 3 — jp4c05851_si_003.zip [file jp4c05851_si_003.zip › Trajectoryplots/622 supercell/622_Chain9.png]

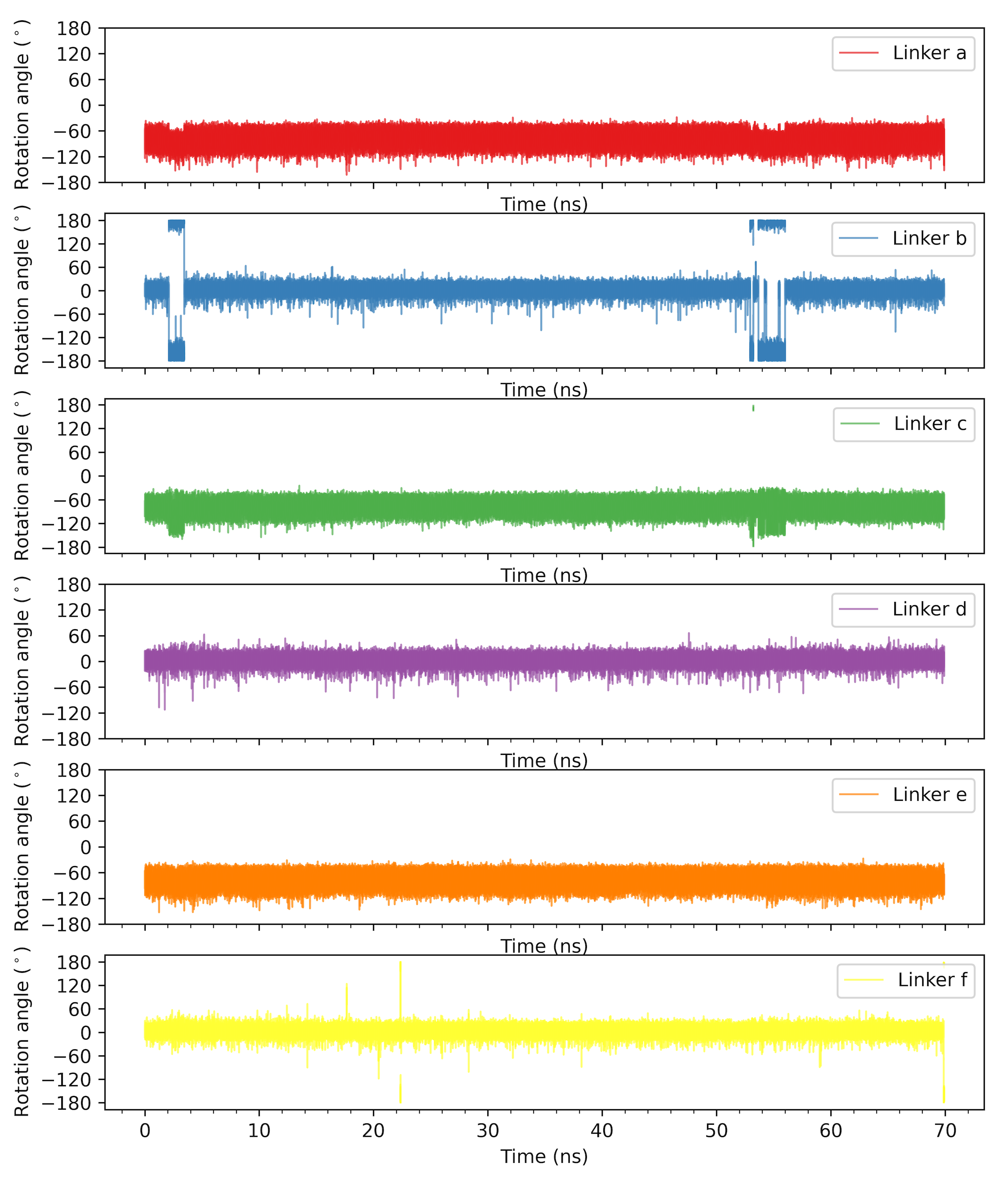

Supplement: Supplementary file 3 — jp4c05851_si_003.zip [file jp4c05851_si_003.zip › Trajectoryplots/622 supercell/622_Chain16.png]

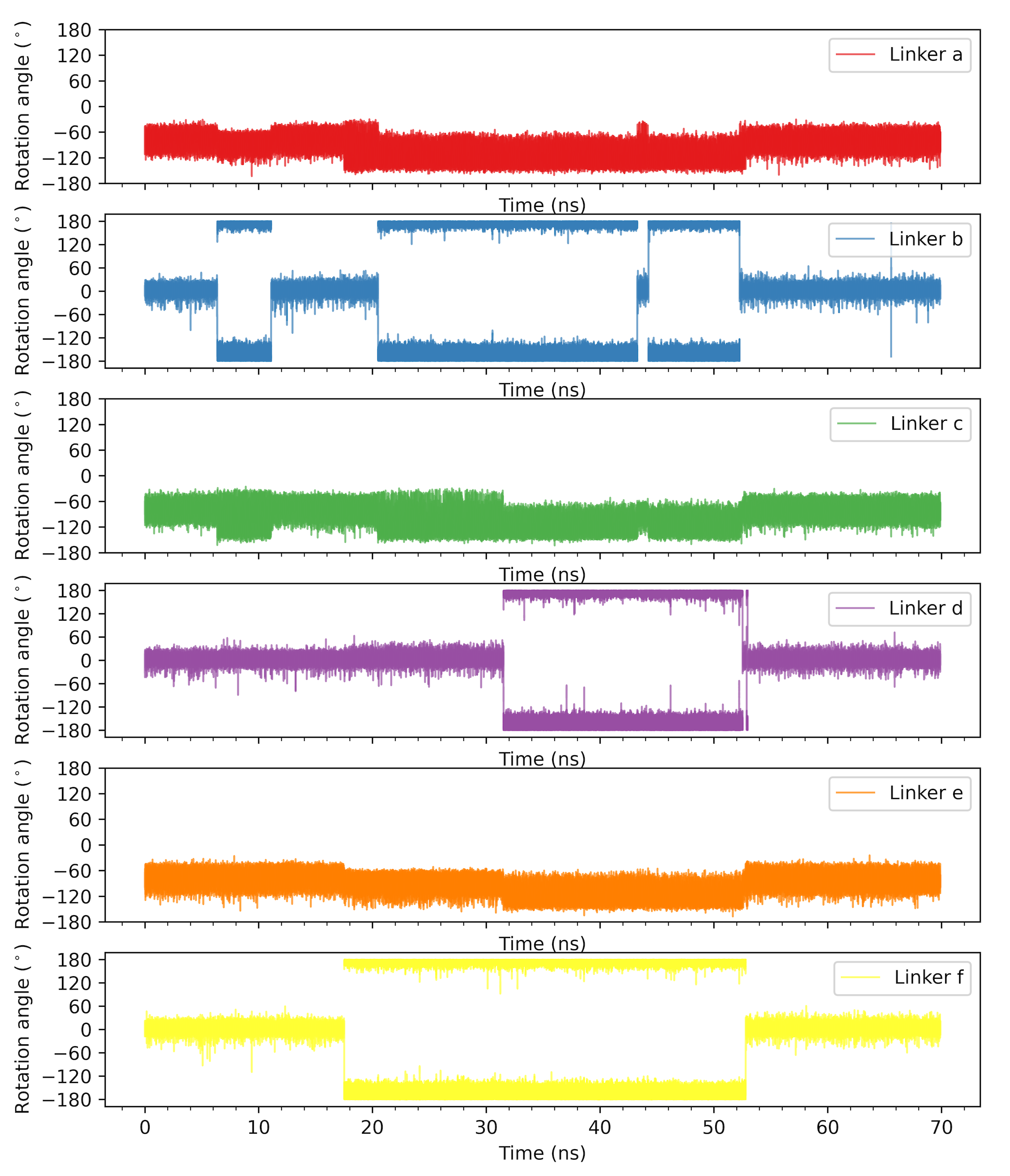

Supplement: Supplementary file 3 — jp4c05851_si_003.zip [file jp4c05851_si_003.zip › Trajectoryplots/622 supercell/622_Chain15.png]

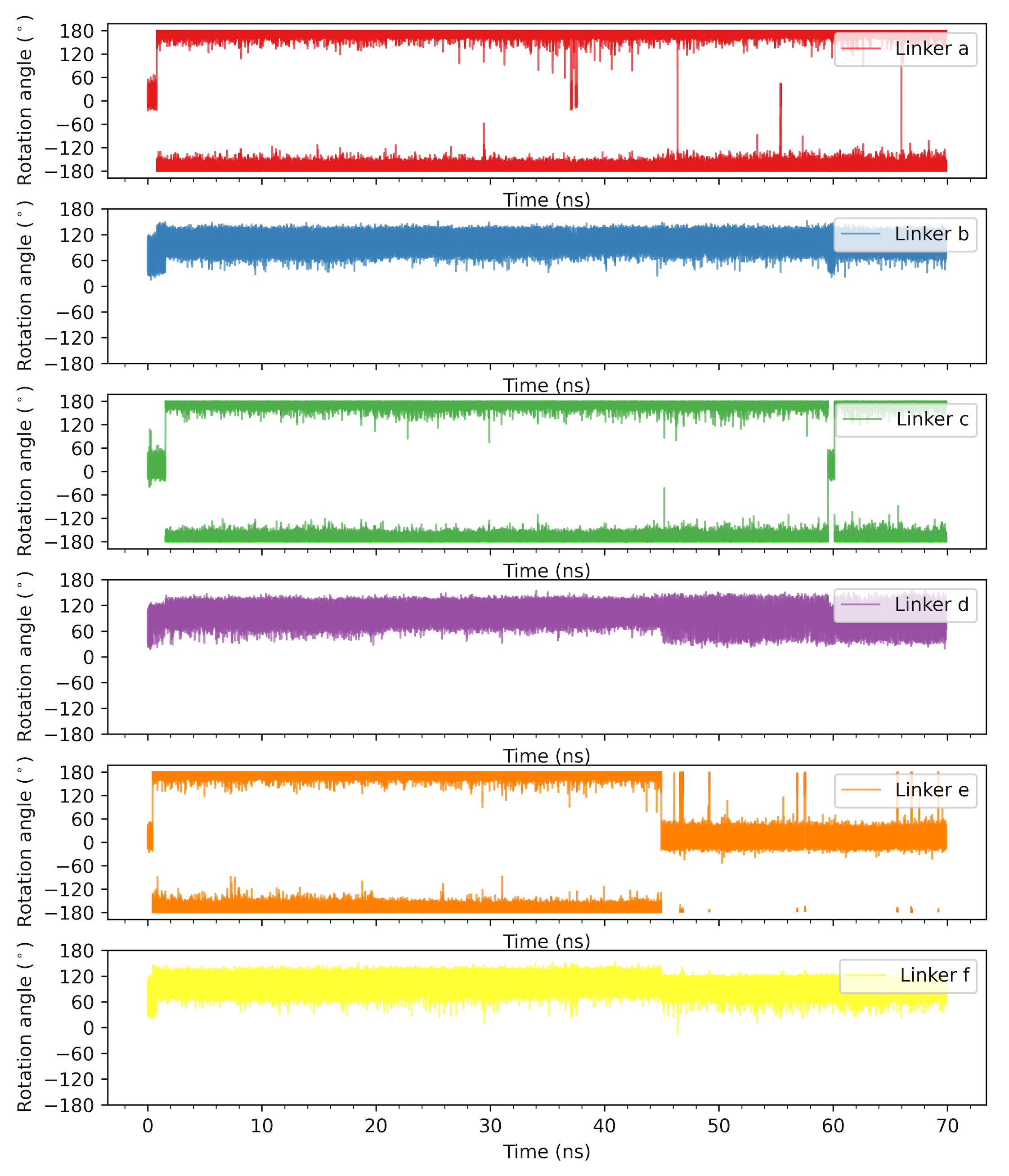

Supplement: Supplementary file 3 — jp4c05851_si_003.zip [file jp4c05851_si_003.zip › Trajectoryplots/622 supercell/622_Chain14.png]

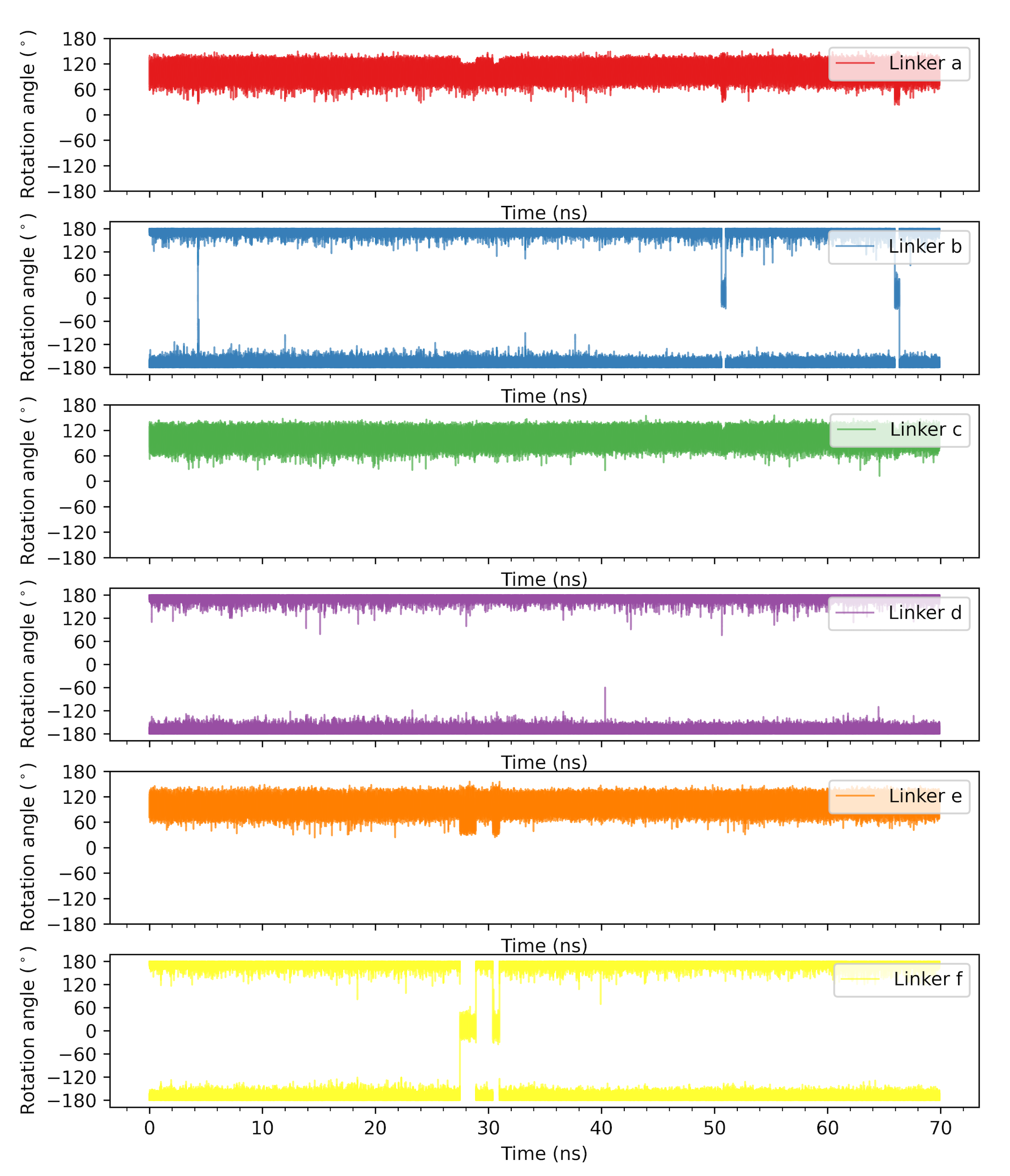

Supplement: Supplementary file 3 — jp4c05851_si_003.zip [file jp4c05851_si_003.zip › Trajectoryplots/622 supercell/622_Chain10.png]

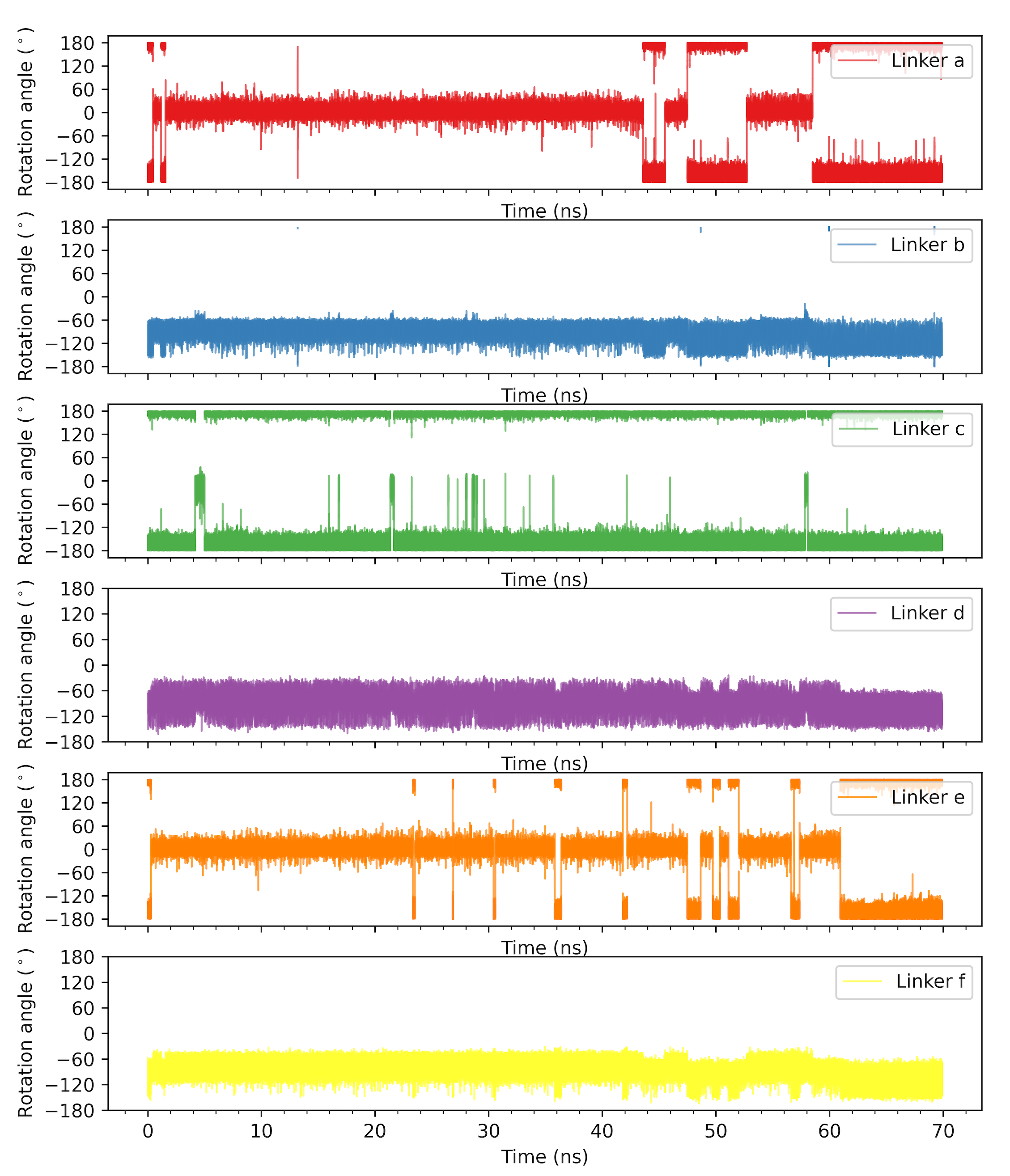

Supplement: Supplementary file 3 — jp4c05851_si_003.zip [file jp4c05851_si_003.zip › Trajectoryplots/622 supercell/622_Chain11.png]

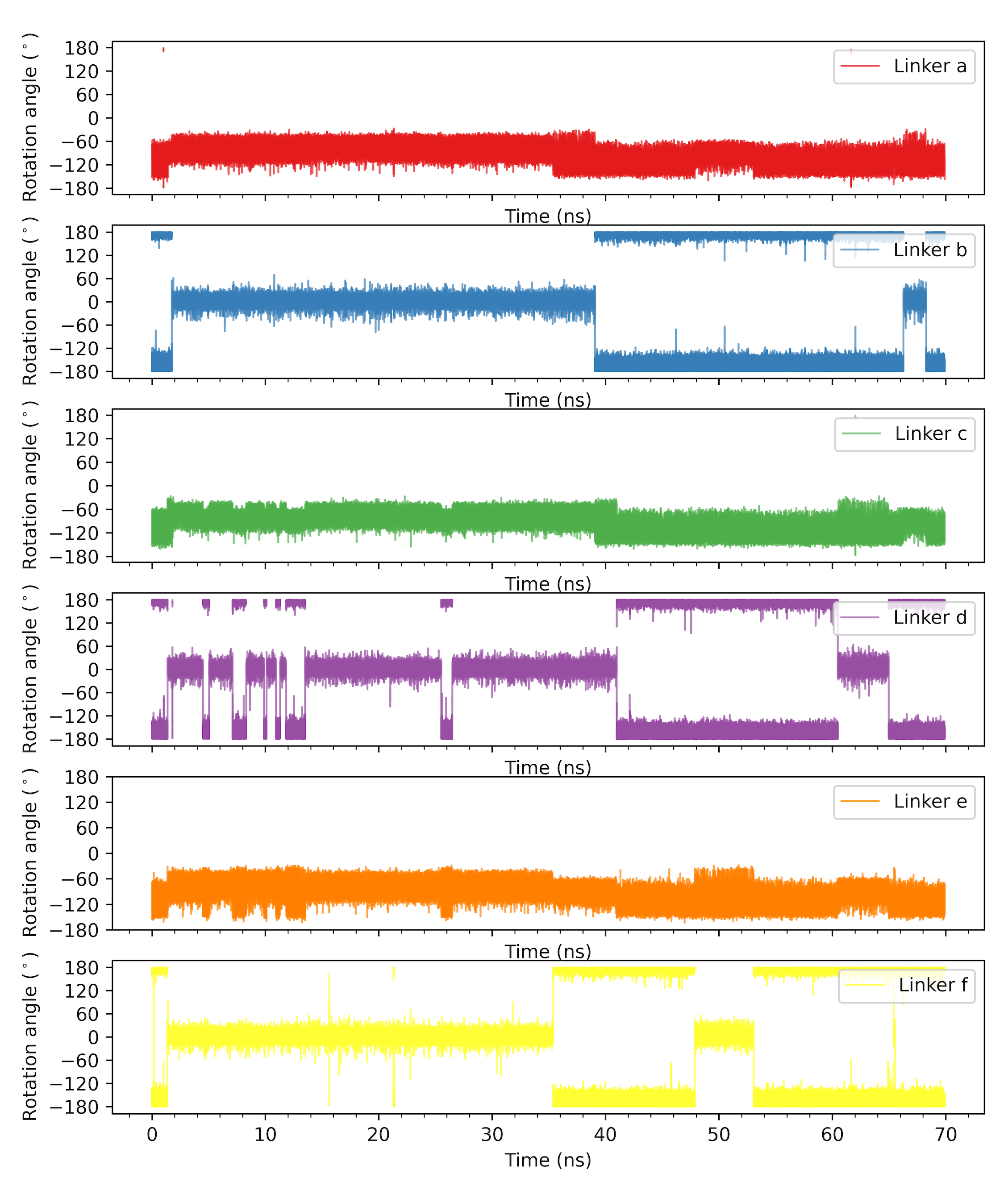

Supplement: Supplementary file 3 — jp4c05851_si_003.zip [file jp4c05851_si_003.zip › Trajectoryplots/622 supercell/622_Chain13.png]

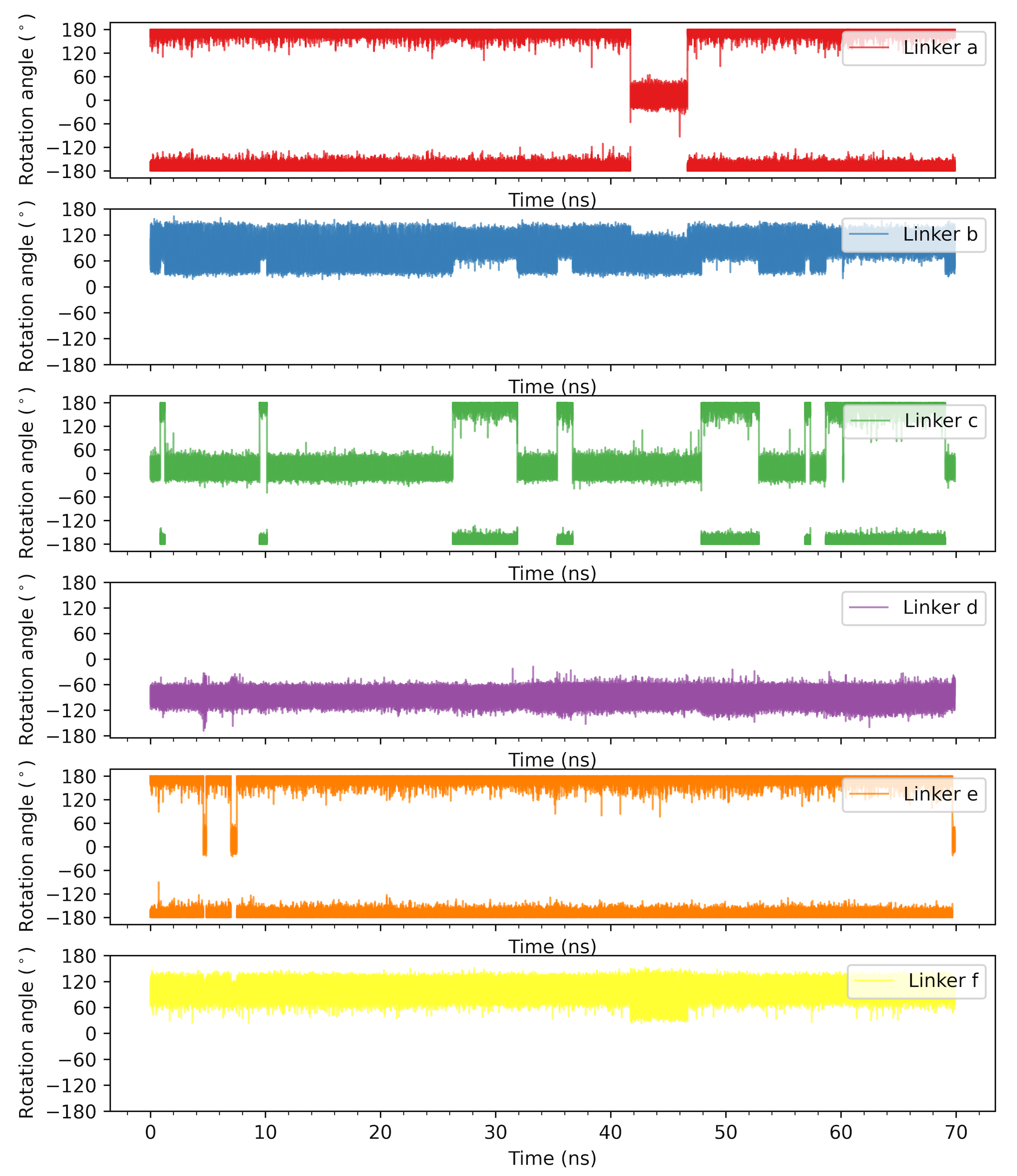

Supplement: Supplementary file 3 — jp4c05851_si_003.zip [file jp4c05851_si_003.zip › Trajectoryplots/622 supercell/622_Chain12.png]

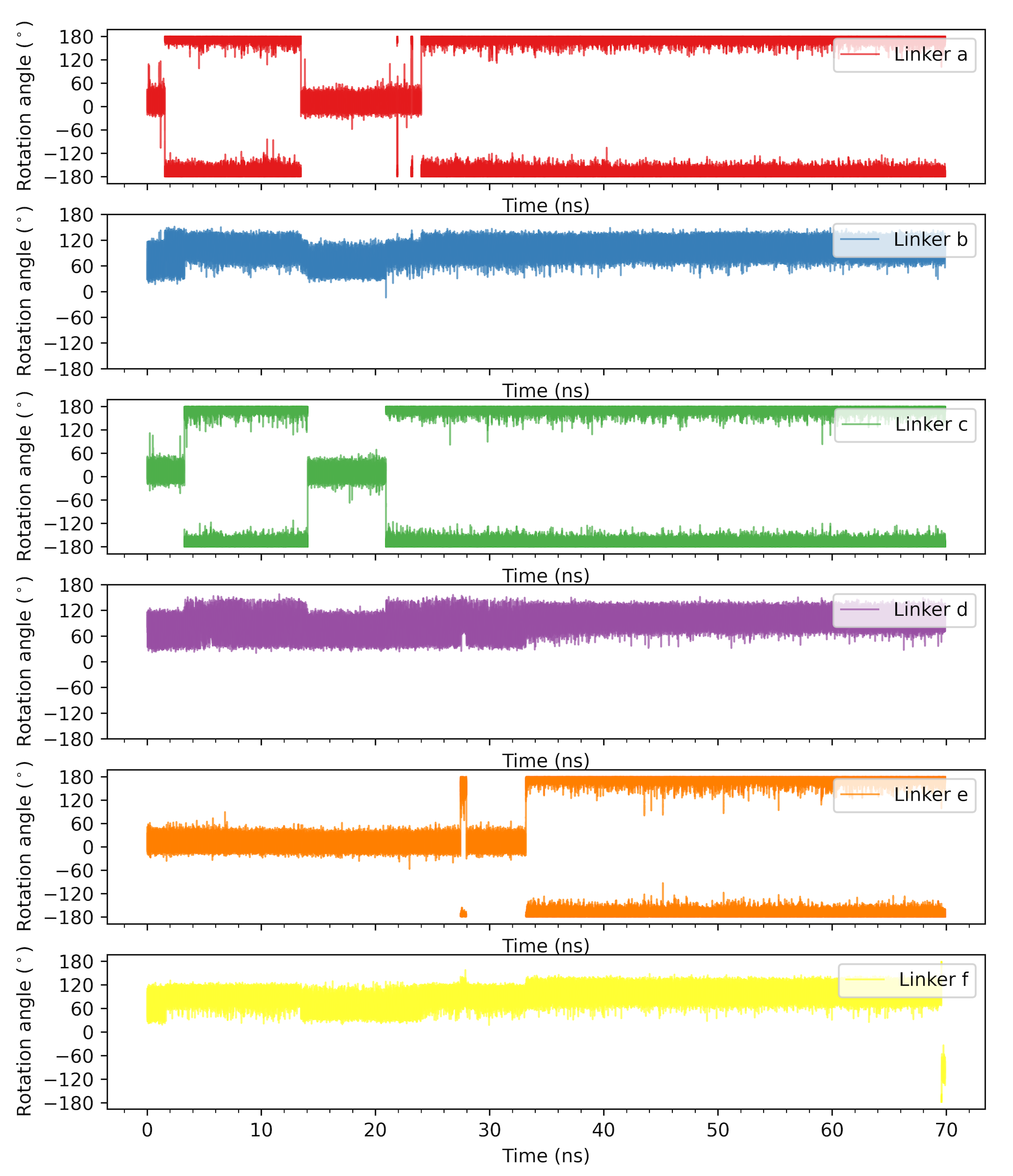

Supplement: Supplementary file 3 — jp4c05851_si_003.zip [file jp4c05851_si_003.zip › Trajectoryplots/622 supercell/622_Chain7.png]

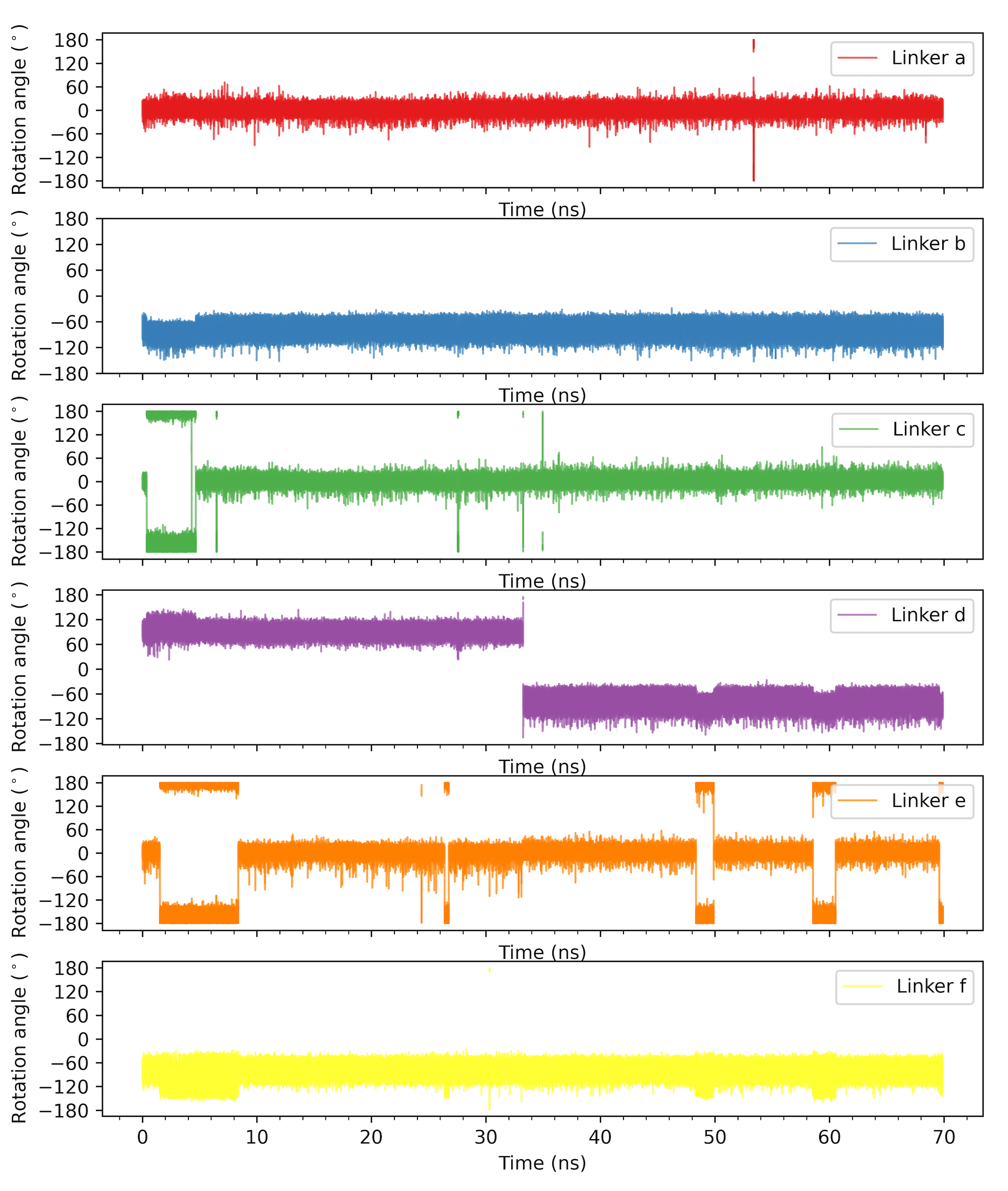

Supplement: Supplementary file 3 — jp4c05851_si_003.zip [file jp4c05851_si_003.zip › Trajectoryplots/622 supercell/622_Chain6.png]

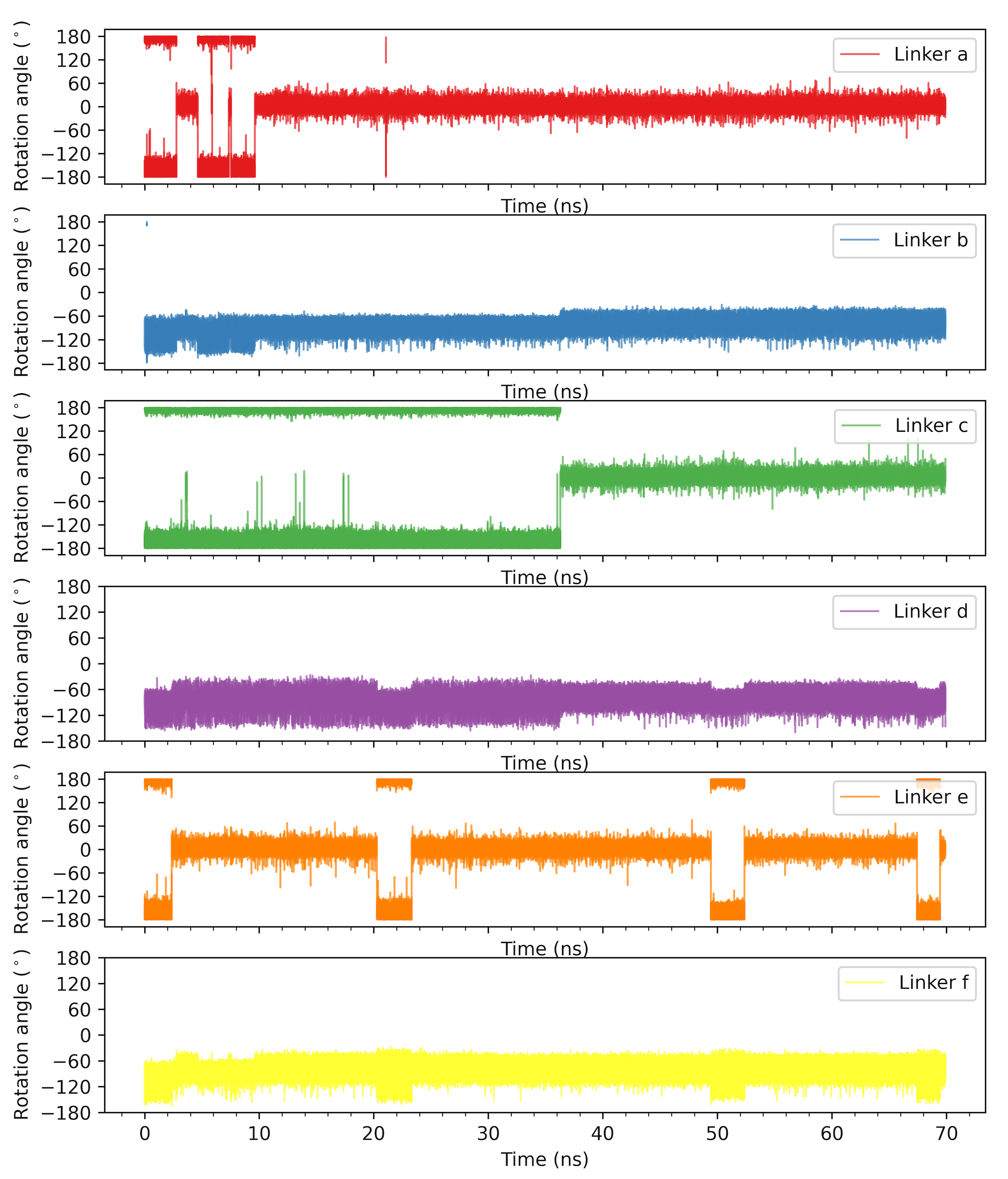

Supplement: Supplementary file 3 — jp4c05851_si_003.zip [file jp4c05851_si_003.zip › Trajectoryplots/622 supercell/622_Chain4.png]

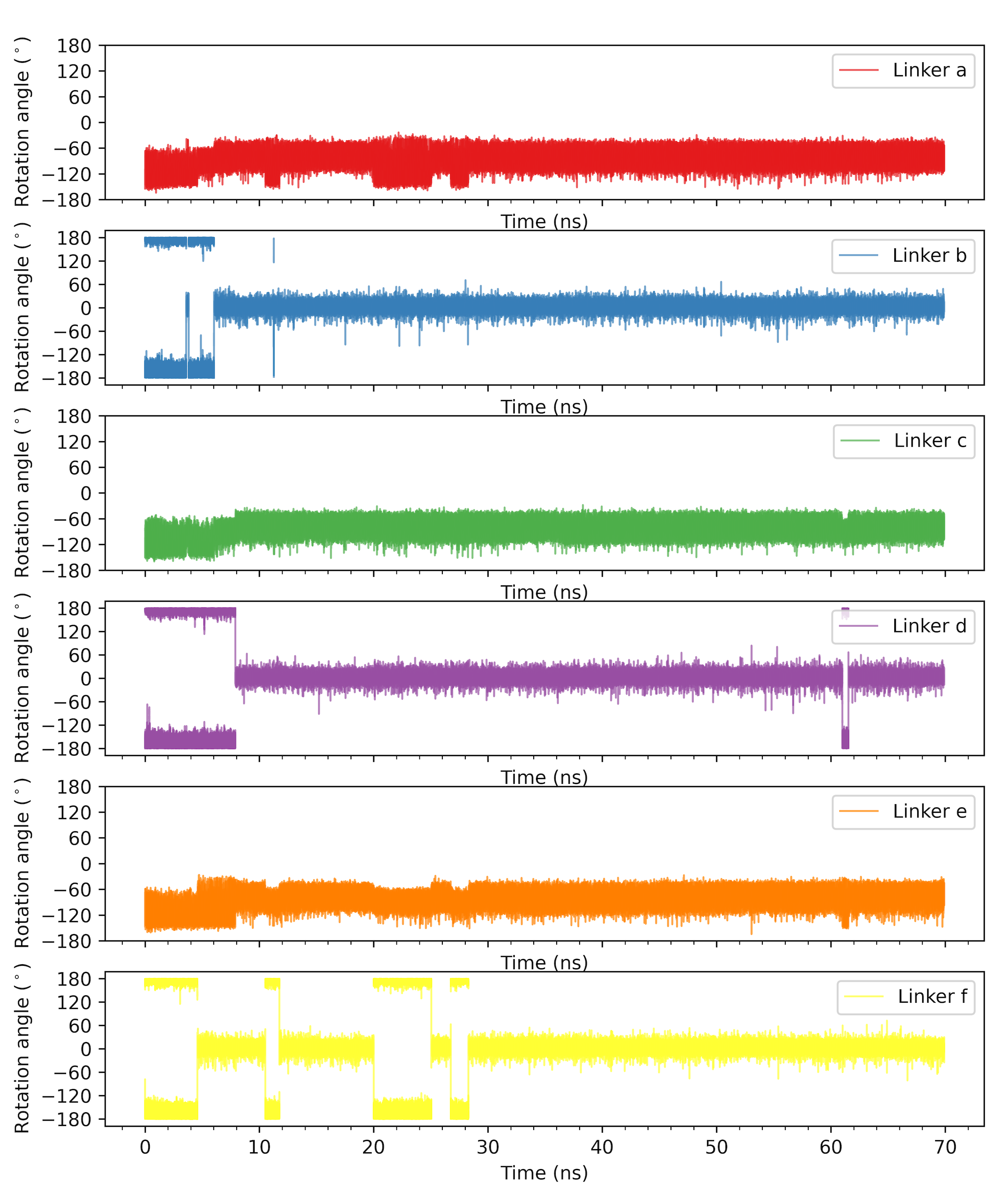

Supplement: Supplementary file 3 — jp4c05851_si_003.zip [file jp4c05851_si_003.zip › Trajectoryplots/622 supercell/622_Chain5.png]

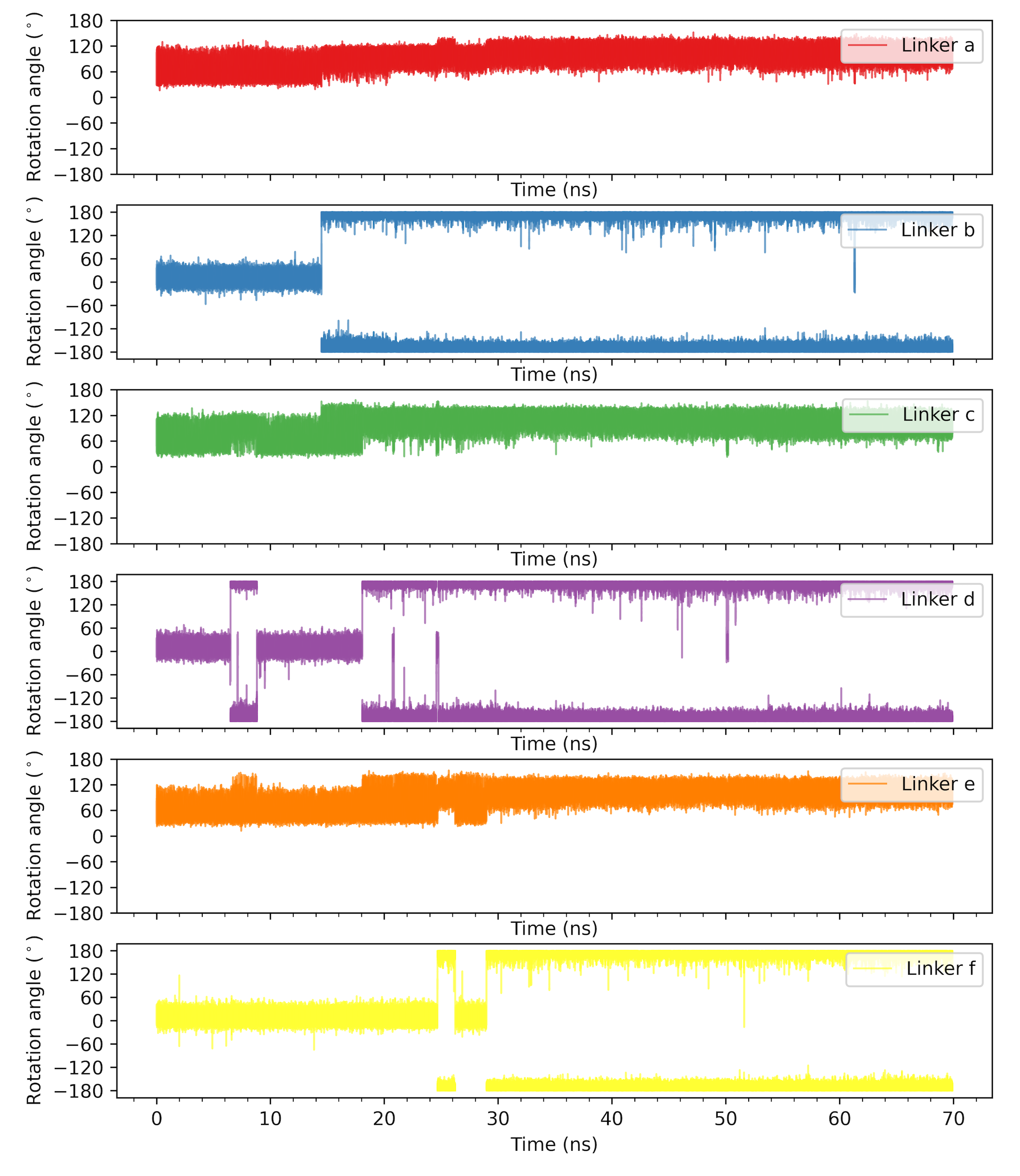

Supplement: Supplementary file 3 — jp4c05851_si_003.zip [file jp4c05851_si_003.zip › Trajectoryplots/622 supercell/622_Chain1.png]

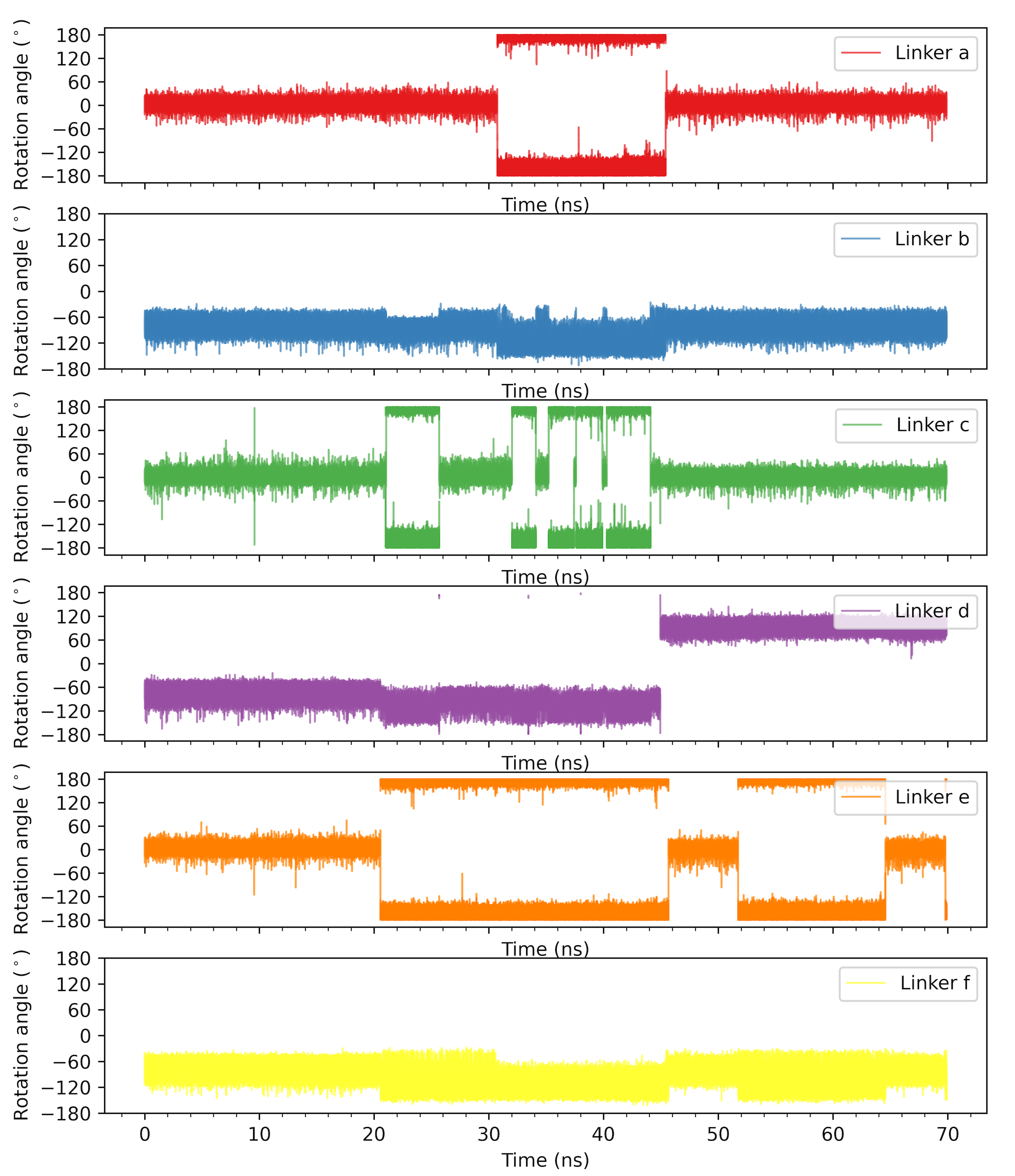

Supplement: Supplementary file 3 — jp4c05851_si_003.zip [file jp4c05851_si_003.zip › Trajectoryplots/622 supercell/622_Chain2.png]

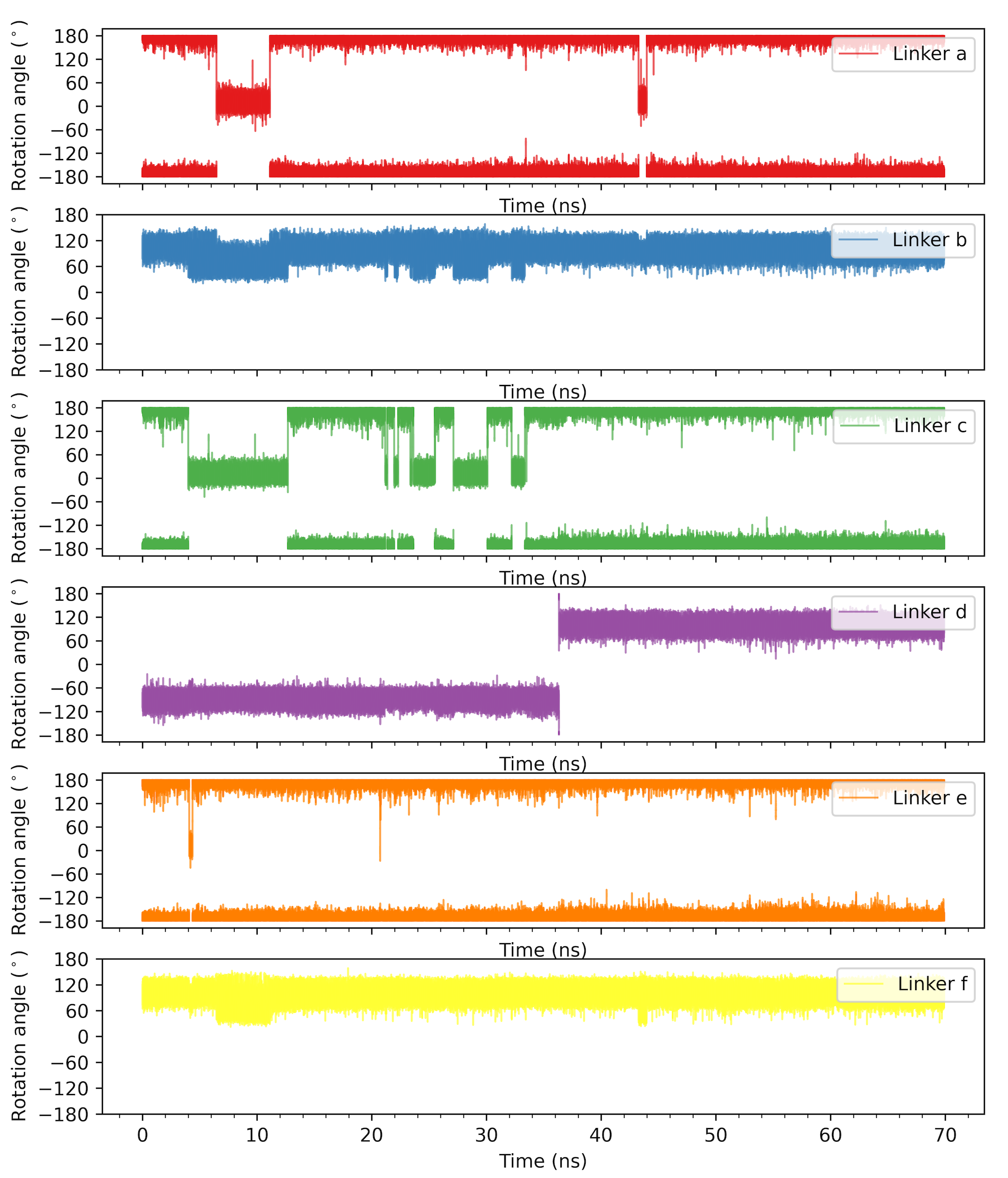

Supplement: Supplementary file 3 — jp4c05851_si_003.zip [file jp4c05851_si_003.zip › Trajectoryplots/622 supercell/622_Chain3.png]

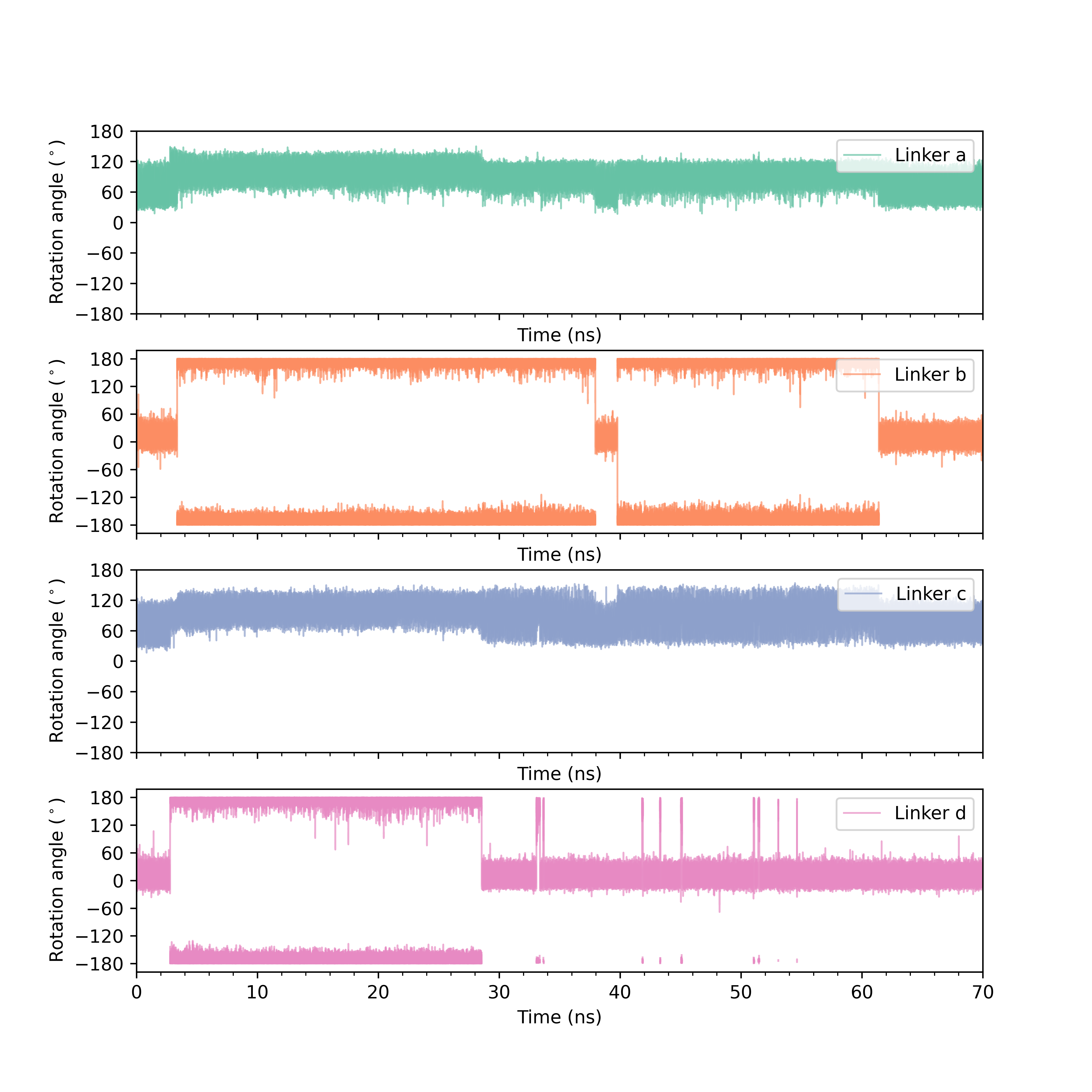

Supplement: Supplementary file 3 — jp4c05851_si_003.zip [file jp4c05851_si_003.zip › Trajectoryplots/422 supercell/422_Chain1.png]

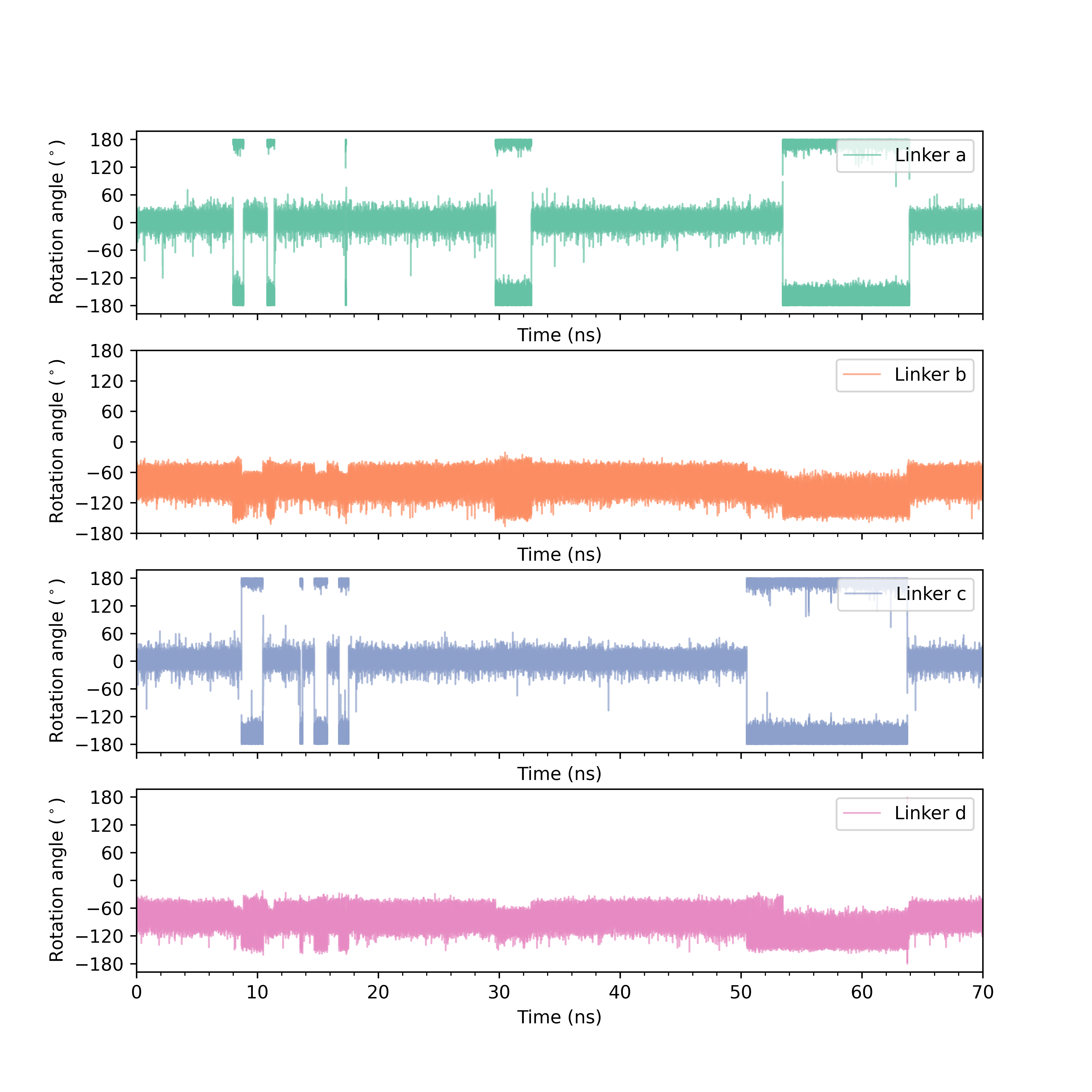

Supplement: Supplementary file 3 — jp4c05851_si_003.zip [file jp4c05851_si_003.zip › Trajectoryplots/422 supercell/422_Chain2.png]

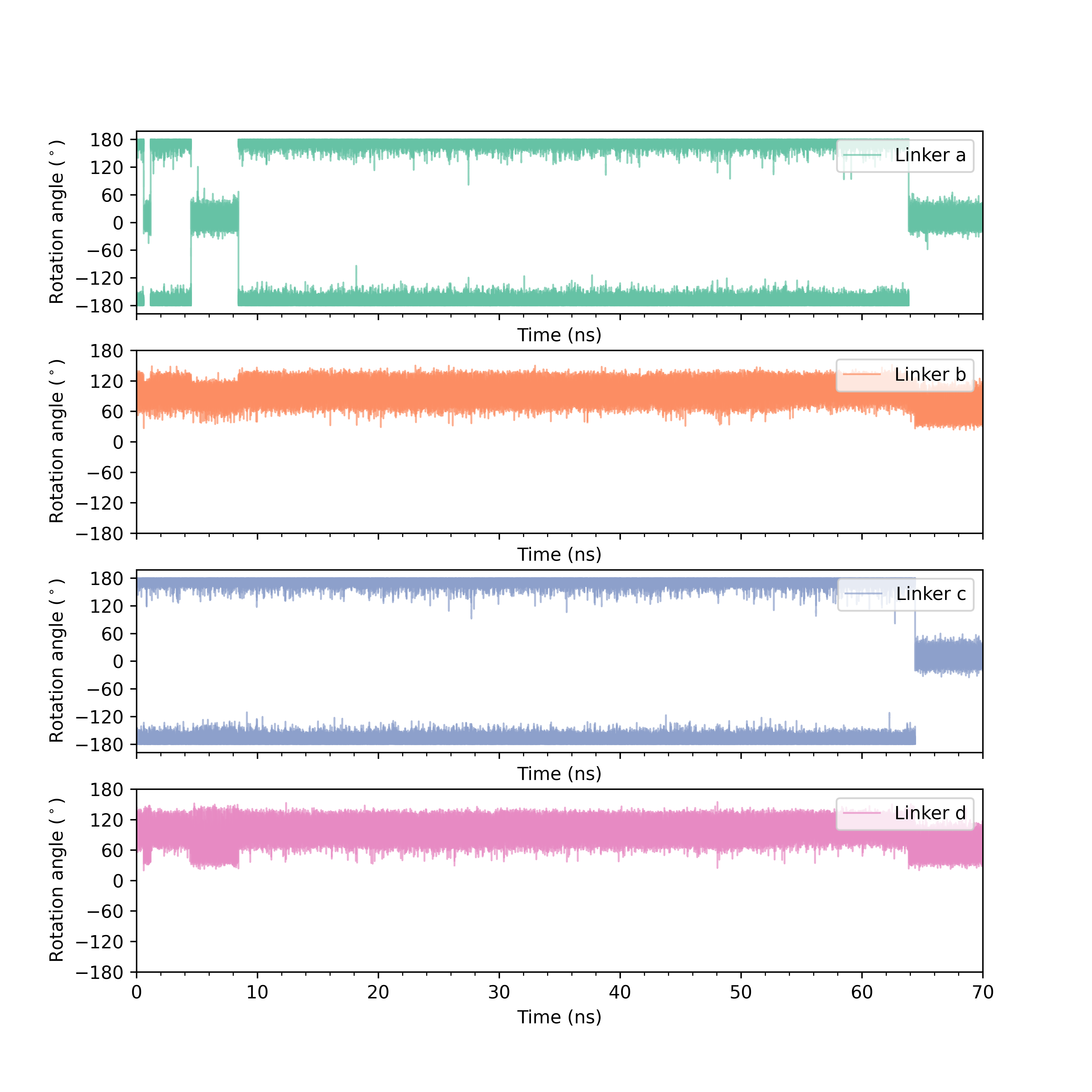

Supplement: Supplementary file 3 — jp4c05851_si_003.zip [file jp4c05851_si_003.zip › Trajectoryplots/422 supercell/422_Chain3.png]

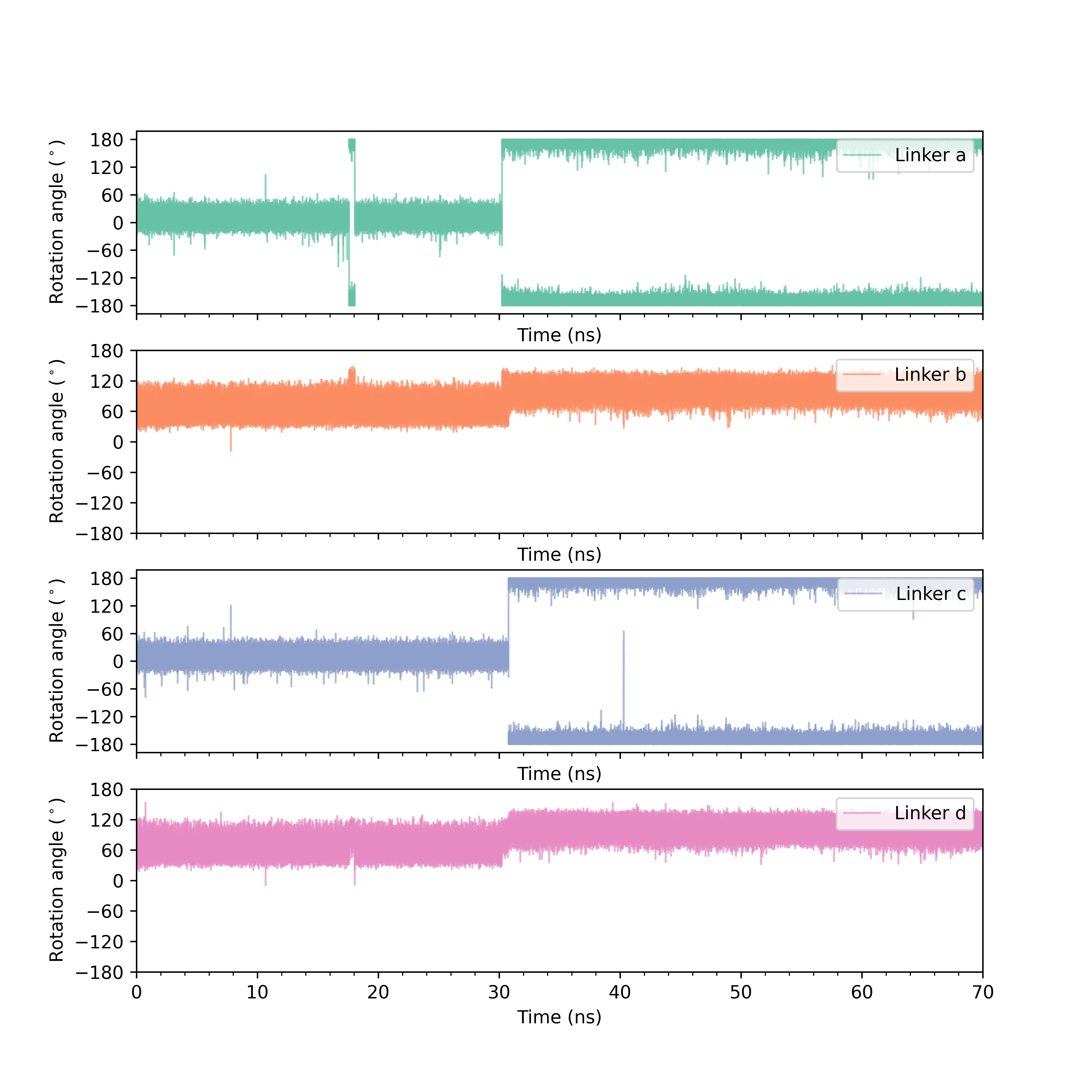

Supplement: Supplementary file 3 — jp4c05851_si_003.zip [file jp4c05851_si_003.zip › Trajectoryplots/422 supercell/422_Chain7.png]

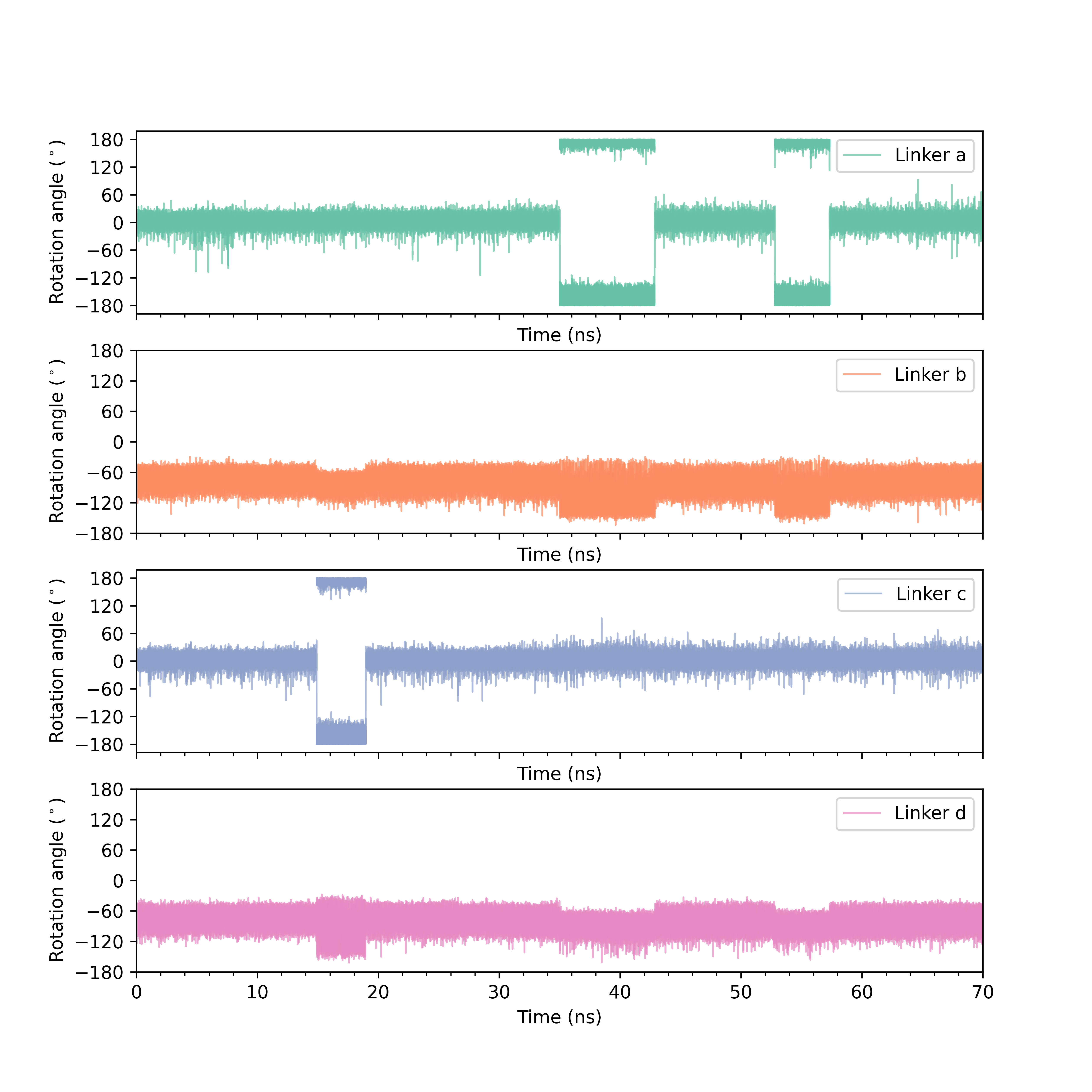

Supplement: Supplementary file 3 — jp4c05851_si_003.zip [file jp4c05851_si_003.zip › Trajectoryplots/422 supercell/422_Chain6.png]

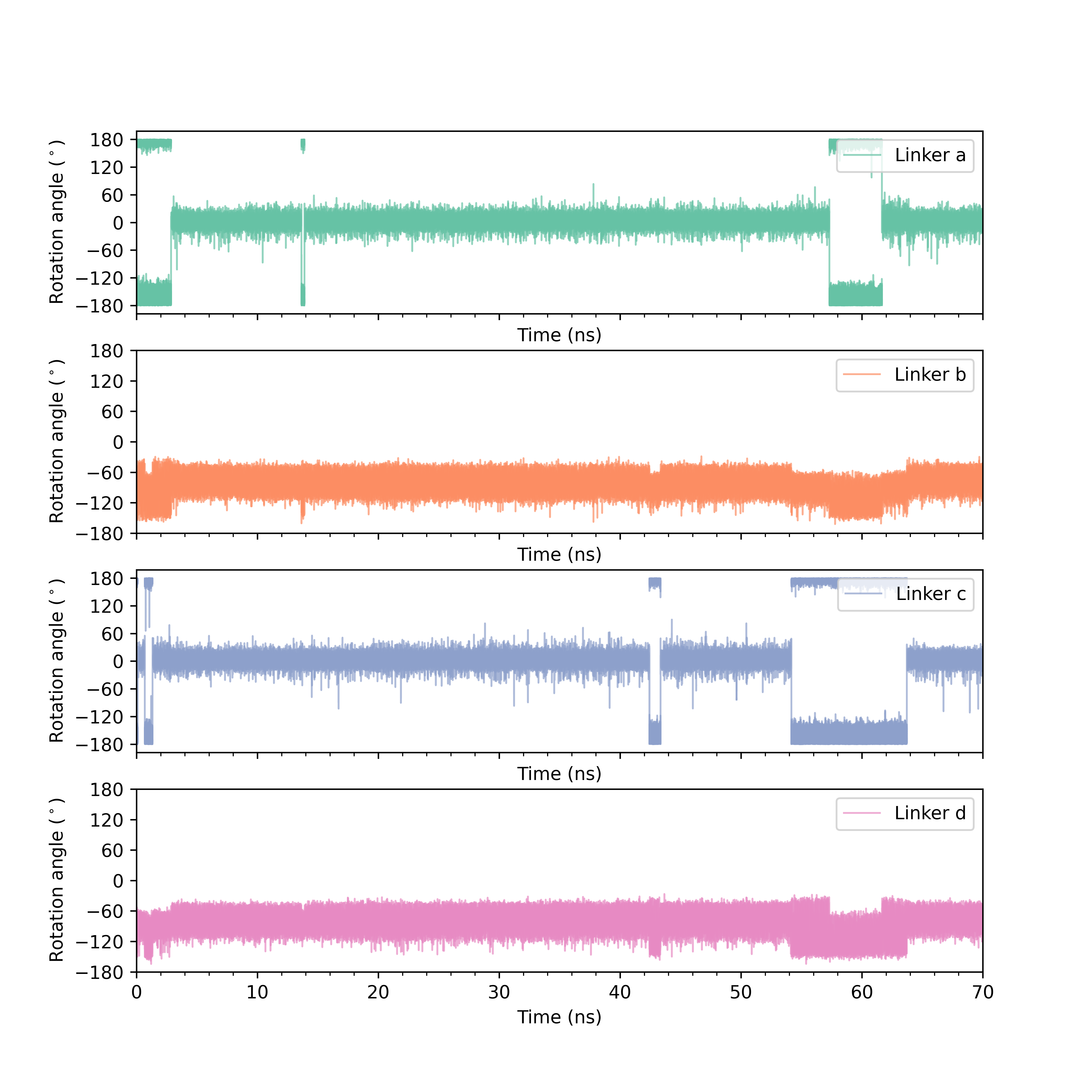

Supplement: Supplementary file 3 — jp4c05851_si_003.zip [file jp4c05851_si_003.zip › Trajectoryplots/422 supercell/422_Chain4.png]

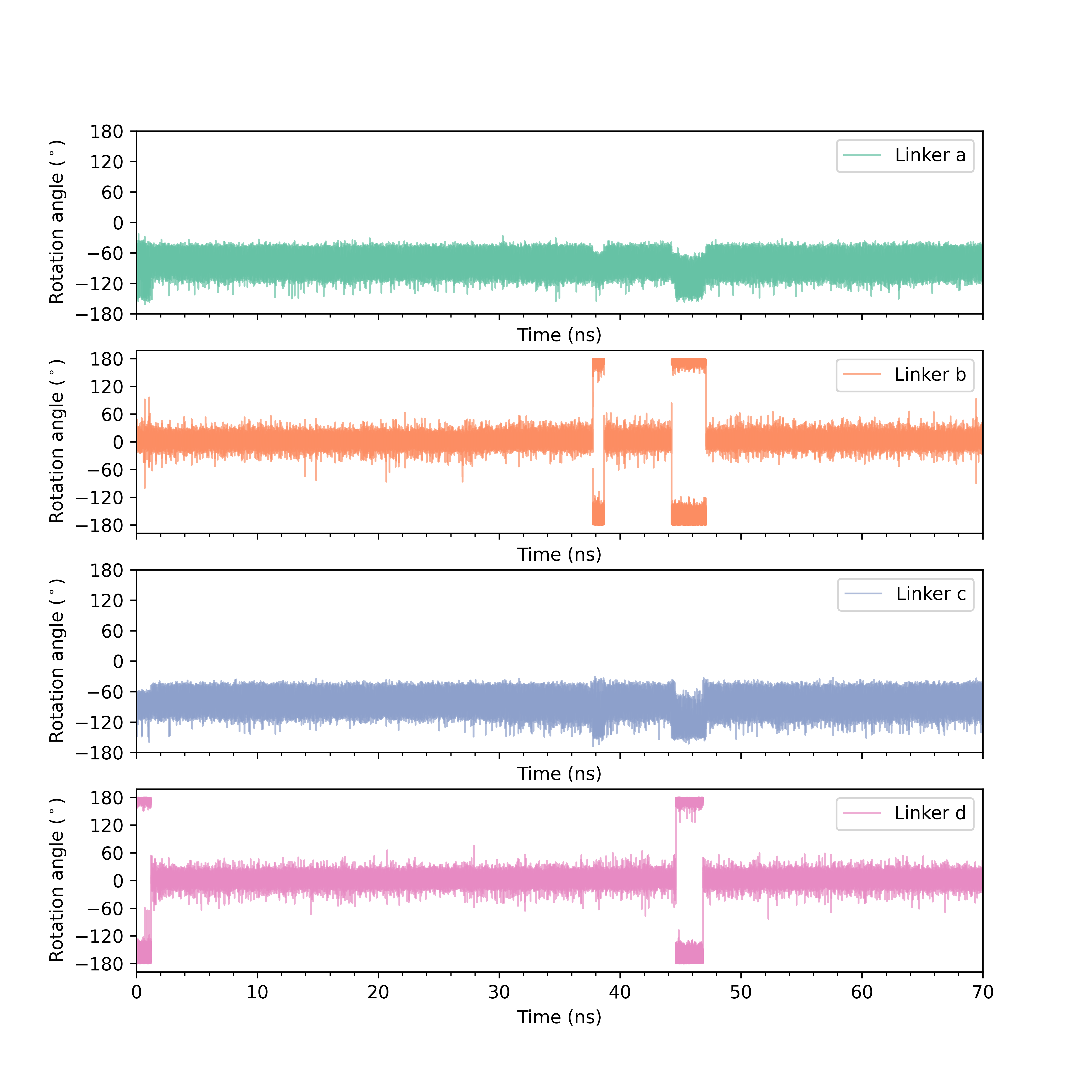

Supplement: Supplementary file 3 — jp4c05851_si_003.zip [file jp4c05851_si_003.zip › Trajectoryplots/422 supercell/422_Chain5.png]

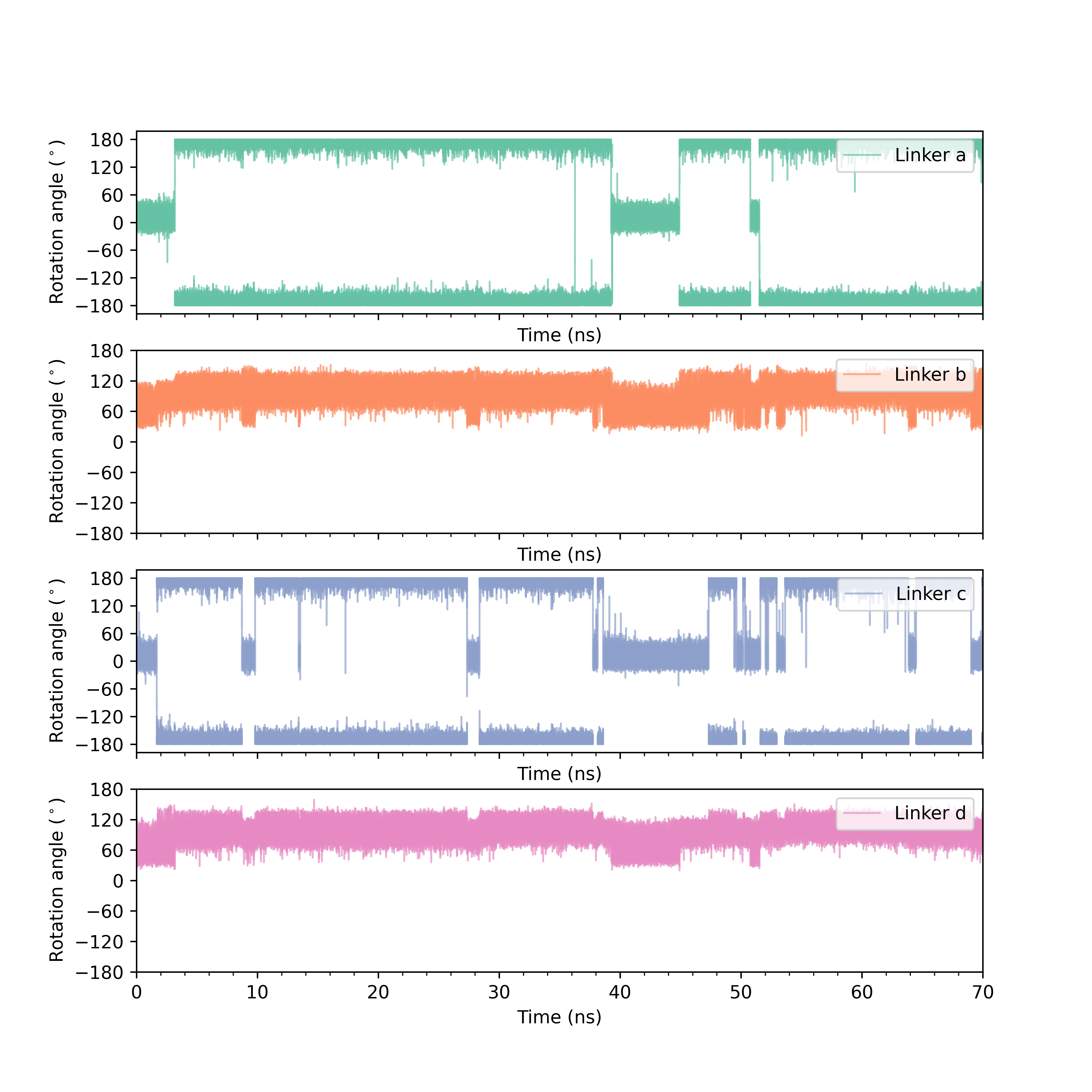

Supplement: Supplementary file 3 — jp4c05851_si_003.zip [file jp4c05851_si_003.zip › Trajectoryplots/422 supercell/422_Chain14.png]

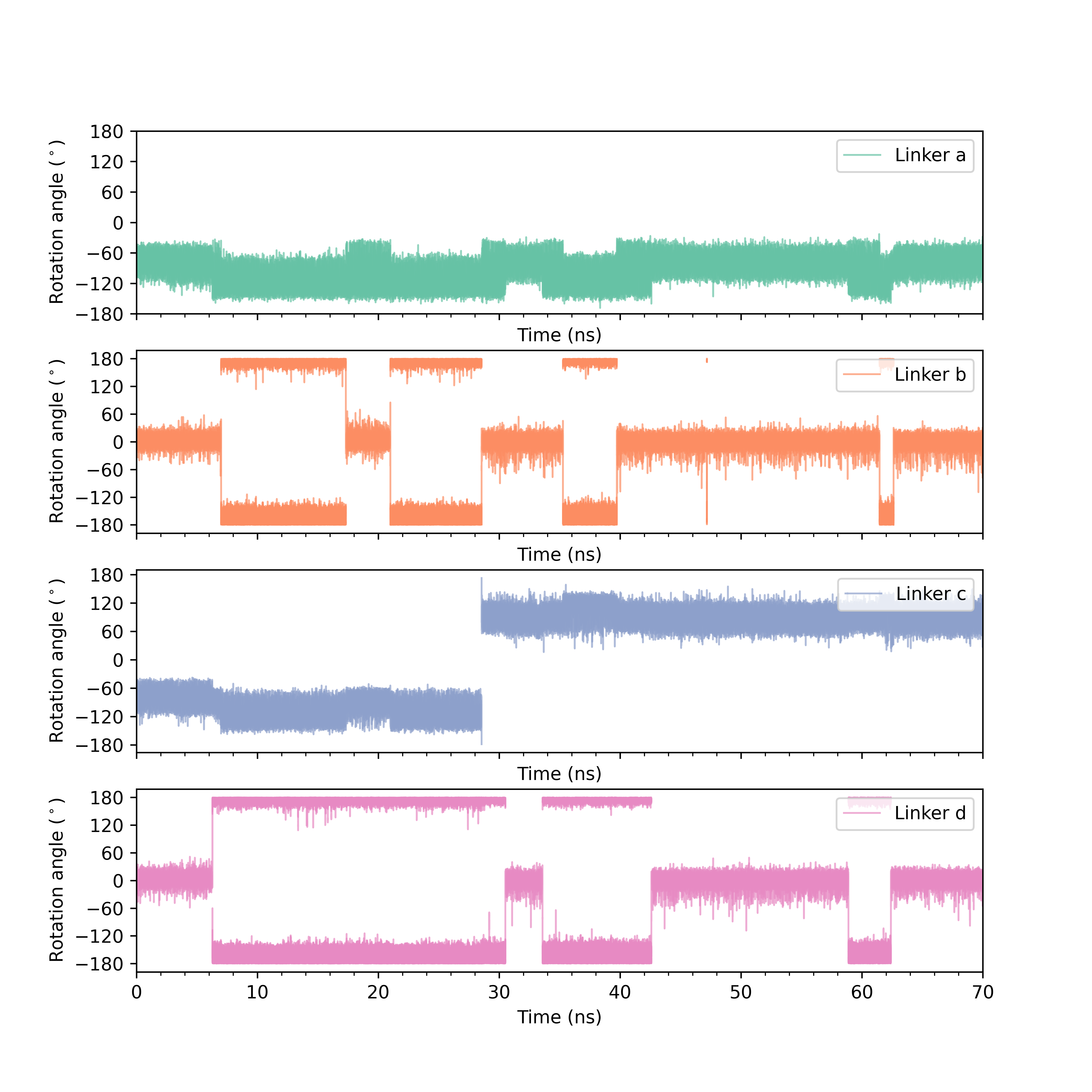

Supplement: Supplementary file 3 — jp4c05851_si_003.zip [file jp4c05851_si_003.zip › Trajectoryplots/422 supercell/422_Chain15.png]

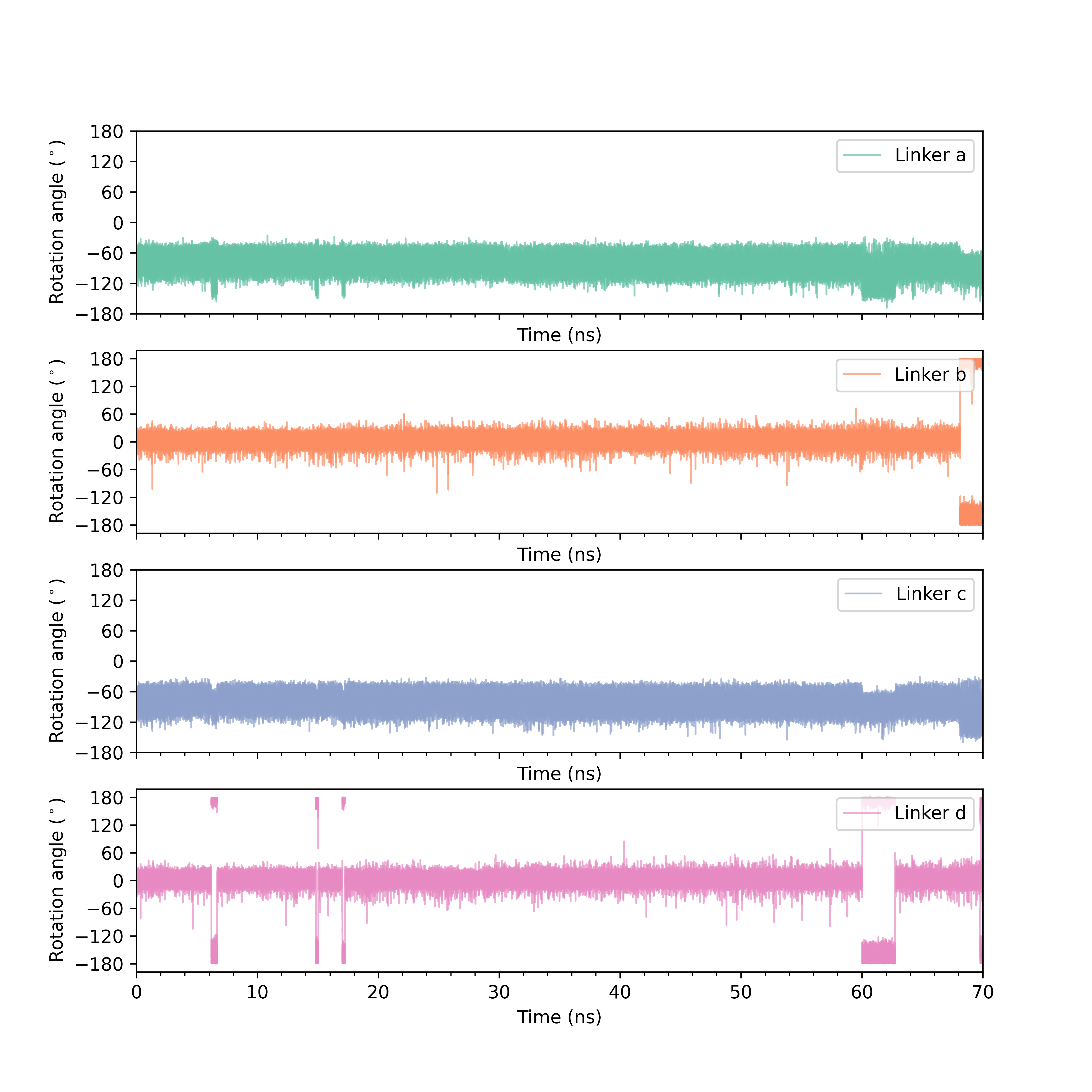

Supplement: Supplementary file 3 — jp4c05851_si_003.zip [file jp4c05851_si_003.zip › Trajectoryplots/422 supercell/422_Chain16.png]

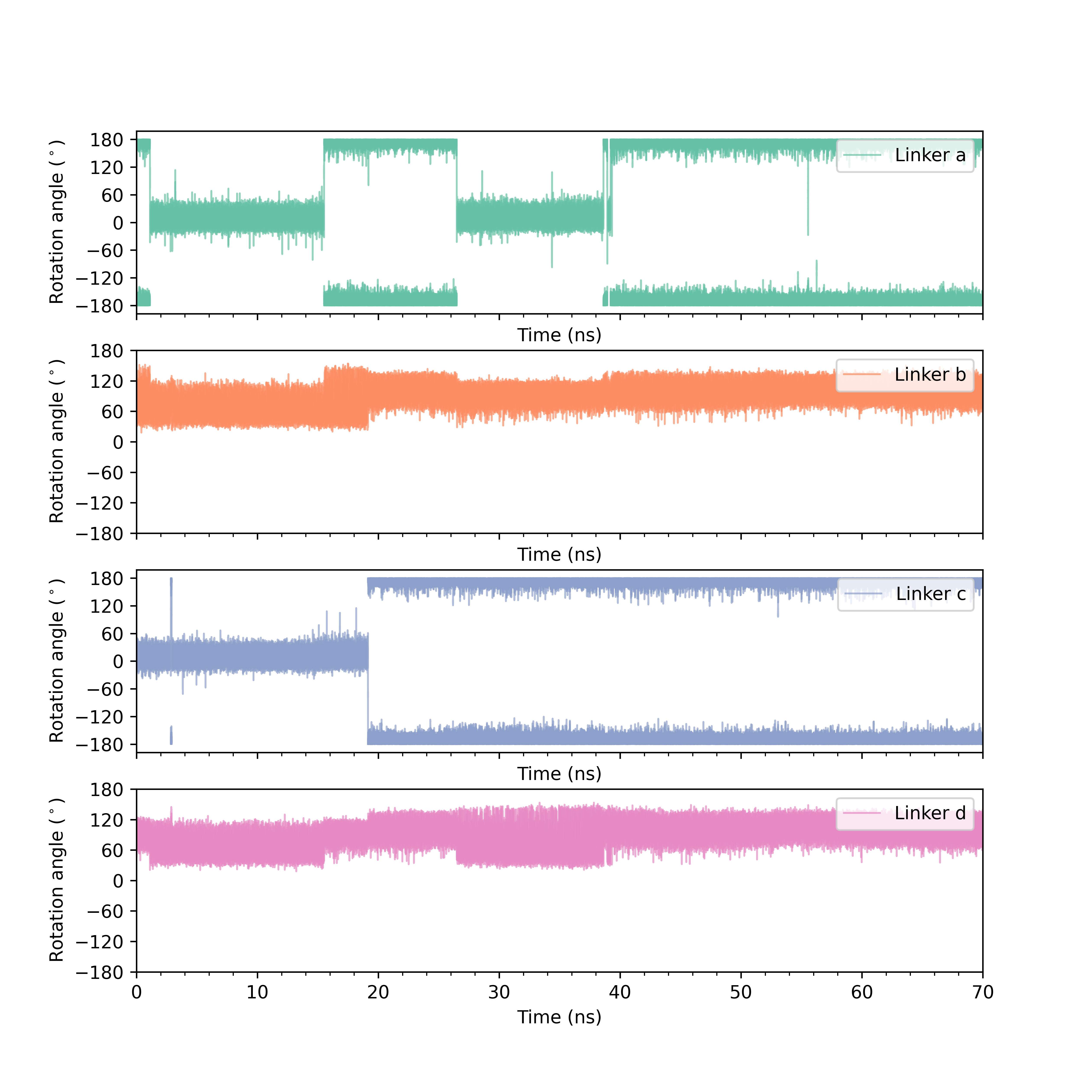

Supplement: Supplementary file 3 — jp4c05851_si_003.zip [file jp4c05851_si_003.zip › Trajectoryplots/422 supercell/422_Chain12.png]

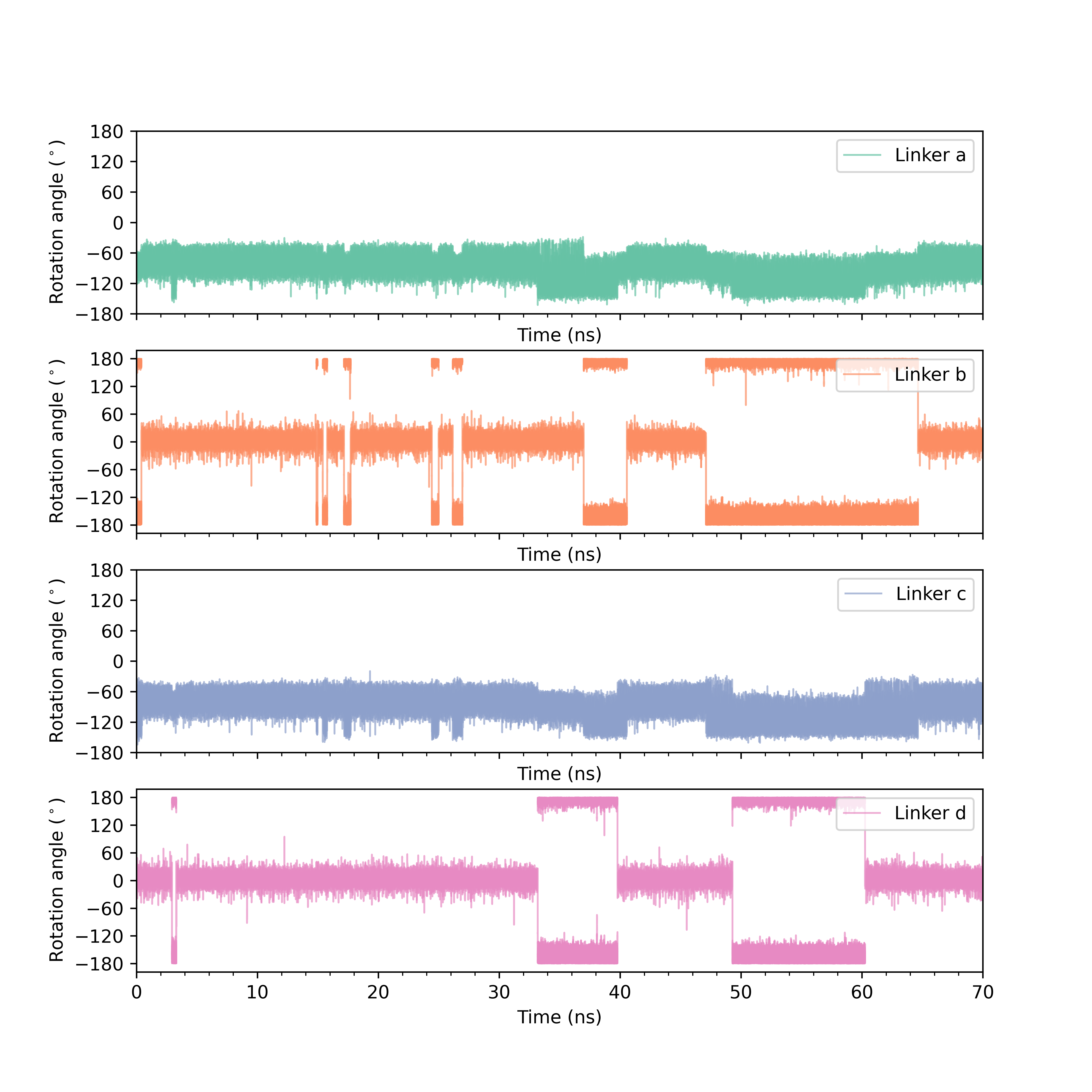

Supplement: Supplementary file 3 — jp4c05851_si_003.zip [file jp4c05851_si_003.zip › Trajectoryplots/422 supercell/422_Chain13.png]

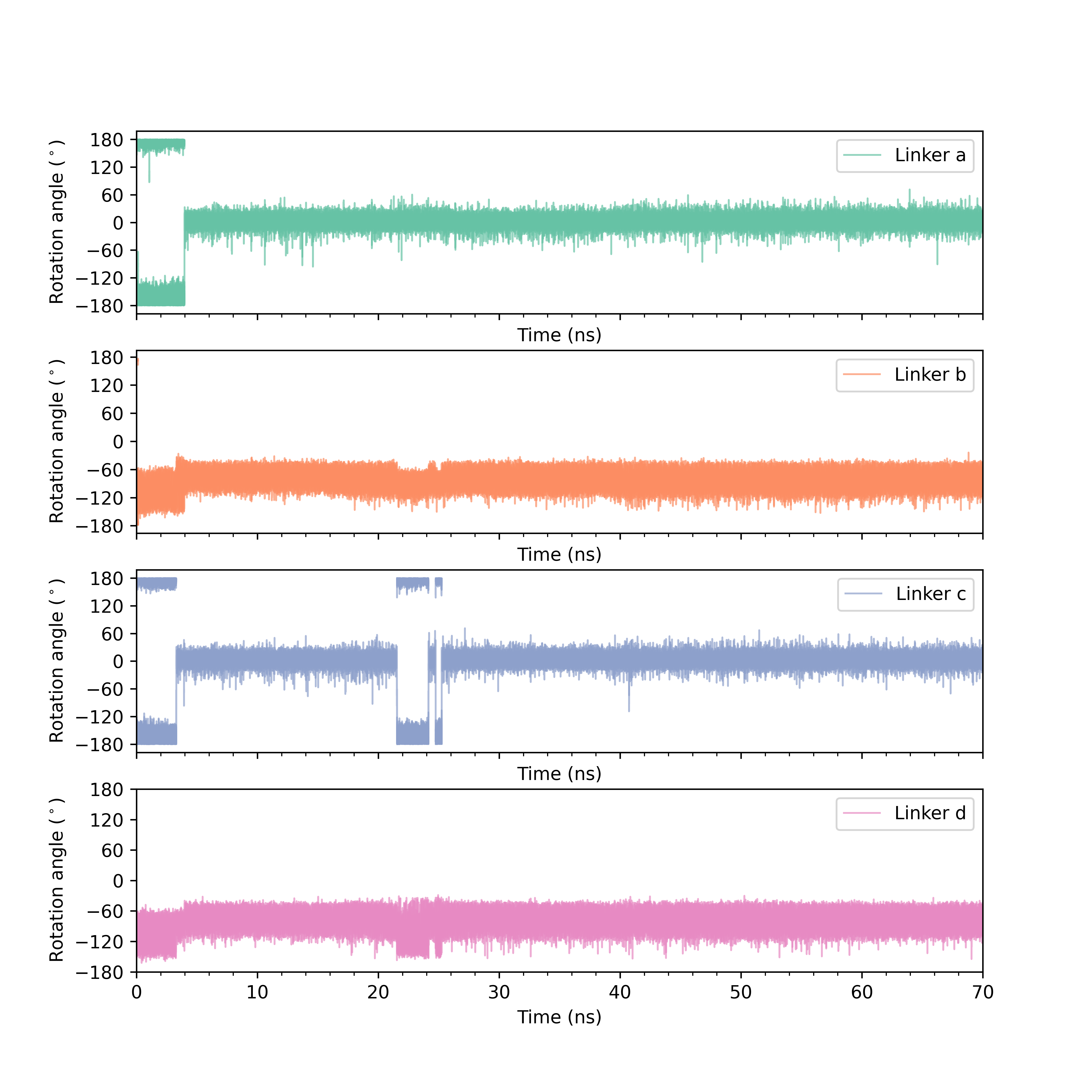

Supplement: Supplementary file 3 — jp4c05851_si_003.zip [file jp4c05851_si_003.zip › Trajectoryplots/422 supercell/422_Chain11.png]

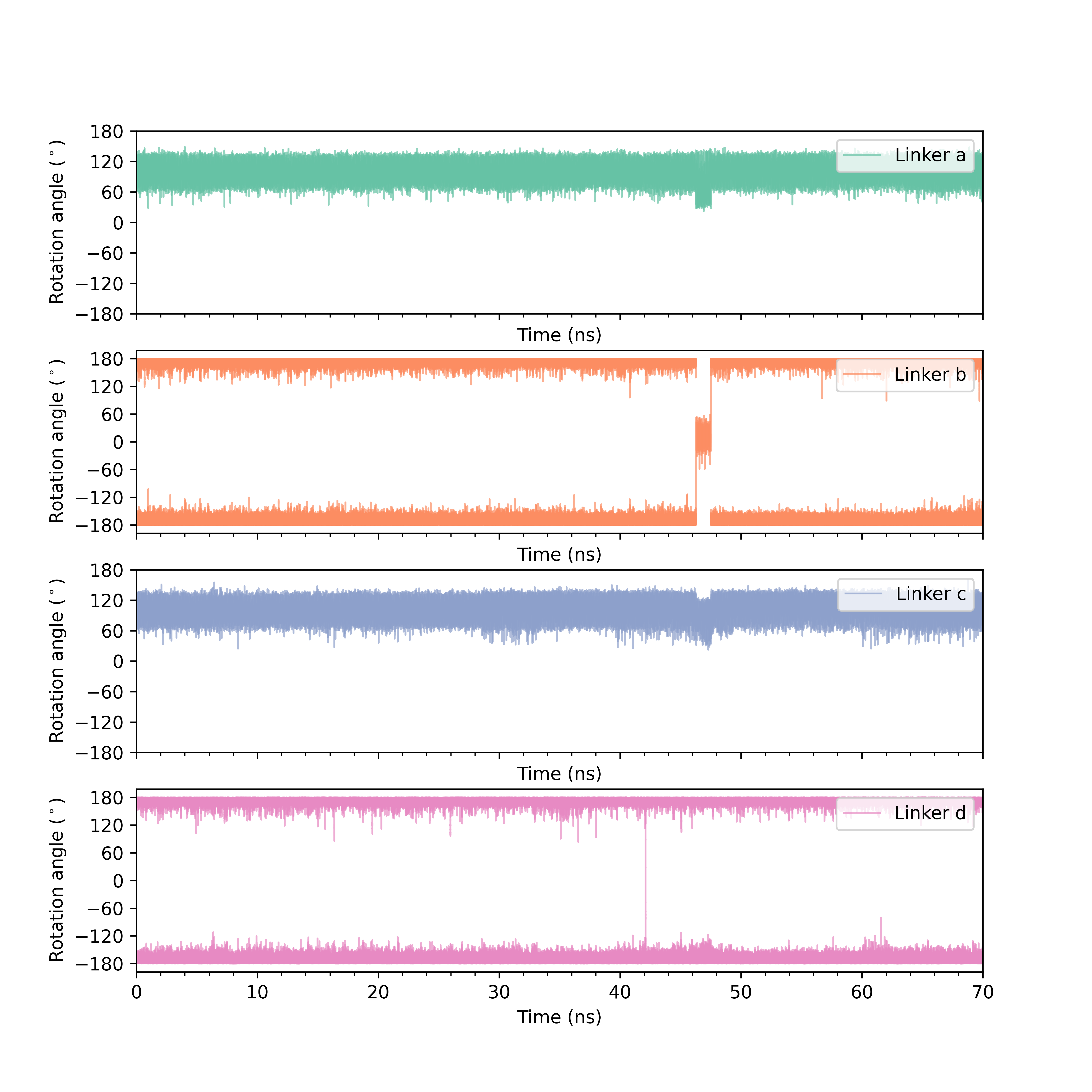

Supplement: Supplementary file 3 — jp4c05851_si_003.zip [file jp4c05851_si_003.zip › Trajectoryplots/422 supercell/422_Chain10.png]

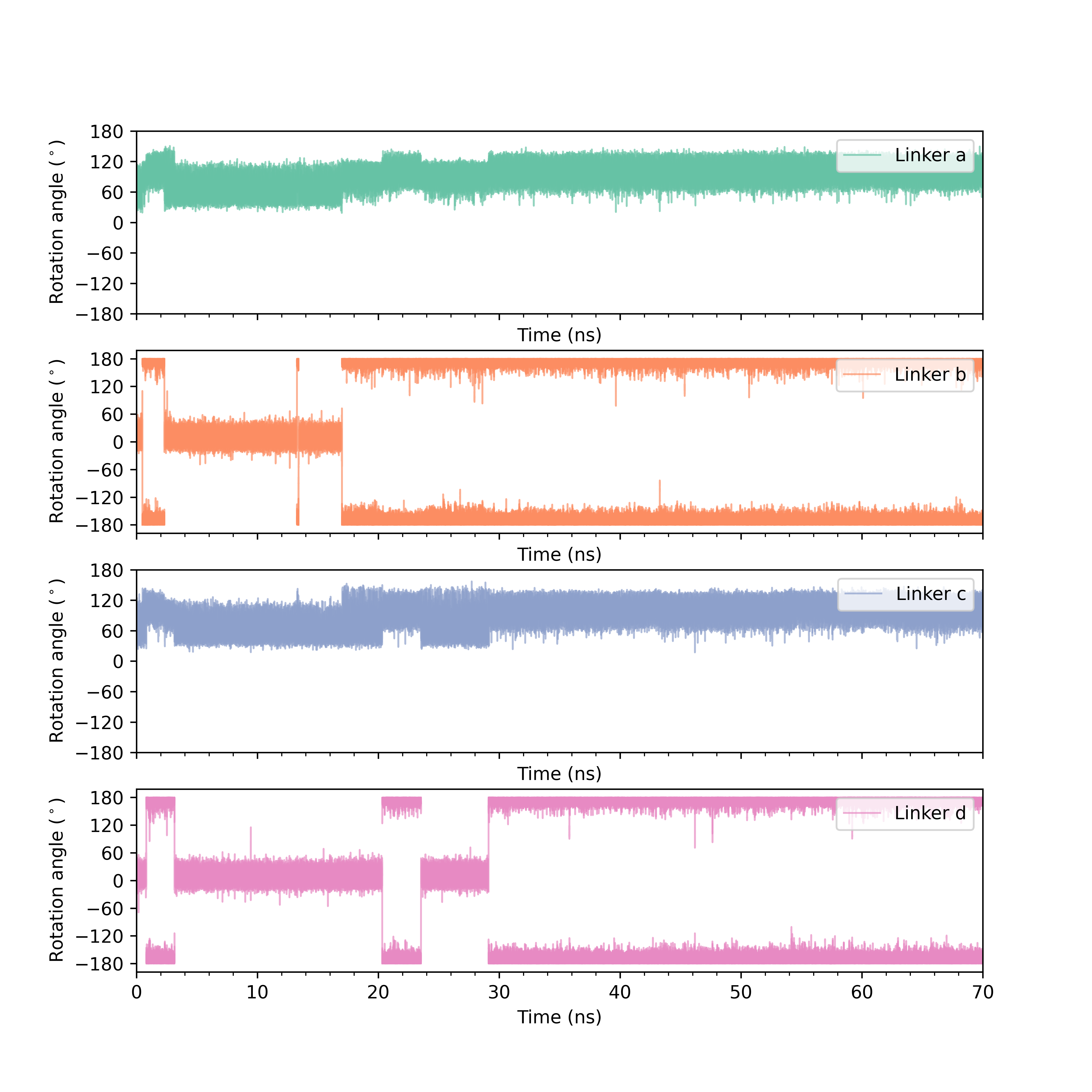

Supplement: Supplementary file 3 — jp4c05851_si_003.zip [file jp4c05851_si_003.zip › Trajectoryplots/422 supercell/422_Chain8.png]

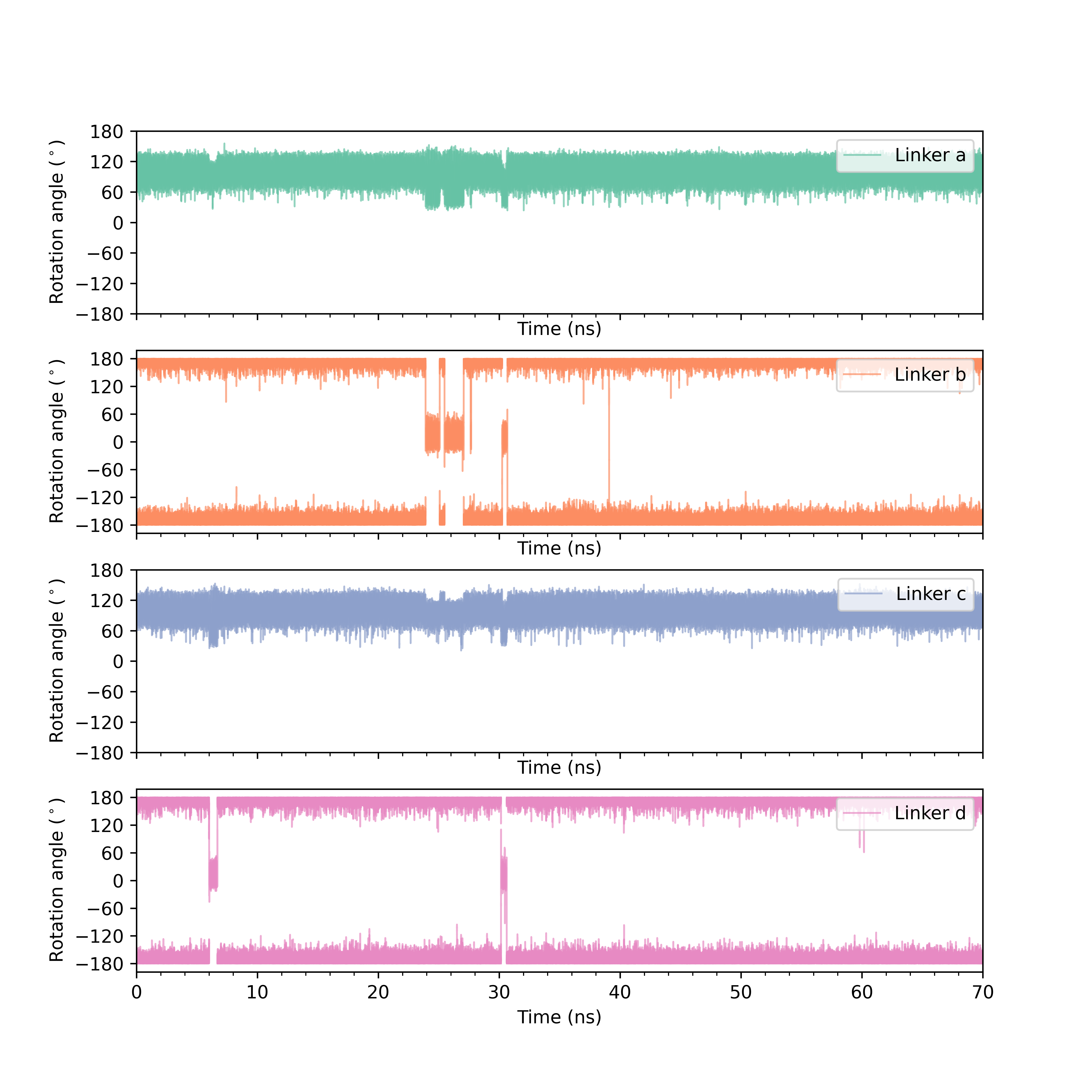

Supplement: Supplementary file 3 — jp4c05851_si_003.zip [file jp4c05851_si_003.zip › Trajectoryplots/422 supercell/422_Chain9.png]
